# Selecting auditory alerting stimuli for eagles on the basis of auditory evoked potentials

**DOI:** 10.1093/conphys/coac059

**Published:** 2022-09-16

**Authors:** Benjamin Goller, Patrice Baumhardt, Ernesto Dominguez-Villegas, Todd Katzner, Esteban Fernández-Juricic, Jeffrey R Lucas

**Affiliations:** Department of Biological Sciences, Purdue University, West Lafayette, IN 47907, USA; Department of Biological Sciences, Purdue University, West Lafayette, IN 47907, USA; Wellesley Animal Hospital, Richmond, VA 23233, USA; U.S. Geological Survey, Forest & Rangeland Ecosystem Science Center, 230 N Collins Rd., Boise, ID 83702, USA; Department of Biological Sciences, Purdue University, West Lafayette, IN 47907, USA; Department of Biological Sciences, Purdue University, West Lafayette, IN 47907, USA

## Abstract

Development of wind energy facilities results in interactions between wildlife and wind turbines. Raptors, including bald and golden eagles, are among the species known to incur mortality from these interactions. Several alerting technologies have been proposed to mitigate this mortality by increasing eagle avoidance of wind energy facilities. However, there has been little attempt to match signals used as alerting stimuli with the sensory capabilities of target species like eagles. One potential approach to tuning signals is to use sensory physiology to determine what stimuli the target eagle species are sensitive to even in the presence of background noise, thereby allowing the development of a maximally stimulating signal. To this end, we measured auditory evoked potentials of bald and golden eagles to determine what types of sounds eagles can process well, especially in noisy conditions. We found that golden eagles are significantly worse than bald eagles at processing rapid frequency changes in sounds, but also that noise effects on hearing in both species are minimal in response to rapidly changing sounds. Our findings therefore suggest that sounds of intermediate complexity may be ideal both for targeting bald and golden eagle hearing and for ensuring high stimulation in noisy field conditions. These results suggest that the sensory physiology of target species is likely an important consideration when selecting auditory alerting sounds and may provide important insight into what sounds have a reasonable probability of success in field applications under variable conditions and background noise.

## Teaser

One potential way to keep bald and golden eagles away from wind turbines is to develop alerting stimuli. We measured eagle hearing to determine which sounds they can hear well. We found that dynamic sounds of intermediate complexity best stimulated bald and golden eagle hearing in noisy environments.

## Introduction

The U.S. Department of Energy envisions a future where the percentage of national electricity demand supplied by wind energy increases from 4.5% in 2013 to 20% by 2030 and 35% by 2050 (U.S. Department of Energy, 2015, 2018). These scenarios require continued investment in new wind energy facilities and therefore represent an increased potential for interactions involving wind turbines and bird and bat species, some of which are negatively affected by wind energy facilities (American Wind Wildlife Institute, 2017; American Wind Wildlife Institute, 2019). Among the bird species found in energy facility conservation surveys, golden eagles and other diurnal raptors account for ~8% of carcasses (American Wind Wildlife Institute, 2019). American kestrels (3.1%) and red-tailed hawks (2.9%) make up the majority of the diurnal raptor mortalities, but these species also have large population sizes and are widespread across the USA. Golden eagles account for only a handful of raptor mortalities, but have relatively small population sizes (U. S. Fish and Wildlife Service, 2016; Millsap *et al.*, 2022), and are therefore a high conservation priority around wind energy facilities built in potential eagle habitat.

Despite efforts to site wind energy projects with conservation in mind, some energy facilities, or even single turbines, can cause significant levels of eagle mortality (e.g. Smallwood and Thelander, 2008; Carrete *et al.*, 2009; Thaker *et al.*, 2018). Thus, in addition to careful siting, two additional types of solutions have been proposed to reduce the impact of wind turbines on susceptible bird species like golden eagles: (i) offset impact on species by supporting conservation efforts to increase populations of susceptible species and (ii) minimize turbine–animal interactions using technologies or strategies that prevent collisions (Allison *et al.*, 2017). One such turbine–animal interaction minimization solution is to decrease the danger posed by the turbines by stopping or curtailing the movement of turbine rotors when birds are near enough to be at risk. Curtailment can be highly effective when implemented with sufficient time for the rotors to stop (de Lucas *et al.*, 2012; McClure *et al.*, 2021), but at the cost of a reduction in energy production (de Lucas *et al.*, 2012; Singh *et al.*, 2015).

Instead of stopping the turbine, an alternative strategy could be to reduce the time an animal spends near a turbine. Removing attractants such as nesting sites, food sources or flight corridors could help to prevent animals from approaching wind energy facilities. Another solution is to keep the animals away from the wind energy facility using targeted auditory or visual stimuli (Khan, 2014; Gradolewski *et al.*, 2021). Wind turbines themselves produce sounds, in particular fairly broad-band noise (Schäffer *et al.*, 2016) that has an amplitude modulation rate of 2–4 Hz (Hafke-Dys *et al.*, 2016). While it may seem obvious that eagles should be able to see a wind turbine from a fairly long distance, there are at least three reasons why sound alerting stimuli may be especially effective at reducing eagle mortality at wind turbines. First, both eagle species have fairly extensive blind areas above their heads (Fernández-Juricic et al., 2020), with the blind area of golden eagles nearly twice as wide as the blind area for bald eagles (74° vs. 44°, respectively). This is relevant because an eagle scanning the ground may not be able to see an object in front of it, even if that object was as large as a turbine. The relevance of the problem lies in part from the fact that birds in flight, and especially eagles, may naturally predict that the environment ahead of them is uncluttered (Martin, 2011). The colour of the spinning turbine may also make the turbine difficult to see (May *et al.*, 2020). Moreover, there would presumably be only weak selection for a top predator such as an eagle to process such information. Second, the complexity of the sound environment may also contribute to turbine-induced mortality. Birds have very good hearing (Dooling and Fay, 2000), but the sounds emanating from the turbines do not have acute onset or offset cues that would facilitate aurally locating the source (Grothe *et al.*, 2010). This is particularly problematic in large turbine facilities because low frequency turbine noise coming from multiple directions can be audible (to humans at least) up to 4 km from the source (Zajamšek *et al.*, 2016), making a single turbine even more difficult to locate aurally. In addition, the problem of detecting the direction of turbines could exacerbate the problem associated with the large blind spot above the head. Third, the passive use of updrafts by large raptors during soaring and gliding flight may carry a bird into a turbine (Barrios and Rodriguez, 2004; Miller *et al.*, 2014). As such, alerting technologies based on well-designed sounds with acute onsets could provide immediate localizable information about the location of a nearby turbine and thereby offer a solution that avoids stoppages in energy production.

One potential way to maximize the effectiveness of alerting technology could be to tune alerting stimuli to the sensory capabilities of the target animals (Goller *et al.*, 2018; reviewed in Blackwell and Fernández-Juricic, 2013). Studying the sensory physiology of a target species can provide insight into the types of signals that stimulate the sensory systems of those animals. Specifically, it is important to find signals that can alert the target animal in natural and behaviourally relevant scenarios, such as noisy field conditions (Gomes *et al.*, 2016; Akcay and Beecher, 2019). For example, narrow band noise covering frequencies where songbirds are known to be sensitive has been shown to reduce risk of avian collision on human-built structures (the ‘acoustic lighthouse’ of Boycott *et al.*, 2021), and ultrasonic noise that masks echolocation clicks has been shown to be a useful deterrent for bats (Gilmour *et al.*, 2020). In addition to being highly alerting, carefully selected sounds may decrease the likelihood of animals habituating to the sound signals used (see Baxter and Robinson, 2007; Götz and Janik, 2015).

We investigated the potential for a variety of different sounds to be processed by the eagle auditory brainstem, with the goal of finding effective alerting sounds. Auditory Evoked Potentials (AEPs; Hall, 2007) measured in response to a suite of simple and complex sound stimuli might provide insight into behaviourally relevant processing of sounds and therefore serve as an important benchmark for selecting candidate sounds for implementation in wind turbine alerting technologies. Specifically, we measured AEPs of bald and golden eagles using a variety of different sounds and background noise conditions to (i) determine how well the auditory brainstem of each species processes a suite of different candidate sounds and (ii) measure how these eagle auditory responses are affected by background noise. These measurements allow us to rapidly collect information about the peripheral auditory processing of eagles without having to sacrifice animals. Our approach was to broadly survey eagle hearing performance to increase the chance of finding valuable candidate stimuli to use as alerting sounds. We therefore measured AEPs to a range of stimuli including pure tones, harmonic stacks of tones, amplitude-modulated (AM) sounds and frequency-modulated (FM) sounds. Each stimulus was tested against three different noise treatments (no noise, pink noise and white noise, as detailed below) to evaluate how the processing of the stimuli is resistant to noise masking.

Note that AEPs have been validated in several ways. Some studies have shown strong correlations between AEPs and single-cell neuronal responses to sound (Henry, 2002; Pienkowski and Harrison, 2005). Audiograms (functions relating minimal intensities that generate an auditory response as a function of frequency) have been generated using both auditory brainstem response (ABR) and behaviour (i.e. perception of sounds) for a number of species (beaked whales, Cook *et al.*, 2006; dolphins, Finneran and Houser, 2006; false killer whales, Yuen *et al.*, 2005; budgerigars, Brittan-Powell *et al.*, 2002; parakeets, Dooling and Walsh, 1976, Saunders *et al.*, 1980). Audiograms generated from AEPs invariably show higher thresholds than behavioural audiograms, but the relative shape of the audiograms is preserved, suggesting that AEPs provide robust information about frequency sensitivity. In fact, when AEPs to speech are amplified and converted to sounds, the sounds can be understood as the original speech patterns (Galbraith *et al.*, 1995). As with audiograms, more complex processing of sound as indexed with AEPs has been validated using correlations with behavioural measures. For example, auditory sensitivity to amplitude modulation measured using AEPs strongly correlates with behavioural sensitivity values in owls and starlings (Dent *et al.*, 2002), dolphins (Supin and Popov, 1995) and humans (Eddins, 1993). Pienkowski and Harrison (2005) showed close correspondence between AEPs and cortical neuronal responses to tones. Finally, Furst *et al.* (1990) showed that AEPs contain information about interaural time differences and about interaural intensity level differences in humans. AEPs strongly correlate with behaviour on these tasks. In short, AEPs provide a robust measure of auditory processing.

## Methods

### Subjects

We measured AEPs of a total of six bald eagles (*Haliaeetus leucocephalus*) and two golden eagles (*Aquila chrysaetos*). Five bald eagles (two juveniles younger than 5 years and three adults) were tested at the Wildlife Center of Virginia in Waynesboro, VA, USA (April–September 2018) and one adult bald eagle and two golden eagles (one juvenile younger than 5 years and one adult) were tested at Liberty Wildlife in Phoenix, AZ, USA (February 2019). These eagles were all rehabilitated wild eagles that had regained healthy status and had completed their clinical treatment. They were all in their final stages of rehabilitation towards the goal of release back into the wild, so our sample size was limited by the number of animals meeting these strict criteria during the period of this study. Access to the eagles and running the experiments were at the discretion of, and in collaboration with, the veterinary staff of each rehabilitation facility. Subject eagles were carefully monitored throughout the experiment. All work with the eagles was conducted with approval of the Purdue Animal Care and Use Committee (PACUC Protocol #: 1705001579) as well as U.S. Fish and Wildlife (Permit #: MB41892B-1) and state authorities of Virginia (Permit #: 62486) and Arizona (Permit #: SP638641).

### Anaesthesia

For the AEP measurements, the eagles were fully anaesthetized with injectable and inhaled anaesthesia. The mixture was necessary because higher amounts of inhaled isoflurane commonly used in veterinary procedures can attenuate the peripheral auditory system responses (see Thiele and Köppl, 2018). Eagles were initially anaesthetized with an intramuscular injection of 0.20 mg/kg butorphanol, 0.40 mg/kg midazolam and 0.08 mg/kg dexmedetomidine. Isoflurane (1%) was then administered, using a mask that covered the bill, as necessary to prepare the eagle for intubation, followed by insertion of an intravenous (IV) or intraosseous (IO) catheter on the right leg of the bird.

Once intubated and catheterized, the eagle was moved to a Faraday cage housed in an anechoic chamber (see below) and connected to a supply of oxygen and 0.25% isoflurane, as well as an IV. A low dose of isoflurane provided veterinarians with a means to rapidly respond to changes in eagle condition (the physiological response to injectable anaesthesia is relatively slower than the response to isoflurane) without hindering physiological measurements. An oesophageal stethoscope was used to monitor heart rate of the subject eagle from outside the anechoic chamber and a USB ‘night vision’ webcam provided a live visual update on the animal. The oxygen and isoflurane supplies for the intubation tube, a bag to allow for manual respiration if necessary, stethoscope ear pieces and IV line were all routed through openings in the Faraday cage (see below) and the anechoic chamber wall. This allowed the veterinarian to adjust anaesthesia and monitor animal condition from outside the closed anechoic chamber.

Experiments were designed to allow a stoppage every 30 mins for a top-up injection of half-doses of the injectable anaesthetic mixture. Top-ups were administered as necessary based on the recommendation of the monitoring veterinary staff in consultation with the researcher. AEPs are relatively weak signals. Therefore, large amplitude bursts of myogenic activity in the electrode recordings could be used as an indicator that the subject bird was no longer fully anaesthetized. The injectable half-dose was delivered via the IV/IO catheter and flushed with a mix of plasmalyte (4 ml/kg/hr), hetastarch (15 ml/kg) and normosol (10 ml/kg). Experiments resumed after the veterinarian indicated the subject eagle’s condition was stable after the fresh top-up of anaesthesia. The subject was given 0.25–0.5% isofluorane throughout the experiment to maintain stable heart rate and respiratory rate. The veterinarian was present and monitored the condition of the eagle throughout the procedure without opening the anechoic chamber.

At the end of the experiment, the eagle was quickly extracted from the experimental chamber and the intubation tube was removed. An atipemazole (0.40 mg/kg) injection was administered to speed up final recovery. The total procedure from induction to recovery was 3–3.5 h with 30 mins to 1 h of preparation (intubate, position, and ensure stable condition of the animal), 1.5 h of experiment trials in three 30-min blocks and 1 h recovery post-experiment.

### Auditory evoked potentials

#### Anechoic chamber

The anechoic chamber was a 1.22 × 1.22 × 1.22 m cube constructed of 3-mm-thick aluminium composite material sheets (Meyer Plastics Inc., Indianapolis, IN, USA) and 6061 aluminium 90° angle iron to connect and reinforce the edges. The chamber is lined with two layers of anechoic foam: first, a layer of 1.5 cm Fireflex flat panels of foam, then 7.6-cm-thick UNX-3 SONEX classic polyurethane foam. One side of the cube had a 0.91 × 0.91 m door that hinged at the top. Inside the chamber was a 0.81 × 0.81 × 0.46 m (l × w × h) Faraday cage made of 10-mesh copper wire mesh (wire diameter, 0.635 mm) over a wooden frame. The cage was electronically grounded to the ground of the RZ6 recorder (see below). The top half of the Faraday cage could be lifted off and removed from the chamber to allow easy setup of the subject animal.

A figure describing the background sound intensity outside of the anechoic chamber and inside the chamber without stimuli is included in Appendix 1. These measurements include the background sound intensities both outside and inside of the chamber in a laboratory at Purdue University (Fig. A1, Appendix 1) and background sound intensities inside of the chamber where the experiments were conducted at the Wildlife Center of Virginia (Fig. A2, Appendix 1). Note that the background intensity levels over the range of frequencies used in this study (1000–6000 Hz) were uniformly below 10 dB, suggesting that the chamber functioned as designed.

#### Stimulus/recording equipment

Experimental stimuli and response recordings were controlled by an RZ6 Multi I/O Processor unit (Tucker-Davis Technologies Inc., Alachua, FL, USA). Output from the RZ6 was passed through an Ultragraph Pro FBQ6200HD equalizer (Behringer, Willich, Germany) and then a Crown D-75 amplifier (Crown Intl., Elkhart, IN, USA) before sounds was played from a JBL Control 25AV speaker (JBL Professional, Los Angeles, CA, USA). AEPs were measured using a RA4LI headstage with RA4PA 4-channel Medusa Preamp (Tucker-Davis Technologies Inc., Alachua, FL, USA) connected to the RZ6. We used 3-lead, 27-gauge, 13-mm Disposable Horizon Subdermal Needle electrodes (Rochester Electro-Medical, Lutz, FL, USA) placed posterior to the left ear opening (+), on the crown of the head (−) and on the breast (ground). The electrodes were adjusted so that impedances were under 3 kΩ. The headstage was placed next to the animal inside the Faraday cage. The speaker was positioned 45 cm above the bird’s head and outside of the Faraday cage. The rest of the equipment remained outside the anechoic chamber.

#### Program parameters

A computer running BioSigRZ (Tucker-Davis Technologies Inc., Alachua, FL, USA) controlled both stimulus presentation and electrophysiology recording simultaneously. Stimuli were generated using SigGenRZ (Tucker-Davis Technologies Inc., Alachua, FL, USA).

#### Speaker calibration

The JBL speaker inside the anechoic chamber was calibrated in two steps. First, we loaded the CAL200K file in BioSigRZ and used the calibration tool with a PCB Model 378C01 microphone (sensitivity, 2.0 mV/Pa). We used the calibration software within BioSigRZ to generate a calibration file. Next, we replaced the PCB microphone with a Type 2250 Hand Held Sound Level Meter with the microphone on a 2.0-m cable (Brüel & Kjaer, Naerum, Denmark). We used this sound level meter (Z-weighting scale) to verify calibration and, if necessary, to adjust the equalizer so that all frequencies from 100 to 8000 Hz were calibrated to within 1 dB of 80 dB Sound Pressure Level (SPL).

### Stimulus sounds

Each AEP for all stimuli was generated as the average of 500 repeated stimulus presentations (see Gall *et al.*, 2011). We evaluated responses of eagles to a suite of different candidate sounds. Each type of stimulus sound was grouped such that all tones were played together, followed by all AM sounds, etc. Stimuli themselves were presented sequentially. Between each stimulus type, we recorded the ABR to a broad-band click (see below) to provide a baseline measure reflecting the state of the anaesthetized animal. Sessions would end if the difference in the response to a click was >20% of the response to the initial click (see Gall and Lucas, 2010); however, none of the eagles surpassed this threshold suggesting that the state of anaesthesia was stable throughout each trial.

The stimulus types were determined ahead of time to ensure the experiments were grouped into 30-min blocks of stimuli. All stimuli except clicks had 2 ms cos^2^ onset and offset ramps (e.g. Gall and Lucas, 2010). The stimulus types were as follows (see Appendix 2 for an overview of the stimuli):

#### Background noise

We used two generic forms of noise for this study (white and pink noise) as well as a no-noise treatment. White noise contained an equal amount of energy at all frequencies from 0 to 12 kHz. The energy profile of pink noise is skewed towards lower frequencies (technically inversely proportional to frequency) and was also low-pass filtered at 12 kHz. White noise approximates a number of natural sources of noise (Handel and Chung, 1993). Noise generated by wind through vegetation, for example, can have properties similar to white noise (Bolin, 2009). However, noise profiles under many conditions tend to have more energy at lower frequencies. Pink noise is commonly used in a variety of studies to mimic noisy backgrounds (Howarth and Griffin, 1991; Airo *et al.*, 1996; Schlittmeier and Hellbrück, 2009; Potvin *et al.*, 2016). Moreover, the over-representation of lower frequencies in pink noise relative to white noise has been shown in measurements of wind turbine noise (Schomer *et al.*, 2015). Note that these noise backgrounds are not meant to mimic a specific noise, but were used as a general representation of two kinds of noise profiles that are commonly found in nature.

#### Tones

We tested six different pure tones (0.5, 1, 2, 3, 4, 5 kHz). This is a standard tone range shown to be processed well in many bird species (Dooling, 1982), including bald eagles and red-tailed hawks (McGee *et al.*, 2019). While some songbirds show reasonably good processing of tones higher than 5 kHz (e.g. Vélez *et al.*, 2015), we limited our tests to a maximum of 5 kHz because evidence suggested that eagles were not particularly good at processing tones above 5 kHz (McGee *et al.*, 2019), and adding additional tones above 5 kHz would impact our ability to test the additional sounds in our study. We randomized the order of the six tones for each eagle, but all treatments for an individual eagle received the same six-tone order. Tones were 30 ms in duration, preceded by 10 ms background only and played at a rate of 18.3 Hz with silence between each 40 ms stimulus (i.e. 10 ms noise then 30 ms tone+noise treatment). The treatments were as follows: each tone was presented at 80 dB SPL initially with a silent background. The same order of tones was then presented at 80 dB SPL with 80 dB SPL white background noise. Finally, the tones were presented at 80 dB SPL with 80 dB SPL pink background noise. This experiment was then repeated first with 70 dB SPL tones and then 60 dB SPL tones, but noise level remained 80 dB SPL. The order of background noise conditions was not varied during the experiments, partly to initially ensure that we would have data from multiple individuals for comparison even if there were issues with anaesthesia. After we performed the experiments with several bald eagles without issue, we analysed auditory responses and found no suggestion of a change in auditory performance over time, so we continued with the same order for our noise presentation. The consistency of noise masking across different individuals and stimulus sounds suggests that noise masking had a large effect relative to any potential adaptation of the auditory system to the stimuli.

#### Harmonic stacks

Many avian vocalizations contain complex harmonic patterns (see Nelson and Marler, 1990) and species can vary widely in how they process these sounds (Lucas *et al.*, 2015). Moreover, the perception of sounds can be affected by the level of inharmonicity of the sound. For example, humans perceive inharmonic sounds as more urgently warning sounds compared with harmonic sounds (Hellier *et al.*, 1993). We therefore tested eagles’ responses to both harmonic stacks and non-harmonic stacks.

We tested three different stacks of tones at 80 dB SPL, first with a silent background, then with white noise and finally with pink noise. The first stack was a 1-kHz harmonic stack containing 1, 2, 3, 4 and 5 kHz (i.e. a chord composed of the majority of tones tested individually). The second stack was composed of four tones (1.2, 1.8, 2.4 and 3.0 kHz) with a spacing of 600 Hz between the tones. The lowest tone in this series (0.6 Hz) is missing but the other four tones will nonetheless generate a strong amplitude modulation at 0.6 Hz (see Henry *et al.*, 2016). This stack was chosen because a 600-Hz amplitude modulation has previously been shown to be highly stimulating to a number of avian species (Henry *et al.*, 2016).

The final stack was a series of non-harmonic tones (1.0, 2.2, 3.3, 3.6 and 4.7 kHz), which fall in the same range as the harmonic stack, but the component tones were arbitrarily chosen with non-uniform spacing between tones. Stack stimuli were 40 ms in duration, starting with 10 ms background, then 30 ms of the stack stimulus played over the background. These sounds were presented at 18.3 Hz.

#### Amplitude modulation

Amplitude modulation is a fundamental property of many vocal signals (Nelson and Marler, 1990; Zeng *et al.*, 2005), and the auditory processing of amplitude modulation has been shown to match these properties (Lucas *et al.*, 2015; Cai and Dent, 2020) making AM stimuli a good candidate for this study. AM signals were generated by playing three equally spaced tones. The middle tone is called the carrier and the other two tones are sidebands. The high sideband is the carrier plus the desired AM rate and the low sideband is the carrier minus the AM rate. We used three different carrier frequencies (1, 2 and 3 kHz) and three different AM rates (100, 400 700 Hz) with each carrier. The carrier tones were chosen based on tones where bald eagles were shown to have maximum sensitivity (McGee *et al.*, 2019), and the range of AM rates tested mirrors the sensitivity range previously measured in songbirds (Henry and Lucas, 2008). Each of the nine carrier-AM stimuli was presented first with a silent background, then with white noise and finally pink noise. AM treatments were 60 ms in duration, with 10 ms background noise preceding the 50 ms AM signal+noise. These stimuli were played at a rate of 13.1 Hz. Note that this phase was not alternated following our previous studies (Henry and Lucas, 2008; Gall *et al.*, 2012).

#### Frequency sweeps

Frequency sweep stimuli were rapid linear frequency changes from 1 to 6 kHz (up sweep) or 6 to 1 kHz (down sweep). The rationale for using FM sounds is that they are likely to be detectable against a noise background (see Discussion). Moreover, birds have been shown to be more capable than mammals at resolving rapidly FM sounds (Dooling *et al.*, 2002), making frequency modulation potentially important alerting stimuli. We tested two different rates of frequency sweeps: either fast (30 ms duration) or slow (50 ms duration). Each sweep was preceded by 10 ms of background noise such that fast sweep treatments were 40 ms total (10 noise plus 30 ms signal+noise) and slow sweeps were 60 ms (10 ms noise plus 50 ms signal+noise) in duration. Fast sweeps were presented at a rate of 18.3 Hz and slow sweeps at 13.1 Hz.

#### Sinusoidal frequency modulation

We tested eagles with sinusoidal FM sounds in addition to the linearly FM sounds. This provides data on a broader range of FM stimuli. As discussed above, rapid frequency modulation is particularly relevant for birds (Dooling *et al.*, 2002), and may be useful alerting stimuli in noisy conditions (see Discussion). Sinusoidal FM stimuli were all centred on 2 kHz with frequency modulation sinusoidally at two modulation rates (70 and 110 Hz) and two depths (400 and 700 Hz; here defined as the difference between mean and minimum or maximum frequency). Treatments were 85 ms in duration with 10 ms background noise followed by 75 ms stimulus+noise. These stimuli were presented at 10.1 Hz.

#### Clicks

We used short, 0.1 ms, 80 dB broadband click averaged from 400 stimulus presentations to determine a baseline responsiveness of the subject eagle auditory system during the experiments. Clicks were alternated in phase by 180^°^ to minimize any cochlear microphonic components that may bias our measure of the ABR elicited by the click (Hall, 2007). Clicks were broadcast before and after each experiment and before and after each top-up anaesthesia injection.

### AEP analysis

Our stimuli fall into two categories with properties that require different types of analyses (see Appendix 3 for example AEPs in response to selected stimuli). One category is composed of one or a series of static tones. Treatments in this category include the pure tones, harmonic and inharmonic stacks and the AM stimuli. The second category is composed of more dynamic FM tones. Stimuli in this category include the linear frequency sweeps and the sinusoidal FM tones. The auditory system will phase-lock to all of these tones and AM components. Phase-locking results from populations of neurons in the auditory system firing at approximately the frequency of each tone or at the frequency of the amplitude modulation (Viemeister and Plack, 1993).

**Figure 1:**
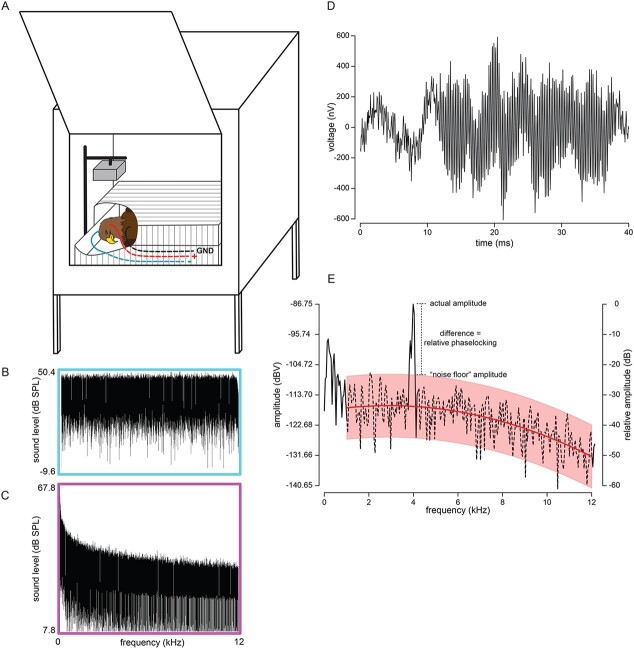
AEPs were measured in bald and golden eagles for a variety of different stimulus sounds. The anaesthetized eagles were placed in a Faraday cage inside an anechoic chamber (**A**). AEPs to a variety of stimuli were measured in quiet, and subsequently under conditions of a background of white noise (**B**, equal intensity across broad range of frequencies) and a background of pink noise (**C**, higher intensity at lower frequencies). Each sound was presented 500 times, and the response of the auditory system was averaged to produce the AEP (**D**, response to 4 kHz single tone). We then analysed the FFT AEP (black line) and measured the response to the stimulus relative to the background (or noise floor) response (**E**). A second-order polynomial line (red line) was fit to the FFT data (dashed line) excluding values in the vicinity of the stimulus frequency and excluding frequencies <1 kHz. The residuals from this polynomial were sorted and used to define a 95% confidence interval band that served as a ‘noise floor’. The actual response to the stimulus tone (or tones) was compared against the expected noise floor at the same frequency to determine relative phase-locking; an index of how well the peripheral auditory system encodes the stimulus sound. The example in (E) is the analysis of the AEP shown in (D).

Phase-locking to tones at the level of the brainstem is called the Frequency Following Response (FFR; Hall, 2007). The amplitude or strength of the FFR is a measure of the firing synchrony of brainstem neurons and the number of neurons responding to the stimulus. Thus, phase-locking strength is an excellent index of how well the auditory system processes many types of sounds (see Kraus and Nicol, 2005). Auditory processing of static tones or static AM components can be characterized with a spectrum that integrates phase-locking over the duration of the stimulus. FFR amplitude was measured using PRAAT software (Boersma, 2001) by first calculating the frequency spectrum of the AEP with a Fast Fourier Transform (FFT; sampling rate, 40 kHz; FFT size, 2048 points; frequency resolution, 19.5 Hz). We then calculated the maximum amplitude (in dBV) of peaks +/−50 Hz from the stimulus frequency. Our estimate of phase-locking to any of the stimulus tones was calculated as the amplitude of phase-locking at the stimulus frequency minus the 95th percentile of the noise floor magnitude at the stimulus frequency (see Fig. 1 and below). Note that the noise floor itself was not significantly different between species or age groups (see Appendix 4).

The auditory system also phase-locks to strong AM components and to the amplitude envelope of the harmonic stack with the missing fundamental. Phase-locking to the AM component of a stimulus is called the Envelope Following Response (EFR), which is measured with the same FFT method described for the FFR.

Random neural activity in the brain generates a noise floor of the AEP that is independent of the neural processing of the stimulus. The noise floor of the AEP will change with the type of masking noise (none, pink or white) broadcast with the stimulus. This change in AEP noise floor makes it difficult to distinguish the strength of phase-locking to our stimuli from the overall level of the AEP noise floor. For that reason, we estimated the upper 95% confidence limit of the AEP noise floor at the frequency of each stimulus tone or AM rate, and we subtracted that number from the absolute amplitude of the AEP spectrum at the frequency of the stimulus or AM rate. This was done as follows: the AEP noise floor was first generated for each individual AEP recording using an FFT of the AEP data for frequencies 500–5000 Hz, but excluding stimulus-related peaks and shoulders for all frequencies in the stimulus within +/−100 Hz of those stimulus frequencies. The approximate average AEP noise-floor amplitude at each stimulus frequency or AM rate was estimated with a second order polynomial fit to the trimmed spectrum using Proc MIXED in SAS v9.4. Higher-order polynomial equations did not provide a better fit than the second-order polynomial. A separate model was generated for each bird, stimulus type, noise type and tone frequency (for pure tones). We also deleted outliers from the noise floor that had model residuals that were ≤15 dBnV or >15 dBnV. The model was rerun if outliers were identified. For example, the noise floor for a trial with a 3 kHz pure tone would be calculated from the data for 0.5–2.9 and 3.1–5.0 kHz. We then estimated the 95% confidence limit of the residuals, added that to the expected amplitude of the noise floor at the stimulus frequency based on the fitted polynomial curve and subtracted this sum from the amplitude of the peak of the spectrum +/− 50 Hz around the stimulus frequency. This value describes how much stronger the auditory response of the subject eagle was to the stimulus compared with the noise floor (Fig. 1E).

For frequency sweeps and sinusoidal FM stimuli, we used an auto-correlation method implemented in the Pitch (ac) analysis function in Praat (v6.1.05; Boersma, 2001) to generate an estimate of phase-locking frequency as a function of time. The details of the pitch analysis for the frequency sweeps were as follows: time step, 0.00025 s; pitch floor, 1000 Hz; very accurate, ‘yes’; silence threshold, 0.03; voicing threshold, 0.45; octave cost, 0.01 per octave; octave-jump cost, 0.35; voiced/unvoiced cost, 0.14; and pitch ceiling, 6000 Hz. The details of the pitch analysis for sinusoidal FM tones were identical to analysis of sweeps except the following: time step, 0.000125 s; pitch floor, 2000 Hz – modulation depth; and pitch ceiling, 2000 Hz + modulation depth. The autocorrelation pitch analysis also provides an index of the relative strength of the AEP waveform, which indicates the degree of periodicity of the candidate tone (here phase-locking to the stimulus tone) relative to the maximum possible autocorrelation, ranging from 0 (no periodicity) to 1 (maximal periodicity). We evaluated the auditory response to FM stimuli using the difference between phase-locking frequency and the stimulus frequency as a function of time. Phase-locking strength was also analysed as a function of time. Note that we folded all cycles of the sinusoidal FM AEP starting at sin(0) through sin(360) and thereby analysed a single average cycle for each sinusoidal FM stimulus. However, the first 0.015 s were trimmed off of the AEP to eliminate any onset responses, and data were only included in the analysis if relative phase-locking strength was greater than 0.

### Statistics

We used repeated measures linear mixed models (Proc Mixed in SAS v9.4) to test for species and treatment effects on auditory responses to our test stimuli. The SAS code used for our analyses is listed in Appendix 5. The dependent variable in our models was relative phase-locking strength (in dBV) for the fixed frequency stimuli (i.e. tones, AM, harmonic stacks and inharmonic stack). Higher phase-locking values indicate better processing of the stimulus sound. A decrease in phase-locking strength during treatments with noise therefore means that noise negatively affected stimulus sound processing. Response variables included the stimulus frequencies (either as a tone frequency or AM frequency), time (for FM stimuli), dB level (for tones), noise background treatment, relative age of the subject eagle (juvenile/adult) and eagle species. Individual eagles were treated as the subjects in the repeated measures analysis. All three-way interaction terms were initially included and trimmed in order of decreasing *F*-value when found to be non-significant (*P* > 0.05). Two-way interaction terms were then addressed in a similar manner. Estimates presented are least squares means and standard errors calculated from the final statistical model for each stimulus (Proc Mixed, LSMEANS). Normality of residuals and homogeneity of variances were confirmed using PROC UNIVARIATE (SAS).

Noise masking was described using a percentage of the phase-locking response without noise. In the dBV scale, baseline activity was −86.75 dBV. Noise masking percentages are therefore calculated using the ratio of phase-locking with noise to phase-locking without. For example, a tone may have phase-locking of −69.80 dBV without noise and −82.22 dBV in pink noise. In that case, noise masking would be ~73% of the non-masked treatment.

\begin{align*} Noise\ masking\ sample\ calculation:\notag\\100\%\ast \left(1-\tfrac{-82.22-\left(-86.75\right)}{-69.80-\left(-86.75\right)}\right) \end{align*}

Note that these effect sizes are measured relative to dBV, not to sound intensity, which scales to 10^dBV^. Nonetheless, this use of effect size is justified given that the perception of sound scales more closely to the log of intensity instead of linearly to intensity (Møller, 2006).

For stimuli with either linear or sinusoidal FM we measured two different dependent variables: phase-locking strength and the frequency difference between the AEP and stimulus. Each measure was analysed separately.

## Results

### Tones

Phase-locking to single tones in both species of eagles was lower when the tones were played at lower amplitude (60 dB SPL = −81.55 ± 0.42 dBV, 70 dB SPL = −76.84 ± 0.57, 80 dB SPL = −73.18 ± 0.42; F_2,10_ = 154.80, *P* < 0.0001). Similarly, phase-locking to tones was lower in noisy backgrounds than in silence (no noise, −69.80 ± 0.40 dBV; pink noise, −82.22 ± 0.40) and slightly lower in pink noise than in white noise (white noise, −79.56 ± 0.40 dBV; noise effect: F_2,8_ = 610.92, *P* < 0.0001). The degradation of the tone stimuli by background noise (73% for pink noise; 58% for white noise) suggests that tones may not be strong candidates for implementation as an eagle alerting stimulus in wind farms.

Overall, bald and golden eagles did not differ in their phase-locking to tones (species main effect F_1,4_ = 6.6, *P* = 0.062). However, bald eagles had stronger phase-locking to the quieter tones (60 dB SPL) compared with golden eagles (bald eagle = −73.63 ± 0.55 dBV, golden eagle = −76.59 ± 0.93; F_2,10_ = 2.50, *P* = 0.032), but only when there was no background noise (species × tone dB × noise-type interaction: F_4,20_ = 3.26, *P* = 0.033). With background noise, bald eagle adults (white noise, −78.67 ± 0.44 dBV; pink noise, −80.92 ± 0.44) also performed better than golden eagle adults (white noise, −82.38 ± 1.14 dBV; pink noise, −84.58 ± 1.13), with no difference among the juveniles of the two species (bald: white noise, −78.40 ± 0.66; pink noise, − 87.19 ± 0.66; golden: white noise, −78.36 ± 0.94; pink noise, −82.47 ± 0.93; species × noise × age interaction: F_2,8_ = 14.82, *P* = 0.0020). Finally, noise masked the stimulus tones more for 60 than 80 dB SPL tones, with different noise masking patterns for different tone frequencies (tone × tone amplitude × noise interaction: F_20,120_ = 2.33, *P* = 0.0025).

### Tone stacks

#### Harmonic stack

Overall phase-locking to the different component tones of the 1–5 kHz harmonic stack matched the expectation that the auditory system responds strongly to 2–3 kHz tones (1 kHz: −80.04 ± 2.15; 2 kHz: −73.59 ± 1.99; 3 kHz: −75.75 ± 1.93; 4 kHz: −80.76 ± 1.99; 5 kHz: −79.96 ± 2.15; all in dBV; main effect of tone frequency: F_4,28_ = 11.45, *P* < 0.0001).

Background noise had a strong effect on the auditory response to the harmonic stack, masking the no-noise response (−74.70 ± 1.69 dBV) by 37% in white (−79.16 ± 1.69 dBV) and 53% in pink (−81.12 ± 1.68 dBV) noise (main noise effect: F_2,10_ = 68.75, *P* < 0.0001). Adult bald eagles generally performed better in noise than juveniles (age × noise: F_2,10_ = 11.43, *P* = 0.0026), though this difference was not seen in the two golden eagle subjects (one adult, one juvenile). Without noise, golden eagles (−72.91 ± 2.87 dBV) had stronger phase-locking than bald eagles (−76.50 ± 1.76 dBV), and both species were similar in white (bald: −78.94 ± 1.76 dBV; golden: −79.39 ± 2.87) and pink (bald: −80.74 ± 1.76 dBV; golden: −81.51 ± 2.87) noise (species × noise effect: F_2,10_ = 14.56, *P* = 0.0011). There was no significant main effect of species on phase-locking strength (F_1,5_ = 0.00, *P* = 0.96).

#### Mistuned stack

The mistuned stack (unequally spaced tones) was processed similarly by both eagle species (F_1,5_ = 5.82, *P* = 0.061), and again significantly masked by noise (no-noise: −84.83 ± 0.20 dBV; white: −85.36 ± 0.20; pink: −85.72 ± 0.20; F_2,14_ = 5.60, *P* = 0.016). A significant interaction between tone frequency and subject age (F_4,24_ = 3.56, *P* = 0.021) showed that at intermediate tone frequencies the juvenile eagles had weaker phase-locking than the adults (2.2 kHz: juvenile: −86.13 ± 0.35 dBV, adult: −84.42 ± 0.28; 3.3 kHz: juvenile: −86.73 ± 0.35, adult: −85.22 ± 0.28; 3.6 kHz: juvenile: −85.71 ± 0.35, adult: −84.64 ± 0.28). The same was not true for the lowest (1.0 kHz: juvenile: −85.35 ± 0.35 dBV, adult: −85.34 ± 0.28) and highest frequency components (4.7 kHz: juvenile: −84.75 ± 0.35 dBV, adult: −84.76 ± 0.28).

#### Missing fundamental (600 Hz) stack

Eagles had a highly variable response to the different components of the missing fundamental (600 Hz) stack. There was no significant main effect of species on phase-locking to the components of the missing fundamental stack (F_1,5_ = 0.16, *P* = 0.71). However, there was a significant species × component interaction (F_4,24_ = 12.8, *P* < 0.001) caused by bald eagles exhibiting strong phase-locking to the 0.6 kHz AM (−74.88 ± 1.04 dBV) and weaker phase-locking to the 1.2 kHz tone (−79.73 ± 1.04 dBV). In contrast, golden eagles showed weak processing of the 0.6 kHz AM (−83.13 ± 1.77 dBV) but exhibited strong phase-locking to the 1.2 kHz tone (−73.04 ± 1.77 dBV). As with pure tones, the main effect of noise had a significant effect on phase-locking of this stack stimulus (no noise, −77.28 ± 0.85 dBV; white noise, −79.18 ± 0.85; pink noise, −80.16 ± 0.85; F_2,14_ = 7.32, *P* = 0.0067) with 20% masking in white noise and 30% masking in pink noise.

### AM stimuli

The AM stimulus was created by playing three equally spaced tones: a carrier, a low sideband (carrier minus *F*) and a high sideband (carrier plus *F*), resulting in an AM rate equal to the spacing (*F*). We separately analysed the eagles’ ability to phase-lock to each of the three tones and to the AM component for a range of carriers (1, 2 and 3 kHz) and AM rates (100, 400, 700 Hz) in each of the noise backgrounds. Results for processing of amplitude modulation and the carrier are described here. The results from the sidebands, which largely mirror results presented for amplitude modulation and the carrier, are presented in supplementary Appendix 6.

#### AM rate

We found significant main effects of AM rate (F_2,12_ = 47.64, *P* < 0.0001), carrier frequency (F_2,12_ = 21.39, *P* = 0.0001) and noise background (F_2,12_ = 99.41, *P* < 0.0001) on phase-locking amplitude to the AM component. Generally, the strongest phase-locking to the AM envelope was measured for the 2 kHz carrier (1000 Hz: −83.41 ± 0.69 dBV; 2000 Hz: −77.91 ± 0.69; 3000 Hz: −79.27 ± 0.69) with a 400 Hz AM rate (100 Hz: −76.71 ± 0.97 dBV; 400 Hz: −74.07 ± 0.94; 700 Hz: −83.03 ± 0.97). This stimulus was also most resistant to noise of all of the AM stimuli.

Bald eagles (−78.86 ± 0.49 dBV) were generally better at phase-locking to the AM component compared with golden eagles (−81.54 ± 0.80 dBV; F_1,5_ = 8.39, *P* = 0.034); however, this difference changed with AM rate (bald eagle: 100 Hz = −76.81 ± 0.71, 400 Hz = −75.10 ± 0.71, 700 Hz = −84.68 ± 0.71; golden eagle: 100 Hz = −81.64 ± 1.16, 400 Hz = −79.29 ± 1.16, 700 Hz = −84.61 ± 1.16; species × AM rate interaction: F_2,12_ = 6.47, *P* = 0.012). In addition to the species differences, there was a significant interaction between the carrier frequency, background noise and age on the magnitude of phase-locking to the amplitude modulation (F_4,24_ = 3.32, *P* = 0.027). The strongest effect was in noisy conditions where adults (no noise, −75.09 ± 0.94 dBV; white, −16%, −76.95 ± 0.97; pink, −28%, −78.30 ± 0.94) exhibited less of a decrease in phase-locking of the amplitude modulation in stimuli with 0.2 kHz carriers than juveniles (no-noise, −76.82 ± 1.16; white, −32%, −80.04 ± 1.19; pink, −35%, −80.30 ± 1.17).

#### AM carrier

Phase-locking amplitude to the carrier tones would be expected to vary with differences in carrier frequency and noise background, and we find support for both factors (carrier: F_2,10_ = 14.22, *P* = 0.0012; noise: F_2,12_ = 54.86, *P* < 0.0001) as well as for the interaction between the two (F_4,24_ = 10.11, *P* < 0.0001) and carrier frequency × noise × subject age (F_4,24_ = 3.20, *P* = 0.031). Generally, this means that eagles can hear the 2 kHz carrier stimuli better (−77.56 ± 0.90 dBV) than the 1 kHz (−83.37 ± 0.92 dBV) but about the same as the 3 kHz carrier stimuli (−78.01 ± 0.63 dBV). Similarly, the reduction in phase-locking strength in background noise relative to no noise was less pronounced for 2 kHz (white: juvenile = −11%, adult = −12%; pink: juvenile = −18%, adult = −23%) than for 1 kHz (white: juvenile = −24%, adult = −50%; pink: juvenile = −55%, adult = −36%) and 3 kHz carriers (white: juvenile = −51%, adult = −42%; pink: juvenile = −63%, adult = −39%). Juveniles and adults differed in the level of phase-locking to 1 kHz (juvenile, −82.12 ± 1.43 dBV; adult, −82.03 ± 1.21), 2 kHz (juvenile, −78.46 ± 1.37 dBV; adult, −74.43 ± 1.18) and 3 kHz (juvenile, −71.86 ± 1.39 dBV; adult, −75.46 ± 1.19) carriers. We did not find a main-effect difference between the two species on phase-locking to that carrier tone (F_1,5_ = 0.01, *P* = 0.95), which suggests that overall the 2 kHz carrier is a good candidate for a stimulating sound. It stimulates both bald and golden eagles and phase-locking remains strong in noise and for different age classes.

### Linear FM (sweeps)

#### Phase-locking strength during the FM sweep

**Figure 2:**
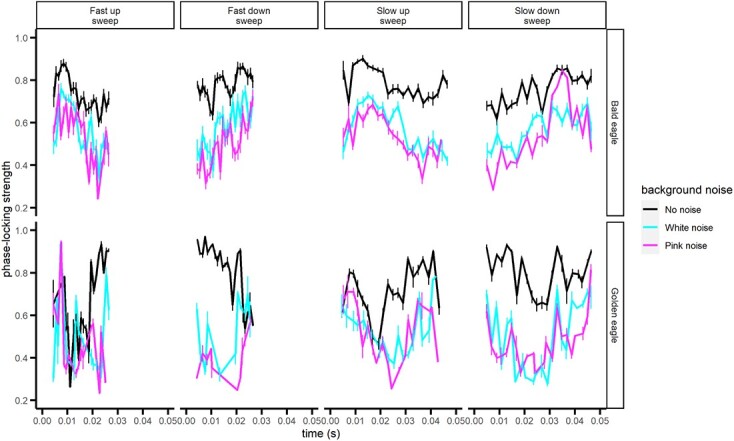
Strength of phase-locking to linear sweeps under three noise profiles by bald (top figures) and golden (bottom figures) eagles. The data are means ± SE. See Methods for descriptions of the stimuli.

Phase-locking strength (Fig. 2) was generally higher without noise than in white or pink noise (fast up: F_2,12_ = 3.13, *P* = 0.081; fast down: F_2,10_ = 19.51, *P* = 0.0004; slow up: F_2,14_ = 68.56, *P* < 0.0001; slow down: F_2,14_ = 6.21, *P* = 0.012). Comparing the two species, bald eagles generally exhibited stronger phase-locking than golden eagles to all but the fast/down sweep (fast up: F_1,4_ = 38.43, *P* = 0.0034; fast down: F_1,3_ = 1.63, *P* = 0.292; slow up: F_1,5_ = 19.55, *P* < 0.0069; slow down: F_1,4_ = 7.92, *P* = 0.048). Significant time × species interactions (fast up: F_1,1422_ = 28.83, *P* < 0.0001; fast down: F_1,1304_ = 8.21, *P* = 0.0042; slow up: F_1,2264_ = 10.68, *P* = 0.0011; slow down: F_1,3682_ = 11.09, *P* = 0.0009) show that golden eagles have weaker phase-locking in response to the lower portions of the frequency sweep stimuli and that both species are similar at the higher frequency portions (Fig. 2).

**Figure 3:**
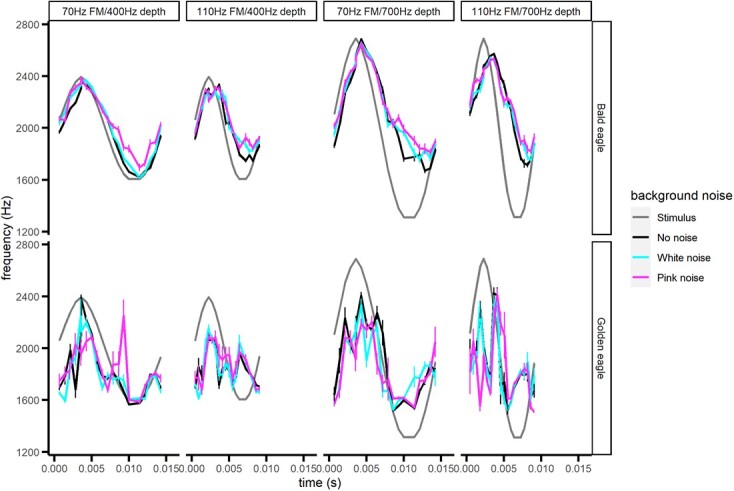
Estimated frequency of phase-locking to sinusoidal FM tones under three noise profiles by bald (top figures) and golden (bottom figures) eagles. The data are averaged across a single cycle and are shown as means ± SE. See Methods for descriptions of the stimuli (here shown in grey).

### Sinusoidal FM

#### Frequency difference

Eagle ability to follow the frequency of the sinusoidal FM stimuli was not significantly affected by background noise (Fig. 3; 70 Hz FM/400 Hz depth: F_2,14_ = 1.92, *P* = 0.184; 70 Hz FM/700 Hz depth: F_2,14_ = 0.67, *P* = 0.527; 110 Hz FM/400 Hz depth: F_2,14_ = 0.55, *P* = 0.589; 110 Hz FM/700 Hz depth: F_2,14_ = 0.68, *P* = 0.521). The frequency difference varied with time (Fig. 3) for all stimuli tested (70/400 Hz: F_19,114_ = 9.88, *P* < 0.0001; 70/700 Hz: F_19,114_ = 5.72, *P* < 0.0001; 110/400 Hz: F_19,113_ = 13.6, *P* < 0.0001; 110/700 Hz: F_19,114_ = 8.42, *P* < 0.0001) as well as species (70/400 Hz: F_1,6_ = 30.75, *P* = 0.0015; 70/700 Hz: F_1,6_ = 20.55, *P* = 0.0040; 110/400 Hz: F_1,6_ = 56.67, *P* = 0.0003; 110/700 Hz: F_1,6_ = 37.95, *P* = 0.0008) and the interaction of species × time (70/400 Hz: F_19,114_ = 2.03, *P* = 0.0120; 70/700 Hz: F_19,114_ = 2.45, *P* = 0.0019; 110/400 Hz: F_19,113_ = 2.08, *P* = 0.0097; 110/700 Hz: F_19,114_ = 4.16, *P* < 0.0001). In general, phase-locking to the stimulus frequency in golden eagles was consistently too low during the higher-frequency portions of the sinusoidal frequency modulation, whereas the bald eagle auditory system followed the stimulus sound more closely (Fig. 3). The frequency of phase-locking for both eagle species was higher than the stimulus frequency during low-frequency portions of the stimulus (Fig. 3), suggesting that there is both a lag in auditory response to changes in stimulus frequency and potentially a mismatch between stimulus frequency and phase-locking when the stimulus is changing rapidly.

#### Phase-locking strength results

**Figure 4:**
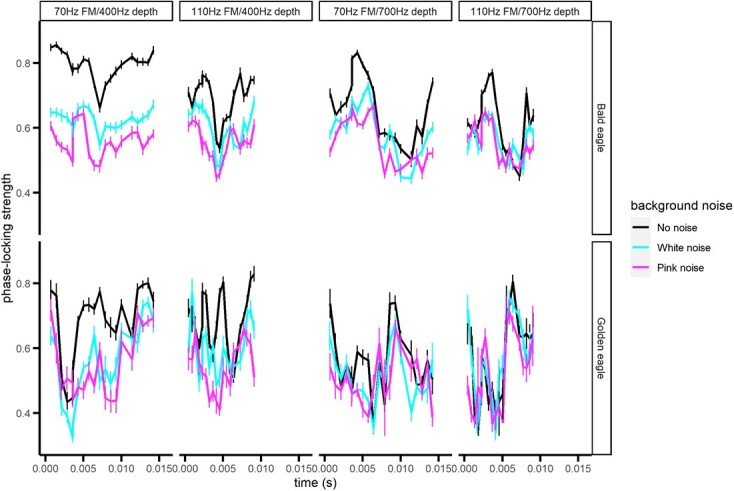
Strength of phase-locking to sinusoidal FM tones by bald and golden eagles. The data are averaged across a single cycle and are shown as means ± SE. See Methods for descriptions of the stimuli.

Phase-locking strength for sinusoidal FM stimuli decreased significantly for all but the fastest modulating stimulus in the presence of background noise (Fig. 4; 70/400 Hz: F_2,14_ = 27.27, *P* < 0.0001; 70/700 Hz: F_2,14_ = 10.99, *P* = 0.0013; 110/400 Hz: F_2,14_ = 11.41, *P* = 0.0011; 110/700Hz: F_2,14_ = 2.90, *P* = 0.0882). Phase-locking strength also varied significantly with time during the cycle for all but the fast/shallow stimulus (70/400 Hz: F_19,114_ = 2.45, *P* = 0.0019; 70/700 Hz: F_19,114_ = 2.84, *P* = 0.0003; 110/400 Hz: F_19,113_ = 1.55, *P* = 0.0823; 110/700 Hz: F_19,114_ = 2.97, *P* = 0.0002). There was generally no main effect of species on phase-locking strength (70/400 Hz: F_1,6_ = 3.35, *P* = 0.117; 70/700Hz: F_1,6_ = 0.00, *P* = 0.946; 110/400 Hz: F_1,6_ = 8.44, *P* = 0.0271; 110/700 Hz: F_1,6_ = 0.26, *P* = 0.628) but all stimulus combinations had significant interactions between time and species (70/400 Hz: F_19,114_ = 1.86, *P* = 0.0236; 70/700 Hz: F_19,114_ = 2.07, *P* = 0.0098; 110/400 Hz: F_19,113_ = 2.84, *P* = 0.0003; 110/700 Hz: F_19,114_ = 4.67, *P* < 0.0001). In general, bald eagles had much higher phase-locking strength for high frequencies than golden eagles, and the species showed similar phase-locking strengths at lower frequencies (Fig. 4).

## Discussion

**Figure 5:**
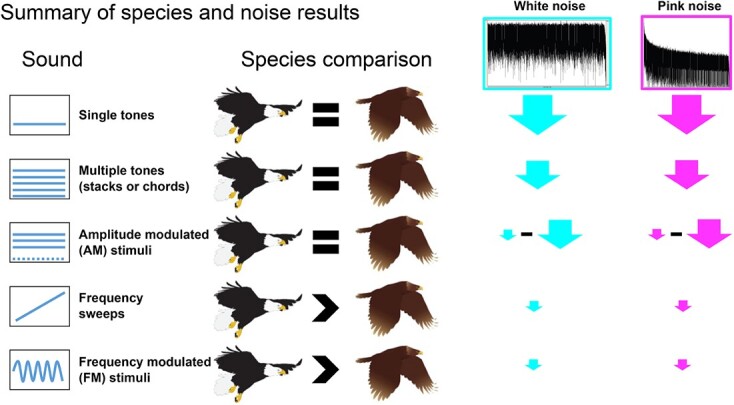
Illustrative overview of auditory responses by both eagle species in response to five categories of sound stimuli and two noise types. Symbols for species comparison are approximate representations of how bald eagles (left) performed in the auditory metrics we analysed, compared with golden eagles (right). The performance of bald eagles was generally about the same (=) or better than (>) that of golden eagles for the stimulus sounds we tested. For white noise and pink noise, the size of the arrows gives a relative overall indication of how much noise decreased auditory performance of both eagle species. Noise masking effect sizes ranged from little-to-no effect of noise (small arrow) to a near complete masking of the auditory response to tones (large arrow), with intermediate masking of tone stacks and some AM stimulus combinations.

Both species of eagle responded similarly to static sounds (tones, tone stacks and three-tone AM chords). In contrast, bald eagles were better than golden eagles at processing rapidly FM sounds (linear sweeps and sinusoidal FM tones) and relatively rapid amplitude modulation (see Fig. 5). Noise most strongly affected auditory processing of static sounds, whereas dynamic sounds were relatively resistant to noise masking and therefore are good candidates for implementation in the field, as long as they are slow enough for golden eagles to process well. Specifically, an FM rate of over 150 Hz/ms appears to be too rapid for golden eagles. This is true for both linearly FM and sinusoidal FM sounds. In contrast, FM rates of 50 Hz/ms or less seem to be efficiently processed by both species.

Our results suggest that adult eagles process sounds in noise better than juveniles. Examples include phase-locking to elements of the harmonic and mis-tuned stacks and processing of AM envelopes. However, the sample sizes for these tests are small so the results should be taken with some caution. More generally, we were able to test only two golden eagles for this study. The fact that golden eagles and bald eagles showed similar processing of static tones, but different processing of more dynamic AM and FM sounds, suggests that our results are a true reflection of species differences (see below for additional discussion of the potential basis of these patterns). Nonetheless, our results if anything call for additional studies of these species.

AEPs provide important information about the processing of sound stimuli at the level of the peripheral auditory system (Hall, 2007). Evidence from human auditory processing shows that the brainstem-derived FFR (i.e. pre-attentive processing) correlates with the processing of speech signals (Krishnan, 2002) and its components (Russo *et al.*, 2004; Krishnan and Gandour, 2009). Additionally, brainstem FFRs encode both the general properties of the sound source and higher-order information about sound modulation and filtering that can be compared with cortical ‘what’ and ‘where’ streams described for visual processing (Kraus and Nicol, 2005). Therefore, the amount and type of information encoded at the brainstem level for any given auditory stimulation can be used to assess how well complex sounds are encoded. This is particularly important here given the differences between species in their response to dynamic sounds. Additionally, comparing peripheral encoding in silent and noisy conditions provides insight into how resistant such encoding is to noisy backgrounds, a critical benchmark when selecting an auditory alerting stimulus for application in field settings.

The activity of the auditory system is commonly characterized using a specific property of the AEP called the ABR (Dooling and Fay, 2000; see McGee *et al.*, 2019, for information on raptor AEPs). The ABR measures the neural response to the onset of a sound, either a tone, click or other stimuli. While the ABR offers an important index of the functioning of the auditory brainstem, behaviourally relevant auditory perception is better tied to more in-depth properties such as processing of the frequency properties of sounds as indexed by the frequency following response and amplitude properties of sound as indexed by the EFR (Moore, 2019). The results shown here for eagles underscore the value of a focus on these more complex descriptions of the processing of a wide variety of stimuli.

We found interspecific differences between the bald and golden eagles in their ability to process FM sounds like linear frequency sweeps and sinusoidal FM sounds. Bald eagle ability to process rapid FM sounds rivalled that of the best songbirds we have measured to date (J.R. Lucas, K.S. Henry and M.D. Gall, unpubl. data), while golden eagle auditory processing for rapid FM sounds was relatively poor. These patterns likely reflect, in part, differences between the eagle species in their ‘auditory filter widths’ (see Henry and Lucas, 2010). The peripheral auditory system is arranged in a spatially graded series of frequency-tuned channels. The bandwidth of these channels (the auditory filter widths) determines a fundamental tradeoff in the auditory system: narrow filters result in good frequency resolution (i.e. ability to distinguish similar frequencies) but poor temporal resolution (i.e. measurement of rapidly changing frequencies or amplitude), and broad filters result in poor frequency resolution but good temporal resolution (Moore, 1993). This is because tones can be differentiated only if they fall on different filters. Therefore, narrow filters result in good frequency resolution. However, narrow filters are poor at temporal resolution because the auditory system needs to integrate information over relatively long periods of time in order to measure frequency accurately (Viemeister and Plack, 1993). In contrast, wide filters are poor at frequency resolution because similar tones that fall within the filter will not be resolvable. Wide filters are good at temporal resolution because their integration times are relatively short (Henry *et al.*, 2011). Auditory filter bandwidth has been shown to correlate with species-specific FM and AM song properties in song birds (Henry and Lucas, 2010) and with processing of host-specific songs in brown-headed cowbirds (Gall and Lucas, 2010).

The substantially better processing of rapid frequency modulation (frequency sweeps and sinusoidal frequency modulation) by bald compared with golden eagles would be consistent with golden eagles having narrower auditory filters than bald eagles (see Henry and Lucas, 2010; Henry *et al.*, 2011). Bald eagles also showed better processing of the two stimuli that consisted of fairly rapid amplitude modulation: the 600 Hz AM component in the harmonic stack with a missing fundamental and the AM stimuli generated with a carrier frequency and side bands. Apparent differences in auditory filters of these eagle species would mirror results in passerines and suggest that bald and golden eagles may have evolved different hearing capabilities reflecting differences in their vocal complexity or social structure (see Henry *et al.*, 2016). However, auditory processing of prey or threat cannot be ruled out (e.g. Köppl *et al.*, 1993). For physiology-based design of field eagle alerting stimuli, such differences between species become critical because dynamic sounds were found to be more resistant to noise masking but less tuned to golden eagle sensitivity.

The mechanism associated with reduced noise effects on processing of rapid FM signals can also be partly attributed to processing of sound in auditory filters. The auditory filters are tonotopically organized on the basilar papilla—the membrane that holds auditory hair cells that convert the mechanical oscillation of sound into a neural signal in the inner ear (Møller, 2006). The result of this tonotopic organization is that different frequencies stimulate different parts of the basilar papilla. This is important for noise effects because any given tone stimulating an auditory filter will be hard to differentiate from the specific frequency of the noise entering that auditory filter, causing the noise to mask the detection of the tone (Moore, 1993). However, processing of sounds in noise is mitigated if the amplitude profile of the tone is different than the amplitude profile of the noise, which will occur with rapid frequency modulation for the following reason: as a rapidly FM signal sweeps across the membrane, it will sequentially stimulate auditory filters for short periods of time (Moore and Sek, 1995; Whiteford *et al.*, 2020). The result is that the FM sweep causes the signal to be converted from a FM signal to a series of rapid amplitude modulations. This can account for our result that evoked potentials from both eagle species are least impacted by FM signals and maximally impacted by static tones. Similar results have been demonstrated in processing of sounds in humans (Henry and Heinz, 2013; Cabrera and Werner, 2017; Shen and Souza, 2017). We therefore suggest that the best compromise is to select dynamic sounds with intermediate rates of change to maximize stimulation of the golden eagle auditory system in noise.

Using sensory physiological studies to determine candidate stimuli for target species is an approach that allows for selection of targeted sensory stimuli and at a minimum helps to avoid implementing stimuli that cannot be perceived by the target animals (Lim *et al.*, 2008; Blumstein and Fernández-Juricic, 2010). For example, visual physiology and genetic research has shown that falcon and accipiter raptor eyes, including golden eagle eyes, filter out ultraviolet light and/or do not have ultraviolet-sensitive photoreceptors (Doyle *et al.*, 2014; Lind *et al.*, 2014; Olsson *et al.*, 2021), therefore making it unlikely that raptors would respond to ultraviolet light stimuli or deterrents (Hunt *et al.*, 2015; May *et al.*, 2017). Similarly, we found sensory differences between the bald and golden eagles and between different types of sounds in noisy backgrounds, which collectively suggest that sound selection is important for development of effective alerting stimuli. Complex sounds are less impacted by noisy conditions, but it is critical to avoid using a stimulus sound that cannot be effectively processed by the target animals (e.g. fast sinusoidally FM for a golden eagle). There are, however, important additional considerations that could influence the effectiveness of a sound stimulus and are not addressed in this study. Sound attenuation increases with distance from a wind turbine or the source of a sound. We did not study how our stimulus sounds attenuated with distance in different noise conditions. Similarly, peripheral auditory processing is only the first step in a behavioural response to a sound stimulus and can only suggest which sounds may, or may not, be likely to alert the animal. Candidate signals require testing in behavioural assays to determine whether they produce the desired alert or avoidance behaviours and to determine if these effects are robust over time (see Gilmour *et al.*, 2020; Boycott *et al.*, 2021). Ultimately, the approach and results described in this manuscript can inform the development of alerting stimuli by ruling out poor candidate signals, but cannot replace field testing to determine how best to deter wildlife from interacting with wind energy facilities.

## Funding

This material is based upon work supported by the U.S. Department of Energy’s Office of Energy Efficiency and Renewable Energy (EERE) under the Wind Energy—Understanding the Eagle Sensory World to Enhance Detection and Response to Wind Turbines, Award Number DE-EE0007882.

The EERE award was supplemented with cost sharing from Avangrid Renewables (awarded to E.F.-J., T.K. and J.R.L.).

*Disclaimer* This report was prepared as an account of work sponsored by an agency of the U.S. Government. Neither the U.S. Government nor any agency thereof, nor any of their employees, makes any warranty, express or implied, or assumes any legal liability or responsibility for the accuracy, completeness or usefulness of any information, apparatus, product or process disclosed, or represents that its use would not infringe privately owned rights. Reference herein to any specific commercial product, process or service by trade name, trademark, manufacturer or otherwise does not necessarily constitute or imply its endorsement, recommendation or favouring by the U.S. Government or any agency thereof. Any use of trade, firm or product names is for descriptive purposes only and does not imply endorsement by the U.S. Government. The findings and conclusions in this article are those of the authors and do not necessarily represent the views of the Department of Energy. This manuscript has been peer reviewed and approved for publication consistent with USGS Fundamental Science Practices (https://pubs.usgs.gov/circ/1367/).

## Data Accessibility

Data collected for this study are stored at the following website: https://osf.io/uzgqs/. The SAS code used to analyse these data is listed in Appendix 5.

## Supplementary Material

Web_Material_coac059
