# Supplementary material for: Selecting auditory alerting stimuli for eagles on the basis of auditory evoked potentials
Source: Conserv Physiol. 2022 Sep 16;10(1):coac059. doi: 10.1093/conphys/coac059 (PMC9486983; doi:10.1093/conphys/coac059)
Supplement: Web_Material_coac059 [file web_material_coac059.zip › Goller et al.Appdx 4-6.docx]

**Appendix 4.** Noise floor analyses for static stimuli

## **Noise Floor**

## We tested levels of phase-locking in the main text for static stimuli (pure tones, tone stacks and AM stimuli), where phase-locking was measured relative the noise floor of the AEPs (note: this is not related to the noise background for each stimulus). We need to estimate phase-locking relative to the AEP noise floor because the AEP noise floor is expected to vary with background noise levels. As such, the magnitude of phase-locking would be misinterpreted if the level of the AEP noise floor was not accounted for. For example, assume we are measuring the magnitude of phase-locking for a 1 kHz tone. Assume that the peak phase-locking magnitude is -30 dBV at 1 kHz. If the AEP noise floor is -80 dBV, then we could use -50 dBV as an index of the magnitude of phase-locking to that tone. Alternatively, if the AEP noise floor is -30 dBV, then the implication is that the auditory brainstem shows no phase-locking to that tone because the ‘peak’ at 1 kHz is indistinguishable from the noise floor.

## Here we provide estimates of the magnitude of the noise floor for each set of static stimuli. These results reflect the use of a single average noise floor for each bird. We used repeated measures linear mixed models (Proc Mixed in SAS v9.4) to test for species, age and noise-background type (none, white and pink noise) effects on the noise floor. All interaction terms were initially included in the statistical model and non-significant interaction terms were removed in order of increasing F value. Only the final models with all main effects and significant interaction terms (α=0.05) are presented here. Estimates presented are least squares means and standard errors calculated from the final statistical model for each stimulus (Proc Mixed, LSMEANS).

## **Tones**

There was no significant species main effect (F_1,5_ = 1.11, P = 0.34) or age main effect (F_1,5_ = 0.34, P = 0.59) on the magnitude of the noise floor with pure-tone treatments. In contrast, the noise background had a significant effect on the magnitude of the AEP noise floor (F_2,14_=108.7, P<0.0001). Specifically, the AEP noise floor with no noise background (-91.02 ± 1.58 dBV) was lower than the noise floor for both white noise (-78.96 ± 1.58 dBV; t_14_=10.93, P<0.0001) and pink noise (-75.52 ± 1.58 dBV; t_14_=15.03, P<0.0001). The AEP noise floor for white was also lower than the AEP noise floor for pink noise (t_14_=3.11, P=0.008).

## **Tone stacks**

### Harmonic stack

There was no significant species main effect (F_1,5_ = 0.23, P = 0.65) or age main effect (F_1,5_ = 0.00, P = 0.99) on the magnitude of the noise floor. In contrast, the noise background had a significant effect on the AEP noise floor (F_2,14_=4.83, P=0.025), with no noise (-72.94 ± 2.57 dBV) lower than both white noise (-70.32 ± 2.57 dBV; t_14_=2.62, P=0.020) and pink noise (-68.78 ± 2.57 dBV; t_14_=3.02, P=0.009). The AEP noise floor was similar for white compared to pink noise (t_14_=1.54, P=0.15).

### Mistuned stack

There was no significant species main effect (F_1,5_ = 1.11, P = 0.34) or age main effect (F_1,5_ = 1.40, P = 0.29) on the magnitude of the noise floor. In contrast, the noise background had a significant effect on the magnitude of the AEP noise floor (F_2,14_=3.86, P=0.046), with no noise (-69.66 ± 2.05 dBV) lower than both white noise (-67.11 ± 2.05 dBV; t_14_=2.16, P=0.049) and pink noise (-65.32 ± 2.05 dBV; t_14_=2.74, P=0.016). The AEP noise floor was similar for white compared to pink noise (t_14_=1.51, P=0.15).

Missing fundamental (600 Hz) stack

There were no significant main effects on the magnitude of the noise floor (species: F_1,5_ = 0.07, P = 0.81; age: F_1,5_ = 0.05, P = 0.83; noise background: F_2,14_ = 2.62, P = 0.10).

## **Amplitude modulated (AM) stimuli**

There was no significant species main effect (F_1,5_ = 0.94, P = 0.38) or age main effect (F_1,5_ = 0.04, P = 0.85) on the magnitude of the noise floor. In contrast, the noise background had a significant effect on the AEP noise floor (F_2,14_=28.08, P<0.0001), with no noise (-69.08 ± 1.63 dBV) lower than both white noise (-65.85 ± 1.63 dBV; t_14_=5.50, P<0.0001) and pink noise (-64.87 ± 1.63 dBV; t_14_=7.16, P<0.0001). The AEP noise floor was similar for white compared to pink noise (t_14_=1.65, P=0.12).

**Appendix 5. SAS Code For Analyses of AEPs Measured for All Stimuli (each stimulus required a different set of code)**

**SAS Code For Analysis of Tones in Noise**

%let y=1000; /* beginning number */

%let xx=3000; /* ending number */

%let ne=8;

%let z1 = z:\*FOLDER*\eagles\eagle1a\noise tones\;

%let z2 = z:\*FOLDER*\eagles\eagle2\noise tones\;

%let z3 = z:\*FOLDER*\eagles\eagle3\noise tones\;

%let z4 = z:\*FOLDER*\eagles\eagle4\noise tones\;

%let z5 = z:\*FOLDER*\eagles\2018-09-06 WCV BAEA105\noise tones\;

%let z6 = z:\*FOLDER*\eagles\2019-02-09 LW GOEA107\noise tones\;

%let z7 = z:\*FOLDER*\eagles\2019-02-09 LW GOEA108\noise tones\;

%let z8 = z:\*FOLDER*\eagles\2019-02-09 LW BAEA107\noise tones\;

%let w = .txt;

symbol1 c=green v=dot i=join line=1 height=2 width=4;

symbol2 c=blue v=box i=join line=2 height=3 width=4;

symbol3 c=red v=z i=join line=3 width=4;

symbol4 c=black v=star i=join line=2 width=4;

symbol5 c=green v=plus i=join line=2 width=4;

symbol6 c=blue v=square i=join line=2 width=4;

%macro allthem;

%let i=%eval(0);

%do zz=1 %to &ne;

%do pz=1 %to 6;

%if &pz=1 %then %let p= 500;

%else %if &pz=2 %then %let p = 1000;

%else %if &pz=3 %then %let p = 2000;

%else %if &pz=4 %then %let p = 3000;

%else %if &pz=5 %then %let p = 4000;

%else %let p = 5000;

%do pp=1 %to 3;

%if &pp=1 %then %let qq= _60_;

%else %if &pp=2 %then %let qq = _70_;

%else %let qq = _80_;

%do ll=1 %to 3;

%if &ll=1 %then %let Zx = 1_;

%else %if &ll=2 %then %let Zx = 4_;

%else %let Zx = 5_;

%let i=%eval(&i+1);

%if &zz=1 %then %do;

%let q = FULLspec_baea1_;

%let filenm = &q&p&qq&zx&w;

%let R = "&z1&q&p&qq&zx&w";

%end;

%else %if &zz=2 %then %do;

%let q = FULLspec_baea2_;

%let filenm = &q&p&qq&zx&w;

%let R = "&z2&q&p&qq&zx&w";

%end;

%else %if &zz=3 %then %do;

%let q = FULLspec_baea103_;

%let filenm = &q&p&qq&zx&w;

%let R = "&z3&q&p&qq&zx&w";

%end;

%else %if &zz=4 %then %do;

%let q = FULLspec_baea104_;

%let filenm = &q&p&qq&zx&w;

%let R = "&z4&q&p&qq&zx&w";

%end;

%else %if &zz=5 %then %do;

%let q = FULLspec_baea105_;

%let filenm = &q&p&qq&zx&w;

%let R = "&z5&q&p&qq&zx&w";

%end;

%else %if &zz=6 %then %do;

%let q = FULLspec_goea107_;

%let filenm = &q&p&qq&zx&w;

%let R = "&z6&q&p&qq&zx&w";

%end;

%else %if &zz=7 %then %do;

%let q = FULLspec_goea108_;

%let filenm = &q&p&qq&zx&w;

%let R = "&z7&q&p&qq&zx&w";

%end;

%else %if &zz=8 %then %do;

%let q = FULLspec_baea107_;

%let filenm = &q&p&qq&zx&w;

%let R = "&z8&q&p&qq&zx&w";

%end;

%put &i; %put &zz; %put &p; %put &q; %put &qq; %put &zx;

filename onesec &r;

data spect (drop=r filename undersc) ; infile onesec ;

attrib filen length=$30; attrib filename length=$30;

filen="&filenm";

spec=substrn(filen,10,4);

*FULLspec_baea103_500_60_1_;

hz=&zz;

if hz>2 then do;

eagle=substrn(filen,10,7);

undersc=substrn(filen,21,1); put 'undersc: ' undersc;

if undersc='_' then do; tone=1.0 * substrn(filen,18,3); tonedb=1.0 * substrn(filen,22,2);

noisef=1.0 * substrn(filen,25,1); end;

else do; tone=1.0 * substrn(filen,18,4); tonedb=1.0 * substrn(filen,23,2);

noisef=1.0 *substrn(filen,26,1); end; end;

else do;

eagle=substrn(filen,10,5);

undersc=substrn(filen,19,1); put 'undersc: ' undersc;

if undersc='_' then do; tone=1.0 * substrn(filen,16,3); tonedb=1.0 * substrn(filen,20,2);

noisef=1.0 * substrn(filen,23,1); end;

else do; tone=1.0 * substrn(filen,16,4); tonedb=1.0 * substrn(filen,21,2);

noisef=1.0 *substrn(filen,24,1); end; end;

input; input; input; input r $ r $ xmin;

input r$ r $ xmax;

input r $ r $ nx;

input r $ r $ dx;

input; input; input; input; input; input; input; input;

do i=1 to nx;

input r $ r $ r $ r $ db; freq=xmin+dx*(i-1);

if eagle='beae1' then do; if tone ne 4000 then output;end;

else output;

end;

input filename $ ; put 'filename: ' filename ' ' filen; run;

%if &i=1 %then %do; data spectra; set spect; run; %end;

%else %do; data spectra; set spectra spect; %end;

%end;

%end; %end; %end;

%MEND allthem; %allthem;

data spectra; set spectra;

age='adult';

if eagle='baea1' then lead=0.23;

else if eagle='baea2' then do; lead=0.0; age='juvie'; end;

else if eagle='baea103' then lead=0.102;

else if eagle='baea104' then lead=0.086;

else if eagle='baea105' then do; lead=0.041; age='juvie'; end;

else if eagle='goea107' then do; lead=0.275; age='juvie'; end;

else if eagle='goea108' then do; lead=.; age='adult'; end;

else if eagle='baea107' then do; lead=.; age='adult'; end;

else put 'eagle: ' eagle; run;

data tone; set spectra;

if freq>(tone+100) or freq<(tone-100) then delete; run;

proc sort data=tone; by spec eagle tone tonedb noisef;

proc means data=tone noprint; by spec eagle tone tonedb noisef; var db; id freq lead age;

output out=meanz max=maxdb; run;

data meanz(drop=_type_ _freq_); set meanz;run;

data noise; set spectra;

if freq>7000 then delete;

if freq> (tone-100) and freq<(tone+100) then delete;

if freq>3000+freq then delete; if freq<3000-freq then delete;run;

proc sort ; by spec eagle age lead tone tonedb noisef ;

proc mixed data=noise; by spec eagle age lead tone tonedb noisef;

model db = freq freq*freq/ outp=preds; run;

proc plot data=preds; plot resid*pred;

proc univariate data=preds plot normal; var resid;run;

proc means data=preds; var freq; run;

data preds(drop=xmin xmax nx df alpha lower upper dx); set preds;

if resid<-15 or resid>15 then delete; run;

proc sort data=preds; by eagle tone tonedb noisef;

proc means noprint data=preds noprint; by eagle tone tonedb noisef; var resid; id pred lead age;

output out=noiz uclm=uclm p75=p75 p90=p90 p95=p95; run;

data noiz(drop=_type_ _freq_); set noiz; run;

data final; merge noiz meanz ; by eagle tone tonedb noisef;

relpeak=maxdb - (pred+p75); if relpeak<0 then relpeak=0; run;

/* FINAL MODEL */

proc mixed data=final; class spec eagle tone tonedb noisef age;

model relpeak = tone tonedb noisef spec age tone*tonedb tone*noisef tonedb*spec /*tone*spec*/

age*tone tonedb*noisef noisef*spec noisef*age tonedb*noisef

/* tonedb*age spec*tone*tonedb spec*tone*noisef spec*age*tone */ spec*tonedb*noisef

spec*age spec*noisef*age tone*tonedb*noisef /* spec*tonedb*age*/

/solution outp=preds ddfm=bw /*KR*/;

lsmeans tone tonedb noisef spec age spec*tonedb*noisef spec*noisef*age tone*tonedb*noisef/*/diff*/;

repeated /type=ar(1) subject=eagle; title 'tone in noise'; run;

proc plot data=preds; plot resid*pred;

proc univariate data=preds plot normal; var resid;run;

* PLOT OUT 3-WAY INTERACTIONS *;

proc sort data=preds; by spec tonedb noisef;

proc means noprint data=preds; by spec tonedb noisef; var relpeak;

output out=azl mean=xrelpeak; run;

proc print; run;

proc sort data=azl; by tonedb;

proc gplot data=azl; by tonedb; plot xrelpeak*noisef=spec; run;

proc sort data=preds; by spec noisef age;

proc means noprint data=preds; by spec noisef age; var relpeak;

output out=azl mean=xrelpeak; run;

proc print; run;

proc sort data=azl; by age;

proc gplot data=azl; by age; plot xrelpeak*noisef=spec; run;

proc sort data=preds; by tone tonedb noisef;

proc means noprint data=preds; by tone tonedb noisef; var relpeak;

output out=azl mean=xrelpeak; run;

proc print; run;

proc sort data=azl; by tonedb;

proc gplot data=azl; by tonedb; plot xrelpeak*tone=noisef; run;

**SAS Code For Analysis of Harmonic Stacks**

%let z = z:\*FOLDER*\eagles\eagle1\noise stacks\;

%let z1 = z:\*FOLDER*\eagles\eagle1a\noise stacks\;

%let z2 = z:\*FOLDER*\eagles\eagle2\noise stacks\;

%let z3 = z:\*FOLDER*\eagles\eagle3\noise stacks\;

%let z4 = z:\*FOLDER*\eagles\eagle4\noise stacks\;

%let z5 = z:\*FOLDER*\eagles\2018-09-06 WCV BAEA105\noise stacks\;

%let z6 = z:\*FOLDER*\eagles\2019-02-09 LW GOEA107\noise stacks\;

%let z7 = z:\*FOLDER*\eagles\2019-02-09 LW GOEA108\noise stacks\;

%let z8 = z:\*FOLDER*\eagles\2019-02-09 LW BAEA107\noise stacks\;

%let ne=8; *NUMBER OF EAGLES;

%let w = .txt;

%let y=1; /* beginning number */

%let xx=9; /* ending number */

symbol1 c=green v=dot i=join line=1 height=2 width=4;

symbol2 c=blue v=box i=join line=2 height=3 width=4;

symbol3 c=red v=z i=join line=3 width=4;

symbol4 c=black v=star i=join line=2 width=4;

symbol5 c=green v=plus i=join line=2 width=4;

symbol6 c=blue v=square i=join line=2 width=4;

%macro allthem;

%let i=%eval(0);

%do zz=1 %to &ne;

%do p=&y %to &xx;

%if &zz=1 %then %do;

%if &p= 1 %then %let Q=FULLspec_HarmonicStacbaea1_1000_2000_80_1_;

%else %if &p= 2 %then %let Q=FULLspec_HarmonicStacbaea1_1000_2000_80_4_;

%else %if &p= 3 %then %let Q=FULLspec_HarmonicStacbaea1_1000_2000_80_5_;

%else %if &p= 4 %then %let Q=FULLspec_HarmonicStacbaea1_1000_2200_80_1_;

%else %if &p= 5 %then %let Q=FULLspec_HarmonicStacbaea1_1000_2200_80_4_;

%else %if &p= 6 %then %let Q=FULLspec_HarmonicStacbaea1_1000_2200_80_5_;

%else %if &p= 7 %then %let Q=FULLspec_HarmonicStacbaea1_1200_1800_80_1_;

%else %if &p= 8 %then %let Q=FULLspec_HarmonicStacbaea1_1200_1800_80_4_;

%else %if &p= 9 %then %let Q=FULLspec_HarmonicStacbaea1_1200_1800_80_5_;

%let R = "&Z1&Q&W"; %end;

%else %if &zz=2 %then %do;

%if &p=1 %then %let Q=FULLspec_HarmonicStacbaea2_1000_2000_80_1_;

%else %if &p=2 %then %let Q=FULLspec_HarmonicStacbaea2_1000_2000_80_4_;

%else %if &p=3 %then %let Q=FULLspec_HarmonicStacbaea2_1000_2000_80_5_;

%else %if &p=4 %then %let Q=FULLspec_HarmonicStacbaea2_1000_2200_80_1_;

%else %if &p=5 %then %let Q=FULLspec_HarmonicStacbaea2_1000_2200_80_4_;

%else %if &p=6 %then %let Q=FULLspec_HarmonicStacbaea2_1000_2200_80_5_;

%else %if &p=7 %then %let Q=FULLspec_HarmonicStacbaea2_1200_1800_80_1_;

%else %if &p=8 %then %let Q=FULLspec_HarmonicStacbaea2_1200_1800_80_4_;

%else %if &p=9 %then %let Q=FULLspec_HarmonicStacbaea2_1200_1800_80_5_;

%let R = "&Z2&Q&W"; %end;

%else %if &zz=3 %then %do;

%if &p=1 %then %let Q=FULLspec_HarmonicStacbaea103_1000_2000_80_1_;

%else %if &p=2 %then %let Q=FULLspec_HarmonicStacbaea103_1000_2000_80_4_;

%else %if &p=3 %then %let Q=FULLspec_HarmonicStacbaea103_1000_2000_80_5_;

%else %if &p=4 %then %let Q=FULLspec_HarmonicStacbaea103_1000_2200_80_1_;

%else %if &p=5 %then %let Q=FULLspec_HarmonicStacbaea103_1000_2200_80_4_;

%else %if &p=6 %then %let Q=FULLspec_HarmonicStacbaea103_1000_2200_80_5_;

%else %if &p=7 %then %let Q=FULLspec_HarmonicStacbaea103_1200_1800_80_1_;

%else %if &p=8 %then %let Q=FULLspec_HarmonicStacbaea103_1200_1800_80_4_;

%else %if &p=9 %then %let Q=FULLspec_HarmonicStacbaea103_1200_1800_80_5_;

%let R = "&Z3&Q&W"; %end;

%else %if &zz=4 %then %do;

%if &p=1 %then %let Q=FULLspec_HarmonicStacbaea104_1000_2000_80_1_;

%else %if &p=2 %then %let Q=FULLspec_HarmonicStacbaea104_1000_2000_80_4_;

%else %if &p=3 %then %let Q=FULLspec_HarmonicStacbaea104_1000_2000_80_5_;

%else %if &p=4 %then %let Q=FULLspec_HarmonicStacbaea104_1000_2200_80_1_;

%else %if &p=5 %then %let Q=FULLspec_HarmonicStacbaea104_1000_2200_80_4_;

%else %if &p=6 %then %let Q=FULLspec_HarmonicStacbaea104_1000_2200_80_5_;

%else %if &p=7 %then %let Q=FULLspec_HarmonicStacbaea104_1200_1800_80_1_;

%else %if &p=8 %then %let Q=FULLspec_HarmonicStacbaea104_1200_1800_80_4_;

%else %if &p=9 %then %let Q=FULLspec_HarmonicStacbaea104_1200_1800_80_5_;

%let R = "&Z4&Q&W"; %end;

%else %if &zz=5 %then %do;

%if &p=1 %then %let Q=FULLspec_HarmonicStacbaea105_1000_2000_80_1_;

%else %if &p=2 %then %let Q=FULLspec_HarmonicStacbaea105_1000_2000_80_4_;

%else %if &p=3 %then %let Q=FULLspec_HarmonicStacbaea105_1000_2000_80_5_;

%else %if &p=4 %then %let Q=FULLspec_HarmonicStacbaea105_1000_2200_80_1_;

%else %if &p=5 %then %let Q=FULLspec_HarmonicStacbaea105_1000_2200_80_4_;

%else %if &p=6 %then %let Q=FULLspec_HarmonicStacbaea105_1000_2200_80_5_;

%else %if &p=7 %then %let Q=FULLspec_HarmonicStacbaea105_1200_1800_80_1_;

%else %if &p=8 %then %let Q=FULLspec_HarmonicStacbaea105_1200_1800_80_4_;

%else %if &p=9 %then %let Q=FULLspec_HarmonicStacbaea105_1200_1800_80_5_;

%let R = "&Z5&Q&W"; %end;

%else %if &zz=6 %then %do;

%if &p=1 %then %let Q=FULLspec_HarmonicStacgoea107_1000_2000_80_1_;

%else %if &p=2 %then %let Q=FULLspec_HarmonicStacgoea107_1000_2000_80_4_;

%else %if &p=3 %then %let Q=FULLspec_HarmonicStacgoea107_1000_2000_80_5_;

%else %if &p=4 %then %let Q=FULLspec_HarmonicStacgoea107_1000_2200_80_1_;

%else %if &p=5 %then %let Q=FULLspec_HarmonicStacgoea107_1000_2200_80_4_;

%else %if &p=6 %then %let Q=FULLspec_HarmonicStacgoea107_1000_2200_80_5_;

%else %if &p=7 %then %let Q=FULLspec_HarmonicStacgoea107_1200_1800_80_1_;

%else %if &p=8 %then %let Q=FULLspec_HarmonicStacgoea107_1200_1800_80_4_;

%else %if &p=9 %then %let Q=FULLspec_HarmonicStacgoea107_1200_1800_80_5_;

%let R = "&Z6&Q&W"; %end;

%else %if &zz=7 %then %do;

%if &p=1 %then %let Q=FULLspec_HarmonicStacgoea108_1000_2000_80_1_;

%else %if &p=2 %then %let Q=FULLspec_HarmonicStacgoea108_1000_2000_80_4_;

%else %if &p=3 %then %let Q=FULLspec_HarmonicStacgoea108_1000_2000_80_5_;

%else %if &p=4 %then %let Q=FULLspec_HarmonicStacgoea108_1000_2200_80_1_;

%else %if &p=5 %then %let Q=FULLspec_HarmonicStacgoea108_1000_2200_80_4_;

%else %if &p=6 %then %let Q=FULLspec_HarmonicStacgoea108_1000_2200_80_5_;

%else %if &p=7 %then %let Q=FULLspec_HarmonicStacgoea108_1200_1800_80_1_;

%else %if &p=8 %then %let Q=FULLspec_HarmonicStacgoea108_1200_1800_80_4_;

%else %if &p=9 %then %let Q=FULLspec_HarmonicStacgoea108_1200_1800_80_5_;

%let R = "&Z7&Q&W"; %end;

%else %if &zz=8 %then %do;

%if &p=1 %then %let Q=FULLspec_HarmonicStacbaea107_1000_2000_80_1_;

%else %if &p=2 %then %let Q=FULLspec_HarmonicStacbaea107_1000_2000_80_4_;

%else %if &p=3 %then %let Q=FULLspec_HarmonicStacbaea107_1000_2000_80_5_;

%else %if &p=4 %then %let Q=FULLspec_HarmonicStacbaea107_1000_2200_80_1_;

%else %if &p=5 %then %let Q=FULLspec_HarmonicStacbaea107_1000_2200_80_4_;

%else %if &p=6 %then %let Q=FULLspec_HarmonicStacbaea107_1000_2200_80_5_;

%else %if &p=7 %then %let Q=FULLspec_HarmonicStacbaea107_1200_1800_80_1_;

%else %if &p=8 %then %let Q=FULLspec_HarmonicStacbaea107_1200_1800_80_4_;

%else %if &p=9 %then %let Q=FULLspec_HarmonicStacbaea107_1200_1800_80_5_;

%let R = "&Z8&Q&W"; %end;

filename onesec &r;

%put &zz &p &r; %put;

data spect (drop=r filename ) ; infile onesec ;

attrib filen length=$50; attrib filename length=$50; attrib eagle length=$7;

filen="&q";

xz=substrn(filen,27,1);

if xz='_' then do;

eagle=substrn(filen,22,5);

freq1=1.0 * substrn(filen,28,4); f2=1.0*substrn(filen,33,2);

dblevel=1.0 * substrn(filen, 38,2);

noisef=1.0 *substrn(filen,41,1); spec=substrn(filen,22,4); end;

else do;

eagle=substrn(filen,22,7);

freq1=1.0 * substrn(filen,30,4); f2=1.0*substrn(filen,35,2);

dblevel=1.0 * substrn(filen, 40,2);

noisef=1.0 *substrn(filen,43,1); spec=substrn(filen,22,4);end;

if freq1=1000 and f2=20 then do; ftype='1k stack'; freq2=2000; freq3=3000; freq4=4000; freq5=5000; end;

else if freq1=1000 and f2=22 then do; ftype='mistuned harm'; freq2=2200; freq3=3300; freq4=3600; freq5=4700; end;

else do; ftype='miss. fund'; freq2=1800; freq3=2400; freq4=3000; freq5=600; end;

put ' freq1 dblevel noisef: ' eagle filen freq1 dblevel noisef;

input; input; input; input r $ r $ xmin;

input r$ r $ xmax;

input r $ r $ nx;

input r $ r $ dx;

input; input; input; input; input; input; input; input;

do i=1 to nx;

input r $ r $ r $ r $ db; freq=xmin+dx*(i-1);

output; end;

input filename $ ; put 'filename: ' filename ' ' filen; run;

%if &zz=1 %then %do; %if &p=&y %then %do; data spectra; set spect; run; %end;

%else %do; data spectra; set spectra spect; %end; %end;

%else %do; data spectra; set spectra spect; %end; %end; %end;

%MEND allthem; %allthem;

********************************************************************************;

data spectra; set spectra;

age='adult';

if eagle='baea2' then do; age='juvie'; end;

else if eagle='baea105' then do; age='juvie'; end;

else if eagle='goea107' then do; age='juvie'; end;

else if eagle='goea108' then do; age='adult'; end;

else if eagle='baea107' then do; age='adult'; end;

else put 'eagle: ' eagle; run;

data carrier; set spectra;

if (freq<(freq1+25) and freq>(freq1-25)) then signal='freq1';

else if (freq<(freq2+25) and freq>(freq2-25)) then signal='freq2';

else if (freq<(freq3+25) and freq>(freq3-25)) then signal='freq3';

else if (freq<(freq4+25) and freq>(freq4-25)) then signal='freq4';

else if (freq<(freq5+25) and freq>(freq5-25)) then signal='freq5';

else delete; run;

proc sort data=carrier; by spec eagle freq1 ftype f2 noisef signal;

proc means data=carrier noprint; by spec eagle freq1 ftype f2 noisef signal; var db; id freq age;

output out=meanz max=maxdb; run;

data meanz(drop=_type_ _freq_); set meanz; run;

proc sort data=spectra; by spec eagle freq1 ftype f2 noisef; run;

data noise; set spectra;

if freq>500 and freq<5000;

if (freq<(freq1+100) and freq>(freq1-100)) then delete;

else if (freq<(freq2+100) and freq>(freq2-100)) then delete;

else if (freq<(freq3+100) and freq>(freq3-100)) then delete;

else if (freq<(freq4+100) and freq>(freq4-100)) then delete;

else if freq5>. then do; if (freq<(freq5+100) and freq>(freq5-100)) then delete;

end; run;

proc sort ; by spec eagle freq1 ftype f2 noisef ;

proc mixed data=noise; by spec eagle freq1 ftype f2 noisef;

model db = freq freq*freq/ outp=preds; run;

proc plot data=preds; plot resid*pred;

proc univariate data=preds plot normal; var resid;run;

data preds(drop=xmin xmax nx df alpha lower upper dx); set preds;

if resid<-15 or resid>15 then delete; run;

proc gplot; by eagle freq1 ftype f2 noisef; plot resid*freq; plot pred*freq; run;

proc sort data=preds; by spec eagle freq1 ftype f2 noisef;

proc means noprint data=preds noprint; by spec eagle freq1 ftype f2 noisef; var resid; id pred;

output out=noiz uclm=uclm p90=p90 p95=p95; run;

data noiz(drop=_type_ _freq_); set noiz; run;

data final; merge noiz meanz ; by spec eagle freq1 ftype f2 noisef;

relpeak=maxdb - (pred+p95);

if relpeak<0 then relpeak=0;

sqrtpeak=sqrt(relpeak);run;

proc print; run;

proc sort data=final; by ftype signal noisef;

proc means noprint data=final noprint; by ftype signal noisef; var relpeak;

output out=var mean=xpeak cv=cvpeak stddev=stdpeak uclm=uclm p90=p90 p95=p95; run;

data var(drop=_freq_ _type_); set var;

proc print; run;

proc sort data=var; by ftype signal noisef;

proc gplot data=var; by ftype ; plot stdpeak*signal=noisef /*/vaxis=0 to 25 by 5*/;

plot cvpeak*signal=noisef; plot stdpeak*xpeak=noisef; run;

/* FINAL MODEL */

data missfu; set final; if ftype='miss. fu'; run;

proc sort data=missfu; by ftype eagle noisef signal ; run;

proc mixed data=missfu; by ftype; class spec eagle signal noisef age;

model relpeak =signal noisef age spec

signal*spec /*age*spec age*signal age*noisef noisef*spec signal*noisef */

/*signal*age*spec noisef*age*spec signal*noisef*spec */

/solution outp=preds ddfm=bw ;

lsmeans signal noisef age spec signal*spec /diff;

repeated /type=cs subject=eagle; title 'miss. fund'; run;

proc plot data=preds; plot resid*pred;

proc univariate data=preds plot normal; var resid;run;

proc sort data=preds; by signal spec;

proc means data=preds; by signal spec; var relpeak;

output out=azl mean=xrelpeak; run;

proc gplot; plot xrelpeak*signal=spec; run;

/* FINAL MODEL */

data onek; set final; if ftype='1k stack'; run;

proc sort data=onek; by ftype eagle signal noisef; run;

proc mixed data=onek; by ftype; class spec eagle signal noisef age;

model relpeak =signal noisef age spec

noisef*spec age*noisef /*signal*noisef signal*spec age*spec age*signal*/

/*noisef*age*spec signal*noisef*spec */

/solution outp=preds ddfm=bw /*KR*/;

lsmeans signal noisef age spec age*noisef noisef*spec /diff;

repeated /type=ar(1) subject=eagle; title '1k stack'; run;

proc plot data=preds; plot resid*pred;

proc univariate data=preds plot normal; var resid;run;

proc sort data=preds; by signal spec age;

proc means data=preds; by signal spec age; var relpeak;

output out=azl mean=xrelpeak; run;

proc sort data=azl; by age;

proc gplot; by age; plot xrelpeak*signal=spec; run;

proc sort data=preds; by noisef age;

proc means data=preds; by noisef age; var relpeak;

output out=azl mean=xrelpeak; run;

proc gplot; plot xrelpeak*noisef=age; run;

proc sort data=preds; by noisef spec;

proc means data=preds; by noisef spec; var relpeak;

output out=azl mean=xrelpeak; run;

proc gplot; by age; plot xrelpeak*noisef=spec; run;

/* FINAL MODEL */

data mist; set final; if ftype='mistuned'; run;

proc sort data=mist; by ftype eagle noisef signal ; run;

proc mixed data=mist; by ftype; class spec eagle noisef signal age;

model sqrtpeak =signal noisef age spec

age*signal /*signal*spec noisef*spec age*spec age*noisef signal*noisef signal*age*spec*/

/*noisef*age*spec signal*noisef*spec */

/solution outp=preds ddfm=bw /*KR*/;

lsmeans signal noisef age spec age*signal /diff;

repeated /type=ar(1) subject=eagle; title 'mistuned'; run;

proc plot data=preds; plot resid*pred;

proc univariate data=preds plot normal; var resid;run;

proc sort data=preds; by signal age;

proc means data=preds; by signal age; var relpeak;

output out=azl mean=xrelpeak; run;

proc gplot; plot xrelpeak*signal=age; run;

**SAS Code For Analysis of Amplitude Modulation in Noise**

%let y=1; /* beginning number */

%let xx=27; /* ending number */

%let ne=8; /* number of eagles in the data set */

%let z1 = z:\*FOLDER*\eagles\eagle1a\noise AM\;

%let z2 = z:\*FOLDER*\eagles\eagle2\noise AM\;

%let z3 = z:\*FOLDER*\eagles\eagle3\noise AM\;

%let z4 = z:\*FOLDER*\eagles\eagle4\noise AM\;

%let z5 = z:\*FOLDER*\eagles\2018-09-06 WCV BAEA105\noise AM\;

%let z6 = z:\*FOLDER*\eagles\2019-02-09 LW GOEA107\noise AM\;

%let z7 = z:\*FOLDER*\eagles\2019-02-09 LW goea108\noise AM\;

%let z8 = z:\*FOLDER*\eagles\2019-02-09 LW BAEA107\noise AM\;

%let w = .txt;

symbol1 c=green v=dot i=join line=1 height=2 width=4;

symbol2 c=blue v=square i=join line=2 height=3 width=4;

symbol3 c=red v=z i=join line=3 width=4;

symbol4 c=black v=star i=join line=2 width=4;

symbol5 c=green v=plus i=join line=2 width=4;

symbol6 c=blue v=square i=join line=2 width=4;

%macro allthem;

%let i=%eval(0);

%do zz=1 %to &ne;

%do p=&y %to &xx;

%if &zz=1 %then %do;

%if &p=1 %then %let Q=FULLspec_baea10404_1000_100_1_;

%else %if &p=2 %then %let Q=FULLspec_baea10404_1000_100_4_;

%else %if &p=3 %then %let Q=FULLspec_baea10404_1000_100_5_;

%else %if &p=4 %then %let Q=FULLspec_baea10404_1000_400_1_;

%else %if &p=5 %then %let Q=FULLspec_baea10404_1000_400_4_;

%else %if &p=6 %then %let Q=FULLspec_baea10404_1000_400_5_;

%else %if &p=7 %then %let Q=FULLspec_baea10404_1000_700_1_;

%else %if &p=8 %then %let Q=FULLspec_baea10404_1000_700_4_;

%else %if &p=9 %then %let Q=FULLspec_baea10404_1000_700_5_;

%else %if &p=10 %then %let Q=FULLspec_baea10404_2000_100_1_;

%else %if &p=11 %then %let Q=FULLspec_baea10404_2000_100_4_;

%else %if &p=12 %then %let Q=FULLspec_baea10404_2000_100_5_;

%else %if &p=13 %then %let Q=FULLspec_baea10404_2000_400_1_;

%else %if &p=14 %then %let Q=FULLspec_baea10404_2000_400_4_;

%else %if &p=15 %then %let Q=FULLspec_baea10404_2000_400_5_;

%else %if &p=16 %then %let Q=FULLspec_baea10404_2000_700_1_;

%else %if &p=17 %then %let Q=FULLspec_baea10404_2000_700_4_;

%else %if &p=18 %then %let Q=FULLspec_baea10404_2000_700_5_;

%else %if &p=19 %then %let Q=FULLspec_baea10404_3000_100_1_;

%else %if &p=20 %then %let Q=FULLspec_baea10404_3000_100_4_;

%else %if &p=21 %then %let Q=FULLspec_baea10404_3000_100_5_;

%else %if &p=22 %then %let Q=FULLspec_baea10404_3000_400_1_;

%else %if &p=23 %then %let Q=FULLspec_baea10404_3000_400_4_;

%else %if &p=24 %then %let Q=FULLspec_baea10404_3000_400_5_;

%else %if &p=25 %then %let Q=FULLspec_baea10404_3000_700_1_;

%else %if &p=26 %then %let Q=FULLspec_baea10404_3000_700_4_;

%else %if &p=27 %then %let Q=FULLspec_baea10404_3000_700_5_;

%let R = "&Z1&Q&W"; %end;

%else %if &zz=2 %then %do;

%if &p=1 %then %let Q=FULLspec_baea20404_1000_100_1_;

%else %if &p=2 %then %let Q=FULLspec_baea20404_1000_100_4_;

%else %if &p=3 %then %let Q=FULLspec_baea20404_1000_100_5_;

%else %if &p=4 %then %let Q=FULLspec_baea20404_1000_400_1_;

%else %if &p=5 %then %let Q=FULLspec_baea20404_1000_400_4_;

%else %if &p=6 %then %let Q=FULLspec_baea20404_1000_400_5_;

%else %if &p=7 %then %let Q=FULLspec_baea20404_1000_700_1_;

%else %if &p=8 %then %let Q=FULLspec_baea20404_1000_700_4_;

%else %if &p=9 %then %let Q=FULLspec_baea20404_1000_700_5_;

%else %if &p=10 %then %let Q=FULLspec_baea20404_2000_100_1_;

%else %if &p=11 %then %let Q=FULLspec_baea20404_2000_100_4_;

%else %if &p=12 %then %let Q=FULLspec_baea20404_2000_100_5_;

%else %if &p=13 %then %let Q=FULLspec_baea20404_2000_400_1_;

%else %if &p=14 %then %let Q=FULLspec_baea20404_2000_400_4_;

%else %if &p=15 %then %let Q=FULLspec_baea20404_2000_400_5_;

%else %if &p=16 %then %let Q=FULLspec_baea20404_2000_700_1_;

%else %if &p=17 %then %let Q=FULLspec_baea20404_2000_700_4_;

%else %if &p=18 %then %let Q=FULLspec_baea20404_2000_700_5_;

%else %if &p=19 %then %let Q=FULLspec_baea20404_3000_100_1_;

%else %if &p=20 %then %let Q=FULLspec_baea20404_3000_100_4_;

%else %if &p=21 %then %let Q=FULLspec_baea20404_3000_100_5_;

%else %if &p=22 %then %let Q=FULLspec_baea20404_3000_400_1_;

%else %if &p=23 %then %let Q=FULLspec_baea20404_3000_400_4_;

%else %if &p=24 %then %let Q=FULLspec_baea20404_3000_400_5_;

%else %if &p=25 %then %let Q=FULLspec_baea20404_3000_700_1_;

%else %if &p=26 %then %let Q=FULLspec_baea20404_3000_700_4_;

%else %if &p=27 %then %let Q=FULLspec_baea20404_3000_700_5_;

%let R = "&Z2&Q&W"; %end;

%else %if &zz=3 %then %do;

%if &p=1 %then %let Q=FULLspec_baea1030509_1000_100_1_;

%else %if &p=2 %then %let Q=FULLspec_baea1030509_1000_100_4_;

%else %if &p=3 %then %let Q=FULLspec_baea1030509_1000_100_5_;

%else %if &p=4 %then %let Q=FULLspec_baea1030509_1000_400_1_;

%else %if &p=5 %then %let Q=FULLspec_baea1030509_1000_400_4_;

%else %if &p=6 %then %let Q=FULLspec_baea1030509_1000_400_5_;

%else %if &p=7 %then %let Q=FULLspec_baea1030509_1000_700_1_;

%else %if &p=8 %then %let Q=FULLspec_baea1030509_1000_700_4_;

%else %if &p=9 %then %let Q=FULLspec_baea1030509_1000_700_5_;

%else %if &p=10 %then %let Q=FULLspec_baea1030509_2000_100_1_;

%else %if &p=11 %then %let Q=FULLspec_baea1030509_2000_100_4_;

%else %if &p=12 %then %let Q=FULLspec_baea1030509_2000_100_5_;

%else %if &p=13 %then %let Q=FULLspec_baea1030509_2000_400_1_;

%else %if &p=14 %then %let Q=FULLspec_baea1030509_2000_400_4_;

%else %if &p=15 %then %let Q=FULLspec_baea1030509_2000_400_5_;

%else %if &p=16 %then %let Q=FULLspec_baea1030509_2000_700_1_;

%else %if &p=17 %then %let Q=FULLspec_baea1030509_2000_700_4_;

%else %if &p=18 %then %let Q=FULLspec_baea1030509_2000_700_5_;

%else %if &p=19 %then %let Q=FULLspec_baea1030509_3000_100_1_;

%else %if &p=20 %then %let Q=FULLspec_baea1030509_3000_100_4_;

%else %if &p=21 %then %let Q=FULLspec_baea1030509_3000_100_5_;

%else %if &p=22 %then %let Q=FULLspec_baea1030509_3000_400_1_;

%else %if &p=23 %then %let Q=FULLspec_baea1030509_3000_400_4_;

%else %if &p=24 %then %let Q=FULLspec_baea1030509_3000_400_5_;

%else %if &p=25 %then %let Q=FULLspec_baea1030509_3000_700_1_;

%else %if &p=26 %then %let Q=FULLspec_baea1030509_3000_700_4_;

%else %if &p=27 %then %let Q=FULLspec_baea1030509_3000_700_5_;

%let R = "&Z3&Q&W"; %end;

%else %if &zz=4 %then %do;

%if &p=1 %then %let Q=FULLspec_baea1040509_1000_100_1_;

%else %if &p=2 %then %let Q=FULLspec_baea1040509_1000_100_4_;

%else %if &p=3 %then %let Q=FULLspec_baea1040509_1000_100_5_;

%else %if &p=4 %then %let Q=FULLspec_baea1040509_1000_400_1_;

%else %if &p=5 %then %let Q=FULLspec_baea1040509_1000_400_4_;

%else %if &p=6 %then %let Q=FULLspec_baea1040509_1000_400_5_;

%else %if &p=7 %then %let Q=FULLspec_baea1040509_1000_700_1_;

%else %if &p=8 %then %let Q=FULLspec_baea1040509_1000_700_4_;

%else %if &p=9 %then %let Q=FULLspec_baea1040509_1000_700_5_;

%else %if &p=10 %then %let Q=FULLspec_baea1040509_2000_100_1_;

%else %if &p=11 %then %let Q=FULLspec_baea1040509_2000_100_4_;

%else %if &p=12 %then %let Q=FULLspec_baea1040509_2000_100_5_;

%else %if &p=13 %then %let Q=FULLspec_baea1040509_2000_400_1_;

%else %if &p=14 %then %let Q=FULLspec_baea1040509_2000_400_4_;

%else %if &p=15 %then %let Q=FULLspec_baea1040509_2000_400_5_;

%else %if &p=16 %then %let Q=FULLspec_baea1040509_2000_700_1_;

%else %if &p=17 %then %let Q=FULLspec_baea1040509_2000_700_4_;

%else %if &p=18 %then %let Q=FULLspec_baea1040509_2000_700_5_;

%else %if &p=19 %then %let Q=FULLspec_baea1040509_3000_100_1_;

%else %if &p=20 %then %let Q=FULLspec_baea1040509_3000_100_4_;

%else %if &p=21 %then %let Q=FULLspec_baea1040509_3000_100_5_;

%else %if &p=22 %then %let Q=FULLspec_baea1040509_3000_400_1_;

%else %if &p=23 %then %let Q=FULLspec_baea1040509_3000_400_4_;

%else %if &p=24 %then %let Q=FULLspec_baea1040509_3000_400_5_;

%else %if &p=25 %then %let Q=FULLspec_baea1040509_3000_700_1_;

%else %if &p=26 %then %let Q=FULLspec_baea1040509_3000_700_4_;

%else %if &p=27 %then %let Q=FULLspec_baea1040509_3000_700_5_;

%let R = "&Z4&Q&W"; %end;

%else %if &zz=5 %then %do;

%if &p=1 %then %let Q=FULLspec_baea1050906_1000_100_1_;

%else %if &p=2 %then %let Q=FULLspec_baea1050906_1000_100_4_;

%else %if &p=3 %then %let Q=FULLspec_baea1050906_1000_100_5_;

%else %if &p=4 %then %let Q=FULLspec_baea1050906_1000_400_1_;

%else %if &p=5 %then %let Q=FULLspec_baea1050906_1000_400_4_;

%else %if &p=6 %then %let Q=FULLspec_baea1050906_1000_400_5_;

%else %if &p=7 %then %let Q=FULLspec_baea1050906_1000_700_1_;

%else %if &p=8 %then %let Q=FULLspec_baea1050906_1000_700_4_;

%else %if &p=9 %then %let Q=FULLspec_baea1050906_1000_700_5_;

%else %if &p=10 %then %let Q=FULLspec_baea1050906_2000_100_1_;

%else %if &p=11 %then %let Q=FULLspec_baea1050906_2000_100_4_;

%else %if &p=12 %then %let Q=FULLspec_baea1050906_2000_100_5_;

%else %if &p=13 %then %let Q=FULLspec_baea1050906_2000_400_1_;

%else %if &p=14 %then %let Q=FULLspec_baea1050906_2000_400_4_;

%else %if &p=15 %then %let Q=FULLspec_baea1050906_2000_400_5_;

%else %if &p=16 %then %let Q=FULLspec_baea1050906_2000_700_1_;

%else %if &p=17 %then %let Q=FULLspec_baea1050906_2000_700_4_;

%else %if &p=18 %then %let Q=FULLspec_baea1050906_2000_700_5_;

%else %if &p=19 %then %let Q=FULLspec_baea1050906_3000_100_1_;

%else %if &p=20 %then %let Q=FULLspec_baea1050906_3000_100_4_;

%else %if &p=21 %then %let Q=FULLspec_baea1050906_3000_100_5_;

%else %if &p=22 %then %let Q=FULLspec_baea1050906_3000_400_1_;

%else %if &p=23 %then %let Q=FULLspec_baea1050906_3000_400_4_;

%else %if &p=24 %then %let Q=FULLspec_baea1050906_3000_400_5_;

%else %if &p=25 %then %let Q=FULLspec_baea1050906_3000_700_1_;

%else %if &p=26 %then %let Q=FULLspec_baea1050906_3000_700_4_;

%else %if &p=27 %then %let Q=FULLspec_baea1050906_3000_700_5_;

%let R = "&Z5&Q&W"; %end;

%else %if &zz=6 %then %do;

%if &p=1 %then %let Q=FULLspec_goea1070209_1000_100_1_;

%else %if &p=2 %then %let Q=FULLspec_goea1070209_1000_100_4_;

%else %if &p=3 %then %let Q=FULLspec_goea1070209_1000_100_5_;

%else %if &p=4 %then %let Q=FULLspec_goea1070209_1000_400_1_;

%else %if &p=5 %then %let Q=FULLspec_goea1070209_1000_400_4_;

%else %if &p=6 %then %let Q=FULLspec_goea1070209_1000_400_5_;

%else %if &p=7 %then %let Q=FULLspec_goea1070209_1000_700_1_;

%else %if &p=8 %then %let Q=FULLspec_goea1070209_1000_700_4_;

%else %if &p=9 %then %let Q=FULLspec_goea1070209_1000_700_5_;

%else %if &p=10 %then %let Q=FULLspec_goea1070209_2000_100_1_;

%else %if &p=11 %then %let Q=FULLspec_goea1070209_2000_100_4_;

%else %if &p=12 %then %let Q=FULLspec_goea1070209_2000_100_5_;

%else %if &p=13 %then %let Q=FULLspec_goea1070209_2000_400_1_;

%else %if &p=14 %then %let Q=FULLspec_goea1070209_2000_400_4_;

%else %if &p=15 %then %let Q=FULLspec_goea1070209_2000_400_5_;

%else %if &p=16 %then %let Q=FULLspec_goea1070209_2000_700_1_;

%else %if &p=17 %then %let Q=FULLspec_goea1070209_2000_700_4_;

%else %if &p=18 %then %let Q=FULLspec_goea1070209_2000_700_5_;

%else %if &p=19 %then %let Q=FULLspec_goea1070209_3000_100_1_;

%else %if &p=20 %then %let Q=FULLspec_goea1070209_3000_100_4_;

%else %if &p=21 %then %let Q=FULLspec_goea1070209_3000_100_5_;

%else %if &p=22 %then %let Q=FULLspec_goea1070209_3000_400_1_;

%else %if &p=23 %then %let Q=FULLspec_goea1070209_3000_400_4_;

%else %if &p=24 %then %let Q=FULLspec_goea1070209_3000_400_5_;

%else %if &p=25 %then %let Q=FULLspec_goea1070209_3000_700_1_;

%else %if &p=26 %then %let Q=FULLspec_goea1070209_3000_700_4_;

%else %if &p=27 %then %let Q=FULLspec_goea1070209_3000_700_5_;

%let R = "&Z6&Q&W"; %end;

%else %if &zz=7 %then %do;

%if &p=1 %then %let Q=FULLspec_goea108_AMsignal_0209_80_1000_100_1_0_;

%else %if &p=2 %then %let Q=FULLspec_goea108_AMsignal_0209_80_1000_100_4_-15_;

%else %if &p=3 %then %let Q=FULLspec_goea108_AMsignal_0209_80_1000_100_5_-8_;

%else %if &p=4 %then %let Q=FULLspec_goea108_AMsignal_0209_80_1000_400_1_0_;

%else %if &p=5 %then %let Q=FULLspec_goea108_AMsignal_0209_80_1000_400_4_-15_;

%else %if &p=6 %then %let Q=FULLspec_goea108_AMsignal_0209_80_1000_400_5_-8_;

%else %if &p=7 %then %let Q=FULLspec_goea108_AMsignal_0209_80_1000_700_1_0_;

%else %if &p=8 %then %let Q=FULLspec_goea108_AMsignal_0209_80_1000_700_4_-15_;

%else %if &p=9 %then %let Q=FULLspec_goea108_AMsignal_0209_80_1000_700_5_-8_;

%else %if &p=10 %then %let Q=FULLspec_goea108_AMsignal_0209_80_2000_100_1_0_;

%else %if &p=11 %then %let Q=FULLspec_goea108_AMsignal_0209_80_2000_100_4_-15_;

%else %if &p=12 %then %let Q=FULLspec_goea108_AMsignal_0209_80_2000_100_5_-8_;

%else %if &p=13 %then %let Q=FULLspec_goea108_AMsignal_0209_80_2000_400_1_0_;

%else %if &p=14 %then %let Q=FULLspec_goea108_AMsignal_0209_80_2000_400_4_-15_;

%else %if &p=15 %then %let Q=FULLspec_goea108_AMsignal_0209_80_2000_400_5_-8_;

%else %if &p=16 %then %let Q=FULLspec_goea108_AMsignal_0209_80_2000_700_1_0_;

%else %if &p=17 %then %let Q=FULLspec_goea108_AMsignal_0209_80_2000_700_4_-15_;

%else %if &p=18 %then %let Q=FULLspec_goea108_AMsignal_0209_80_2000_700_5_-8_;

%else %if &p=19 %then %let Q=FULLspec_goea108_AMsignal_0209_80_3000_100_1_0_;

%else %if &p=20 %then %let Q=FULLspec_goea108_AMsignal_0209_80_3000_100_4_-15_;

%else %if &p=21 %then %let Q=FULLspec_goea108_AMsignal_0209_80_3000_100_5_-8_;

%else %if &p=22 %then %let Q=FULLspec_goea108_AMsignal_0209_80_3000_400_1_0_;

%else %if &p=23 %then %let Q=FULLspec_goea108_AMsignal_0209_80_3000_400_4_-15_;

%else %if &p=24 %then %let Q=FULLspec_goea108_AMsignal_0209_80_3000_400_5_-8_;

%else %if &p=25 %then %let Q=FULLspec_goea108_AMsignal_0209_80_3000_700_1_0_;

%else %if &p=26 %then %let Q=FULLspec_goea108_AMsignal_0209_80_3000_700_4_-15_;

%else %if &p=27 %then %let Q=FULLspec_goea108_AMsignal_0209_80_3000_700_5_-8_;

%let R = "&Z7&Q&W"; %end;

%else %if &zz=8 %then %do;

%if &p=1 %then %let Q=FULLspec_baea107_AMsignal_0209_80_1000_100_1_0_;

%else %if &p=2 %then %let Q=FULLspec_baea107_AMsignal_0209_80_1000_100_4_-15_;

%else %if &p=3 %then %let Q=FULLspec_baea107_AMsignal_0209_80_1000_100_5_-8_;

%else %if &p=4 %then %let Q=FULLspec_baea107_AMsignal_0209_80_1000_400_1_0_;

%else %if &p=5 %then %let Q=FULLspec_baea107_AMsignal_0209_80_1000_400_4_-15_;

%else %if &p=6 %then %let Q=FULLspec_baea107_AMsignal_0209_80_1000_400_5_-8_;

%else %if &p=7 %then %let Q=FULLspec_baea107_AMsignal_0209_80_1000_700_1_0_;

%else %if &p=8 %then %let Q=FULLspec_baea107_AMsignal_0209_80_1000_700_4_-15_;

%else %if &p=9 %then %let Q=FULLspec_baea107_AMsignal_0209_80_1000_700_5_-8_;

%else %if &p=10 %then %let Q=FULLspec_baea107_AMsignal_0209_80_2000_100_1_0_;

%else %if &p=11 %then %let Q=FULLspec_baea107_AMsignal_0209_80_2000_100_4_-15_;

%else %if &p=12 %then %let Q=FULLspec_baea107_AMsignal_0209_80_2000_100_5_-8_;

%else %if &p=13 %then %let Q=FULLspec_baea107_AMsignal_0209_80_2000_400_1_0_;

%else %if &p=14 %then %let Q=FULLspec_baea107_AMsignal_0209_80_2000_400_4_-15_;

%else %if &p=15 %then %let Q=FULLspec_baea107_AMsignal_0209_80_2000_400_5_-8_;

%else %if &p=16 %then %let Q=FULLspec_baea107_AMsignal_0209_80_2000_700_1_0_;

%else %if &p=17 %then %let Q=FULLspec_baea107_AMsignal_0209_80_2000_700_4_-15_;

%else %if &p=18 %then %let Q=FULLspec_baea107_AMsignal_0209_80_2000_700_5_-8_;

%else %if &p=19 %then %let Q=FULLspec_baea107_AMsignal_0209_80_3000_100_1_0_;

%else %if &p=20 %then %let Q=FULLspec_baea107_AMsignal_0209_80_3000_100_4_-15_;

%else %if &p=21 %then %let Q=FULLspec_baea107_AMsignal_0209_80_3000_100_5_-8_;

%else %if &p=22 %then %let Q=FULLspec_baea107_AMsignal_0209_80_3000_400_1_0_;

%else %if &p=23 %then %let Q=FULLspec_baea107_AMsignal_0209_80_3000_400_4_-15_;

%else %if &p=24 %then %let Q=FULLspec_baea107_AMsignal_0209_80_3000_400_5_-8_;

%else %if &p=25 %then %let Q=FULLspec_baea107_AMsignal_0209_80_3000_700_1_0_;

%else %if &p=26 %then %let Q=FULLspec_baea107_AMsignal_0209_80_3000_700_4_-15_;

%else %if &p=27 %then %let Q=FULLspec_baea107_AMsignal_0209_80_3000_700_5_-8_;

%let R = "&Z8&Q&W"; %end;

filename onesec &r;

data spect (drop=r filename ) ; infile onesec ;

attrib filen length=$46; attrib filename length=$34; attrib eagle length=$7;

filen="&q";

*FULLspec_baea1040509_1000_100_1_;

hz=&zz;

if hz<3 then do;

eagle=substrn(filen,10,5); spec=substrn(filen,10,4);

carrier=1.0 * substrn(filen,20,4); AMrate=1.0 * substrn(filen,25,3);

noisef=1.0 *substrn(filen,29,1); end;

else if hz<7 then do;

eagle=substrn(filen,10,7); spec=substrn(filen,10,4);

carrier=1.0 * substrn(filen,22,4); AMrate=1.0 * substrn(filen,27,3);

noisef=1.0 *substrn(filen,31,1);end;

else do;

eagle=substrn(filen,10,7); spec=substrn(filen,10,4);

carrier=1.0 * substrn(filen,35,4); AMrate=1.0 * substrn(filen,40,3);

noisef=1.0 *substrn(filen,44,1);end;

input; input; input; input r $ r $ xmin;

input r$ r $ xmax;

input r $ r $ nx;

input r $ r $ dx;

input; input; input; input; input; input; input; input;

do i=1 to nx;

input r $ r $ r $ r $ db; freq=xmin+dx*(i-1);

output; end;

input filename $ ; put 'filename: ' filename ' ' filen; run;

%if &zz=1 %then %do; %if &p=&y %then %do; data spectra; set spect; run;%end;

%else %do; data spectra; set spectra spect; %end; %end;

%else %do; data spectra; set spectra spect; %end; %end;

%end;

%MEND allthem; %allthem;

data spectra; set spectra;

age='adult';

if eagle='baea2' then do; age='juvie'; end;

else if eagle='baea105' then do; age='juvie'; end;

else if eagle='goea107' then do; age='juvie'; end;

else if eagle='goea108' then do; age='adult'; end;

else if eagle='baea107' then do; age='adult'; end;

else put 'eagle: ' eagle; run;

data carrier; set spectra;

if (freq<(carrier+50) and freq>(carrier-50)) then signal='carrier';

else if (freq<(carrier+AMrate+50) and freq>(carrier+AMrate-50)) then signal='high';

else if (freq<(carrier-AMrate+50) and freq>(carrier-AMrate-50)) then signal='low';

else if (freq<(AMrate+50) and freq>(AMrate-50)) then signal='AMrate';

else delete;run;

proc sort data=carrier; by eagle carrier AMrate noisef signal;

proc means data=carrier noprint; by eagle carrier AMrate noisef signal; var db; id freq age;

output out=meanz max=maxdb; run;

data meanz(drop=_type_ _freq_); set meanz;run;

data noise; set spectra;

if freq<5000;

if (freq<(carrier+100) and freq>(carrier-100)) then delete;

else if (freq<(carrier+AMrate+100) and freq>(carrier+AMrate-100)) then delete;

else if (freq<(carrier-AMrate+100) and freq>(carrier+AMrate-100)) then delete;

else do; do i=1 to 6;

if (freq<(AMrate*i+100) and freq>(AMrate*i-100)) then delete; end; end;run;

proc sort ; by eagle carrier AMrate noisef ;

proc mixed data=noise; by eagle carrier AMrate noisef;

model db = freq freq*freq /*freq*freq*freq*// outp=preds; run;

proc plot data=preds; plot resid*pred;

proc univariate data=preds plot normal; var resid;run;

data preds(drop=xmin xmax nx df alpha lower upper dx); set preds;

if resid<-15 or resid>15 then delete; run;

proc gplot; by eagle carrier AMrate noisef; plot resid*freq; plot pred*freq; run;

proc sort data=preds; by spec eagle carrier AMrate noisef;

proc means noprint data=preds noprint; by spec eagle carrier AMrate noisef; var resid; id pred age;

output out=noiz uclm=uclm p90=p90 p95=p95; run;

data noiz(drop=_type_ _freq_); set noiz; run;

data final; merge noiz meanz ; by eagle carrier AMrate noisef;

relpeak=maxdb - (pred+p95); if relpeak<0 then relpeak=0; run;

proc print; run;

/* FINAL MODEL */

data amrate; set final; if signal='AMrate'; run;

proc mixed data=amrate; by signal; class spec carrier eagle AMrate noisef age;

model relpeak =amrate noisef carrier amrate*noisef amrate*carrier noisef*carrier

age*noisef*carrier age*noisef /*age*noisef*amrate amrate*noisef*carrier*/

age spec spec*amrate /*age*amrate spec*noisef spec*carrier spec*amrate*noisef */

age*carrier/*spec*age spec*amrate*carrier spec*noisef*carrier spec*age*amrate spec*age*noisef*/

/solution outp=preds ddfm=bw /*KR*/;

lsmeans age spec amrate carrier noisef amrate*noisef amrate*carrier noisef*carrier

age*noisef*carrier spec*amrate/diff;

repeated /type=ar(1) subject=eagle; title 'AMrate'; run;

proc plot data=preds; plot resid*pred;

proc univariate data=preds plot normal; var resid;run;

proc sort data=preds; by AMrate noisef;

proc means data=preds; by AMrate noisef; var relpeak;

output out=azl mean=xrelpeak; run;

proc gplot; plot xrelpeak*AMrate=noisef; run;

proc sort data=preds; by AMrate carrier;

proc means data=preds; by AMrate carrier; var relpeak;

output out=azl mean=xrelpeak; run;

proc gplot; plot xrelpeak*AMrate=carrier; run;

proc sort data=preds; by AMrate spec;

proc means data=preds; by AMrate spec; var relpeak;

output out=azl mean=xrelpeak; run;

proc gplot; plot xrelpeak*AMrate=spec; run;

proc sort data=preds; by carrier noisef age;

proc means data=preds; by carrier noisef age; var relpeak;

output out=azl mean=xrelpeak; run;

proc sort data=azl; by age;

proc gplot; by age; plot xrelpeak*carrier=noisef; run;

/* FINAL MODEL */

data amrate; set final; if signal='carrier'; run;

proc mixed data=amrate; by signal; class spec carrier eagle AMrate noisef age;

model relpeak =amrate noisef carrier age spec noisef*carrier

age*noisef amrate*carrier /*age*noisef*amrate amrate*noisef*carrier*/ amrate*noisef

age*carrier age*noisef*carrier spec*carrier

spec*amrate /*spec*amrate*noisef age*amrate spec*noisef*/

spec*amrate*carrier /*spec*age spec*noisef*carrier spec*age*amrate spec*age*noisef*/

/solution outp=preds ddfm=bw /*KR*/;

lsmeans amrate carrier noisef age spec spec*amrate*carrier age*noisef*carrier /diff;

repeated /type=ar(1) subject=eagle; title 'CARRIER tone in noise'; run;

proc sort data=preds; by carrier noisef age;

proc means data=preds noprint; by carrier noisef age; var relpeak;

output out=azl mean=xrelpeak; run;

proc sort data=azl; by age;

proc gplot; by age; plot xrelpeak*carrier=noisef; run;

proc sort data=preds; by carrier spec AMrate;

proc means data=preds noprint; by carrier spec AMrate; var relpeak;

output out=azl mean=xrelpeak; run;

proc sort data=azl; by carrier;

proc gplot; by carrier; plot xrelpeak*AMrate=spec; run;

proc sort data=preds; by AMrate noisef;

proc means data=preds noprint; by AMrate noisef; var relpeak;

output out=azl mean=xrelpeak; run;

proc gplot; plot xrelpeak*AMrate=noisef; run;

data amrate; set final; if signal='high'; run;

proc mixed data=amrate; by signal; class spec carrier eagle AMrate noisef age;

model relpeak =amrate noisef carrier age spec noisef*carrier

amrate*carrier amrate*noisef/*age*noisef age*noisef*amrate amrate*noisef*carrier */

spec*carrier

spec*amrate /*age*amratespec*noisef age*carrier age*noisef*carrier spec*amrate*noisef */

spec*amrate*carrier /*spec*age spec*age*amrate spec*age*noisef spec*noisef*carrier */

/solution outp=preds ddfm=bw /*KR*/;

lsmeans amrate carrier noisef age spec spec*amrate*carrier/diff;

repeated /type=ar(1) subject=eagle; title 'HIGH tone in noise'; run;

proc sort data=preds; by carrier spec AMrate;

proc means data=preds noprint; by carrier spec AMrate; var relpeak;

output out=azl mean=xrelpeak; run;

proc sort data=azl; by carrier;

proc gplot; by carrier; plot xrelpeak*AMrate=spec; run;

proc sort data=preds; by carrier noisef;

proc means data=preds noprint; by carrier noisef; var relpeak;

output out=azl mean=xrelpeak; run;

proc gplot; plot xrelpeak*carrier=noisef; run;

proc sort data=preds; by noisef AMrate;

proc means data=preds noprint; by noisef AMrate; var relpeak;

output out=azl mean=xrelpeak; run;

proc gplot; plot xrelpeak*AMrate=noisef; run;

data amrate; set final; if signal='low'; run;

proc mixed data=amrate; by signal; class spec carrier eagle AMrate noisef age;

model relpeak =amrate noisef carrier age spec noisef*carrier

amrate*noisef /*age*noisef amrate*carrier amrate*noisef*carrier age*noisef*amrate */

spec*carrier

age*amrate /*spec*amrate spec*noisef age*carrier spec*amrate*noisef age*noisef*carrier */

/*spec*age spec*age*amrate spec*noisef*carrier spec*amrate*carrier spec*age*noisef */

/solution outp=preds ddfm=bw /*KR*/;

lsmeans amrate carrier noisef age spec noisef*carrier spec*carrier amrate*noisef age*amrate/diff;

repeated /type=ar(1) subject=eagle; title 'LOW tone in noise'; run;

proc sort data=preds; by carrier noisef;

proc means data=preds noprint; by carrier noisef; var relpeak;

output out=azl mean=xrelpeak; run;

proc gplot; plot xrelpeak*carrier=noisef; run;

proc sort data=preds; by AMrate noisef;

proc means data=preds noprint; by AMrate noisef; var relpeak;

output out=azl mean=xrelpeak; run;

proc gplot; plot xrelpeak*AMrate=noisef; run;

proc sort data=preds; by carrier spec;

proc means data=preds noprint; by carrier spec; var relpeak;

output out=azl mean=xrelpeak; run;

proc gplot; plot xrelpeak*carrier=spec; run;

proc sort data=preds; by AMrate age;

proc means data=preds noprint; by AMrate age; var relpeak;

output out=azl mean=xrelpeak; run;

proc gplot; plot xrelpeak*AMrate=age; run;

**SAS Code For Analysis of Linear FM Sweeps**

/*1=up fast 2=down fast 3=up slow 4=down slow

noisetype: 1=none, 4=white, 5=pink */

%let x=12; /* NUMBER OF RECORDS */

%let y=1; /* beginning record */

%let ne1 = 5; * number of eagles from Virginia;

%let ne2 = 3; * number of eagles from Liberty;

%let w = .txt;

* SWEEPS ;

%let z1 = z:\*FOLDER*\eagles\eagle1a\sweep\;

%let z2 = z:\*FOLDER*\eagles\eagle2\sweep\;

%let z3 = z:\*FOLDER*\eagles\eagle3\sweep\;

%let z4 = z:\*FOLDER*\eagles\eagle4\sweep\;

%let z5 = z:\*FOLDER*\eagles\2018-09-06 WCV BAEA105\sweep\;

%let z6 = z:\*FOLDER*\eagles\2019-02-09 LW GOEA107\sweep\;

%let z7 = z:\*FOLDER*\eagles\2019-02-09 LW GOEA108\sweep\;

%let z8 = z:\*FOLDER*\eagles\2019-02-09 LW BAEA107\sweep\;

%let qq1F = baea1_PlaySweepFast_0404_;

%let qq1S = baea1_PlaySweepSlow_0404_;

%let bn1 = baea1;

%let qq2F = baea2_PlaySweepFast_0404_;

%let qq2S = baea2_PlaySweepSlow_0404_;

%let bn2 = baea2;

%let qq3F = baea103_PlaySweepFast_0509_;

%let qq3S = baea103_PlaySweepSlow_0509_;

%let bn3 = baea103;

%let qq4F = baea104_PlaySweepFast_0509_;

%let qq4S = baea104_PlaySweepSlow_0509_;

%let bn4 = baea104;

%let qq5F = baea105_PlaySweepFast_0906_;

%let qq5S = baea105_PlaySweepSlow_0906_;

%let bn5 = baea105;

%let qq6F = ;

%let qq6S = goea107_PlaySweepSlow2_0209_;

%let bn6 = goea107;

%let qq7F = goea108_PlaySweepFast2_0209_;

%let qq7S = goea108_PlaySweepSlow2_0209_;

%let bn7 = goea108;

%let qq8F = baea107_PlaySweepFast2_0209_;

%let qq8S = baea107_PlaySweepSlow2_0209_;

%let bn8 = baea107;

* SWEEPS;

%macro allthem1; * Virginia birds;

%do zz=1 %to &ne1;

%do p=&y %to &x;

%if &p=1 %then %do; %let Q=1_1_; %let zx = 30_0_; %end;

%else %if &p=2 %then %do; %let Q=1_4_; %let zx = 30_-15_; %end;

%else %if &p=3 %then %do; %let Q=1_5_; %let zx = 30_-8_; %end;

%else %if &p=4 %then %do; %let Q=2_1_; %let zx = 30_0_; %end;

%else %if &p=5 %then %do; %let Q=2_4_; %let zx = 30_-15_; %end;

%else %if &p=6 %then %do; %let Q=2_5_; %let zx = 30_-8_; %end;

%else %if &p=7 %then %do; %let Q=3_1_; %let zx = 50_0_; %end;

%else %if &p=8 %then %do; %let Q=3_4_; %let zx = 50_-15_; %end;

%else %if &p=9 %then %do; %let Q=3_5_; %let zx = 50_-8_; %end;

%else %if &p=10 %then %do; %let Q=4_1_; %let zx = 50_0_; %end;

%else %if &p=11 %then %do; %let Q=4_4_; %let zx = 50_-15_; %end;

%else %if &p=12 %then %do; %let Q=4_5_; %let zx = 50_-8_; %end;

%if &zz=1 %then %do; %let birdn=&bn1;

%if &p<7 %then %let R = "&Z1&Q&qq1F&zx&W";

%else %let R = "&Z1&Q&qq1S&zx&W"; %end;

%else %if &zz=2 %then %do; %let birdn=&bn2;

%if &p<7 %then %let R = "&Z2&qq2F&Q&zx&W";

%else %let R = "&Z2&qq2S&Q&zx&W"; %end;

%else %if &zz=3 %then %do; %let birdn=&bn3;

%if &p<7 %then %let R = "&Z3&qq3F&Q&zx&W";

%else %let R = "&Z3&qq3S&Q&zx&W"; %end;

%else %if &zz=4 %then %do; %let birdn=&bn4;

%if &p<7 %then %let R = "&Z4&qq4F&Q&zx&W";

%else %let R = "&Z4&qq4S&Q&zx&W"; %end;

%else %if &zz=5 %then %do; %let birdn=&bn5;

%if &p<7 %then %let R = "&Z5&qq5F&Q&zx&W";

%else %let R = "&Z5&qq5S&Q&zx&W"; %end;

filename onesec &r;

data db(keep=alec i time freq xmin xmax npoints dtime firsttime intensity strength spec dbsweep)

info(keep=alec filetype noisetype dbsweep eagle); attrib birdn length=$7; attrib eagle length=$7;

retain xmin xmax npoints dtime firsttime;

infile onesec missover; /* DATA FILE NAME */

alec=1; /* this is to merge data sets db and info */

birdn="&birdn"; ztype=&zz; dbsweep=80;

spec=substrn(birdn,1,4);

input; input; input;

input z $ z $ xmin;

input z $ z $ xmax;

input z $ z $ npoints;

input z $ z $ dtime;

input z $ z $ firsttime;

input z $ z $ ceiling; input z $ z $ maxcand; input;

put 'start: ' ztype birdn xmin xmax npoints dtime firsttime ceiling maxcand;

do i=1 to npoints;

input ;

input z $ z $ intensity;

input z $ z $ ncand; input;

do j=1 to ncand;

input;

input z $ z $ freq; input z $ z $ strength;

time=firsttime + i*dtime;

output db; end; end;

if ztype<3 then input eagle $5. @26 filetype 1.0 @28 noisetype 1.0 ;

else input eagle $7. @28 filetype 1.0 @30 noisetype 1.0;

output info; run;

data dbinfo; merge db info; by alec; run;

%if &zz=1 %then %do; %if &p=&y %then %do; data fm1; set dbinfo; run; %end;

%else %do; data fm1; set fm1 dbinfo; run; %end; %end;

%else %do; data fm1; set fm1 dbinfo; run; %end;%end;%end;

%MEND allthem1; %allthem1;

%macro allthem2; * Liberty birds;

%do zz=6 %to 8;

%do p=&y %to &x;

%if &p=1 %then %do; %let Q=1_1_; %let zx = 30_0_; %end;

%else %if &p=2 %then %do; %let Q=1_4_; %let zx = 30_-15_; %end;

%else %if &p=3 %then %do; %let Q=1_5_; %let zx = 30_-8_; %end;

%else %if &p=4 %then %do; %let Q=2_1_; %let zx = 30_0_; %end;

%else %if &p=5 %then %do; %let Q=2_4_; %let zx = 30_-15_; %end;

%else %if &p=6 %then %do; %let Q=2_5_; %let zx = 30_-8_; %end;

%else %if &p=7 %then %do; %let Q=3_1_; %let zx = 50_0_; %end;

%else %if &p=8 %then %do; %let Q=3_4_; %let zx = 50_-15_; %end;

%else %if &p=9 %then %do; %let Q=3_5_; %let zx = 50_-8_; %end;

%else %if &p=10 %then %do; %let Q=4_1_; %let zx = 50_0_; %end;

%else %if &p=11 %then %do; %let Q=4_4_; %let zx = 50_-15_; %end;

%else %if &p=12 %then %do; %let Q=4_5_; %let zx = 50_-8_; %end;

%do j=1 %to 2; *loud and soft sweeps;

%if &j=1 %then %let jj=0_; %else %let jj=-18_;

%if &zz=6 %then %do; %let birdn=&bn6;

%if &p<7 %then %let R = "&Z6&qq6F&Q&zx&jj&W";

%else %let R = "&Z6&qq6S&Q&zx&jj&W"; %end;

%else %if &zz=7 %then %do; %let birdn=&bn7;

%if &p<7 %then %let R = "&Z7&qq7F&Q&zx&jj&W";

%else %let R = "&Z7&qq7S&Q&zx&jj&W"; %end;

%else %if &zz=8 %then %do; %let birdn=&bn8;

%if &p<7 %then %let R = "&Z8&qq8F&Q&zx&jj&W";

%else %let R = "&Z8&qq8S&Q&zx&jj&W"; %end;

filename onesec &r;

data db(keep=alec i time freq xmin xmax npoints dtime firsttime intensity strength )

info(keep=alec filetype noisetype dbsweep eagle spec dbsweep); attrib birdn length=$7; attrib eagle length=$7;

retain xmin xmax npoints dtime firsttime;

infile onesec missover; /* DATA FILE NAME */

alec=1; /* this is to merge data sets db and info */

birdn="&birdn"; ztype=&zz;

spec=substrn(birdn,1,4);

input; input; input;

input z $ z $ xmin;

input z $ z $ xmax;

input z $ z $ npoints;

input z $ z $ dtime;

input z $ z $ firsttime;

input z $ z $ ceiling; input z $ z $ maxcand; input;

put 'start: ' birdn xmin xmax npoints dtime firsttime ceiling maxcand;

do i=1 to npoints;

input ;

input z $ z $ intensity;

input z $ z $ ncand; input;

do j=1 to ncand;

input;

input z $ z $ freq; input z $ z $ strength;

time=firsttime + i*dtime;

output db; end; end;

input eagle $7. @29 filetype 1.0 @31 noisetype 1.0 @37 zk $ ;

zkk=substrn(zk,1,1);

if zkk='_' then zkkk=substrn(zk,2,1);

else if zkk='1' then zkkk=substrn(zk,4,1);

else if zkk='8' then zkkk=substrn(zk,3,1);

if zkkk='-' then dbsweep=60; else dbsweep=80;

*put 'zk: ' eagle filetype noisetype zk zkk zkkk dbsweep;

output info; run;

data dbinfo; merge db info; by alec; run;

%if &zz=1 %then %do; %if &p=&y and &j=1 %then %do; data fm; set dbinfo; run; %end;

%else %do; data fm2; set fm2 dbinfo; run; %end; %end;

%else %do; data fm2; set fm2 dbinfo; run; %end;%end;%end; %end;

%MEND allthem2; %allthem2;

data fm; set fm1 fm2;

age='adult';

if eagle='baea1' then lead=0.23;

else if eagle='baea2' then do; lead=0.0; age='juvie'; end;

else if eagle='baea103' then lead=0.102;

else if eagle='baea104' then lead=0.086;

else if eagle='baea105' then do; lead=0.041; age='juvie'; end;

else if eagle='goea107' then do; lead=0.275; age='juvie'; end;

else if eagle='goea108' then do; lead=.; age='adult'; end;

else if eagle='baea107' then do; lead=.; age='adult'; end;

else put 'eagle: ' eagle; run;

* 1=up fast 2=down fast 3=up slow 4=down slow;

data fmnlin; set fm;

if freq>1000 and freq<6000; if time>0.0113; if strength>0;

time=time-0.0113;

if filetype=1 then pred=1000+(time)*5000/0.03;

else if filetype=2 then pred=6000-(time)*5000/0.03;

else if filetype=3 then pred=1000+(time)*5000/0.06;

else if filetype=4 then pred=6000-(time)*5000/0.06;

if freq>pred+700 or freq<pred-700 then delete;

timecat=.;

if time<0.004 then delete;

freqdiff=freq-pred;

do i=1 to 30;

if filetype<3 then do;

if time<i*0.03/30 then do; timecat=i; goto here; end; end;

else do;

if time<i*0.06/30 then do; timecat=i; goto here; end; end;

end; here: ; sqstrength=strength**2; run;

proc sort data=fmnlin; by spec eagle filetype noisetype dbsweep timecat; run;

proc means noprint data=fmnlin; by spec eagle filetype noisetype dbsweep timecat;

var strength freqdiff; id lead age;

output out=eachbird mean=xstrength xdiff; run;

proc print; run;

proc sort data=eachbird; by spec filetype noisetype dbsweep timecat; run;

proc means noprint data=eachbird; by spec filetype noisetype dbsweep timecat; var xstrength;

output out=xspec mean=xstrength; run;

symbol1 c=green v=dot i=join line=1 height=2 width=4;

symbol2 c=blue v=square i=join line=2 height=3 width=4;

symbol3 c=red v=z i=join line=3 width=4;

symbol4 c=black v=star i=none line=2 width=4;

symbol5 c=green v=plus i=none line=2 width=4;

symbol6 c=blue v=square i=join line=2 width=4;

data xspec1; set xspec; retain oldtime 0;

put timecat oldtime xstrength;

if oldtime=0 then do i=1 to timecat-1; cat=timecat; os=xstrength;

timecat=i; xstrength=0; output; timecat=cat; xstrength=os; end;

else if timecat<oldtime and timecat>3 then do i=3 to timecat-1;os=xstrength;

cat=timecat; timecat=i; xstrength=0; output; timecat=cat;xstrength=os; end;

else if timecat ne oldtime+1 then do i=oldtime+1 to timecat-1; os=xstrength;

cat=timecat; timecat=i; xstrength=0; output; timecat=cat; xstrength=os; end;

output;

oldtime=timecat; run;

axis2 order=(3 to 25 by 5);

axis1 order=(0 to 1 by 0.2);

proc sort data=xspec1; by spec filetype dbsweep;

proc gplot data=xspec1; by spec filetype dbsweep; plot xstrength*timecat=noisetype/vaxis=axis1 haxis=axis2; run;

*************************;

data zero; set eachbird;

retain oldtimecat oldfiletype oldnoisetype 0;

icat=0; xstren=.;

if (filetype ne oldfiletype) or (noisetype ne oldnoisetype) then do;

if timecat>1 then do; do i=1 to timecat-1; icat=i; xstren=0; output; end; end;

icat=0; xstren=.; output; end;

else do; icat=0; xstren=.;

if timecat ne oldtimecat+1 then do;

do i=oldtimecat+1 to timecat-1; icat=i; xstren=0; output; end; end; xstren=.; icat=0; output; end;

oldfiletype=filetype; oldnoisetype=noisetype; oldtimecat=timecat; run;

data zero1(drop=oldtimecat oldfiletype oldnoisetype icat xstren i _type_ _freq_); set zero;

if icat>0 then timecat=icat; if xstren=0 then xstrength=0; run;

data zero2; set zero1;

retain oldtimecat 27 oldfiletype 0 oldnoisetype 0;

xstren=.;

if timecat=1 and oldtimecat ne 27 then do;

do i=oldtimecat+1 to 27; xstren=0; output; end; xstren=.; end;

oldtimecat=timecat; oldfiletype=filetype; oldnoisetype=noisetype;

output;run;

data zero3(drop=oldtimecat oldfiletype oldnoisetype xstren i); set zero2;

if xstren ne . then do;

xstrength=0; timecat=i; filetype=oldfiletype; noisetype=oldnoisetype; end;run;

symbol1 c=green v=dot i=join line=1 height=2 width=4;

symbol2 c=blue v=box i=join line=2 height=3 width=4;

symbol3 c=red v=z i=join line=3 width=4;

symbol4 c=black v=star i=join line=2 width=4;

symbol5 c=green v=plus i=join line=2 width=4;

symbol6 c=blue v=square i=join line=2 width=4;

/* take out first 0.015 or 0.0075 (slow or fast) sec */

proc sort data=fmnlin; by eagle filetype noisetype time; run;

data clip; set fmnlin;

if filetype=1 or filetype=2 then do; if time>0.0075; end;

else if filetype=3 or filetype=4 then do; if time>0.015; end; run;

proc mixed data=clip; class spec eagle filetype noisetype age;

model strength = spec filetype noisetype time age filetype*time noisetype*time

filetype*noisetype*time/**/

lead lead*filetype lead*noisetype lead*time lead*time*filetype lead*time*noisetype/* */

/solution outp=preds ddfm=bw /*KR*/;

lsmeans filetype noisetype /diff;

repeated /type=ar(1) subject=eagle; title 'fm sweep CLIP/ STRENGTH'; run;

proc plot data=preds; plot resid*pred;

proc univariate data=preds plot normal; var resid;run;

data one; set clip; if filetype=1; run;

proc sort data=one; by eagle filetype noisetype time; run;

proc mixed data=one; class spec eagle noisetype age;

model strength = noisetype time spec age time*time

age*time spec*time /* age*noisetype spec*noisetype spec*noisetype*time spec*age */

noisetype*time time*time*noisetype /*spec*time*age spec*age*time*time*/

/* age*time*noisetype time*time*noisetype*age */

/solution outp=preds ddfm=bw /*KR*/;

lsmeans noisetype spec age /diff;

repeated /type=ar(1) subject=eagle; title 'fm sweep CLIP/ STRENGTH filetype=1'; run;

proc plot data=preds; plot resid*pred; plot resid*time; run;

proc univariate data=preds plot normal; var resid;run;

proc sort data=preds; by time spec ;

proc means noprint data=preds; by time spec ; var strength;

output out=azl mean=xstr; run;

proc gplot; plot xstr*time=spec; run;

proc sort data=preds; by time age ;

proc means noprint data=preds; by time age ; var strength;

output out=azl mean=xstr; run;

proc gplot; plot xstr*time=age; run;

proc sort data=preds; by time noisetype ;

proc means noprint data=preds; by time noisetype ; var strength;

output out=azl mean=xstr; run;

proc gplot; plot xstr*time=noisetype; run;

* complete model for effect sizes;

proc mixed data=one; class eagle noisetype ;

model strength = noisetype time /*noisetype*time*/

lead lead*noisetype /*lead*time*noisetype*/ lead*time

/solution outp=preds ddfm=bw /*KR*/;

lsmeans noisetype /diff;

repeated /type=ar(1) subject=eagle; title 'fm sweep CLIP/ STRENGTH filetype=1'; run;

proc plot data=preds; plot resid*pred; plot resid*time; run;

proc univariate data=preds plot normal; var resid;run;

data two; set clip; if filetype=2; run;

proc sort data=two; by eagle filetype noisetype time; run;

proc mixed data=two; class spec eagle noisetype age;

model strength = noisetype time spec age

age*time spec*time /*age*noisetype time*time spec*noisetype*time */

/*spec*noisetype time*time*noisetype */ noisetype*time

/*spec*age spec*age*time*time spec*time*age */

/*age*time*noisetype time*time*noisetype*age*/

/solution outp=preds ddfm=bw /*KR*/;

lsmeans noisetype spec age /diff;

repeated /type=ar(1) subject=eagle; title 'fm sweep CLIP/ STRENGTH filetype=2'; run;

proc plot data=preds; plot resid*pred;

proc univariate data=preds plot normal; var resid;run;

proc sort data=preds; by time spec ;

proc means noprint data=preds; by time spec ; var strength;

output out=azl mean=xstr; run;

proc gplot; plot xstr*time=spec; run;

proc sort data=preds; by time age ;

proc means noprint data=preds; by time age ; var strength;

output out=azl mean=xstr; run;

proc gplot; plot xstr*time=age; run;

proc sort data=preds; by time noisetype ;

proc means noprint data=preds; by time noisetype ; var strength;

output out=azl mean=xstr; run;

proc gplot; plot xstr*time=noisetype; run;

data thr; set clip; if filetype=3; run;

proc sort data=thr; by eagle filetype noisetype time ; run;

proc mixed data=thr; class spec eagle noisetype age;

model strength = noisetype time time*time spec age

age*time spec*time /*age*noisetype spec*noisetype*time*/

/*spec*age spec*noisetype time*time*noisetype noisetype*time spec*time*age*/

time*time*spec time*time*age/**/

/*spec*age*time*time age*time*noisetype time*time*noisetype*age*/

/solution outp=preds ddfm=bw /*KR*/;

lsmeans noisetype age spec /diff;

repeated /type=ar(1) subject=eagle; title 'fm sweep CLIP/ STRENGTH filetype=3'; run;

proc plot data=preds; plot resid*pred; plot resid*time; run;

proc univariate data=preds plot normal; var resid;run;

proc sort data=preds; by time spec ;

proc means noprint data=preds; by time spec ; var strength;

output out=azl mean=xstr; run;

proc gplot; plot xstr*time=spec; run;

proc sort data=preds; by time age ;

proc means noprint data=preds; by time age ; var strength;

output out=azl mean=xstr; run;

proc gplot; plot xstr*time=age; run;

* complete model for effect sizes;

data thr; set clip; if filetype=3; run;

proc sort data=thr; by eagle filetype noisetype time; run;

proc mixed data=thr; class eagle noisetype ;

model strength = noisetype time time*time /*time*time*lead noisetype*time*/

lead lead*noisetype /*lead*time*noisetype */ lead*time

/solution outp=preds ddfm=bw /*KR*/;

lsmeans noisetype /diff;

repeated /type=ar(1) subject=eagle; title 'fm sweep CLIP/ STRENGTH filetype=3'; run;

data fo; set clip; if filetype=4; run;

proc sort data=fo; by eagle filetype noisetype time; run;

proc mixed data=fo; class spec eagle noisetype age;

model strength = noisetype time time*time spec age

spec*time /*age*noisetype age*time spec*noisetype*time*/

spec*age time*time*noisetype noisetype*time /*spec*noisetype spec*age*time*time spec*time*age*/

time*time*spec

/* age*time*noisetype time*time*noisetype*age time*time*age*/

/solution outp=preds ddfm=bw /*KR*/;

lsmeans noisetype spec age /diff;

repeated /type=ar(1) subject=eagle;

title 'fm sweep CLIP/ STRENGTH filetype=4'; run;

proc plot data=preds; plot resid*pred;

proc univariate data=preds plot normal; var resid;run;

proc sort data=preds; by time spec ; run;

proc means data=preds noprint; by time spec; var strength;

output out=azl mean=xstr; run;

proc gplot; plot xstr*time=spec; run;

proc sort data=preds; by time noisetype ; run;

proc means data=preds noprint; by time noisetype; var strength;

output out=azl mean=xstr; run;

proc gplot; plot xstr*time=noisetype; run;

proc sort data=preds; by spec age ; run;

proc means data=preds noprint; by spec age; var strength;

output out=azl mean=xstr; run;

proc gplot; plot xstr*age=spec; run;

**SAS Code For Analysis of Sinusoidal FM Sweeps**

/*filetype: 1/3 = 70 Hz

2/4 = 110 Hz

depth: 1/2 = 400 (800) depth

3/4 = 700 (1400) depth

noisetype: 1=none, 4=white, 5=pink */

%let x=12; /* NUMBER OF RECORDS */

%let y=1; /* beginning record */

%let ne=8; /* number of eagles */

%let w = .txt;

* sinusoidal FM;

%let qq1 = baea1_PlayFM_0404__75;

%let qq2 = baea2_PlayFM_0404__75;

%let qq3 = baea103_PlayFM_0509__75;

%let qq4 = baea104_PlayFM_0509__75;

%let qq5 = baea105_PlayFM_0906__75;

%let qq6 = goea107_PlayFM_0209__75;

%let qq7 = goea108_PlayFM_0209__75;

%let qq8 = baea107_PlayFM_0209__75;

%let z1 = z:\*FOLDER*\eagles\eagle1a\fm\;

%let z2 = z:\*FOLDER*\eagles\eagle2\fm\;

%let z3 = z:\*FOLDER*\eagles\eagle3\fm\;

%let z4 = z:\*FOLDER*\eagles\eagle4\fm\;

%let z5 = z:\*FOLDER*\eagles\2018-09-06 WCV BAEA105\fm\;

%let z6 = z:\*FOLDER*\eagles\2019-02-09 LW GOEA107\fm\;

%let z7 = z:\*FOLDER*\eagles\2019-02-09 LW GOEA108\fm\;

%let z8 = z:\*FOLDER*\eagles\2019-02-09 LW BAEA107\fm\;

%macro allthem;

%do zx=1 %to &ne;

%do p=&y %to &x;

* SINUSOIDAL FM;

%if &p=1 %then %do; %let Q=1_1_; %let zz = 0_; %end;

%else %if &p=2 %then %do; %let Q=1_4_; %let zz = -15_; %end;

%else %if &p=3 %then %do; %let Q=1_5_; %let zz = -8_; %end;

%else %if &p=4 %then %do; %let Q=2_1_; %let zz = 0_; %end;

%else %if &p=5 %then %do; %let Q=2_4_; %let zz = -15_; %end;

%else %if &p=6 %then %do; %let Q=2_5_; %let zz = -8_; %end;

%else %if &p=7 %then %do; %let Q=3_1_; %let zz = 0_; %end;

%else %if &p=8 %then %do; %let Q=3_4_; %let zz = -15_; %end;

%else %if &p=9 %then %do; %let Q=3_5_; %let zz = -8_; %end;

%else %if &p=10 %then %do; %let Q=4_1_; %let zz = 0_; %end;

%else %if &p=11 %then %do; %let Q=4_4_; %let zz = -15_; %end;

%else %if &p=12 %then %do; %let Q=4_5_; %let zz = -8_; %end;

%if &zx=1 %then %let R = "&Z1&Q&qq1&zz&W";

%else %if &zx=2 %then %let R = "&Z2&Q&qq2&zz&W";

%else %if &zx=3 %then %let R = "&Z3&Q&qq3&zz&W";

%else %if &zx=4 %then %let R = "&Z4&Q&qq4&zz&W";

%else %if &zx=5 %then %let R = "&Z5&Q&qq5&zz&W";

%else %if &zx=6 %then %let R = "&Z6&Q&qq6&zz&W";

%else %if &zx=7 %then %let R = "&Z7&Q&qq7&zz&W";

%else %if &zx=8 %then %let R = "&Z8&Q&qq8&zz&W";

%put &zx &p;

%put &z1 ' ' &q ' ' &qq1 ' ' &zz ' ' &w;

filename onesec &r;

data db(keep=alec i time freq xmin xmax npoints dtime firsttime intensity strength filenam)

info(keep=alec filetype noisetype eagle);

retain xmin xmax npoints dtime firsttime;

infile onesec missover; /* DATA FILE NAME */

alec=1; /* this is to merge data sets db and info */

filenam=&r;

input; input; input;

input z $ z $ xmin;

input z $ z $ xmax;

input z $ z $ npoints;

input z $ z $ dtime;

input z $ z $ firsttime;

input z $ z $ ceiling; input z $ z $ maxcand; input;

* put 'start: ' xmin xmax npoints dtime firsttime ceiling maxcand;

do i=1 to npoints;

input ;

input z $ z $ intensity;

input z $ z $ ncand; input;

do j=1 to ncand;

input;

input z $ z $ freq; input z $ z $ strength;

time=firsttime + i*dtime;

output db; end;end;

input @1 filetype 1.0 @3 noisetype 1.0 @5 eagle $7.; spec=substrn(eagle,1,4);

output info; run;

data dbinfo; merge db info; by alec; run;

%if &zx=1 %then %do;

%if &p=&y %then %do; data fm; set dbinfo; run; %end;

%else %do; data fm; set fm dbinfo; run; %end; %end;

%else %do; data fm; set fm dbinfo; run; %end;%end; %end;

%MEND allthem; %allthem;

data fm; set fm;

spec=substrn(eagle,1,4);

age='adult';

if eagle='baea1_P' then lead=0.23;

else if eagle='baea2_P' then do; lead=0.0; age='juvie'; end;

else if eagle='baea103' then lead=0.102;

else if eagle='baea104' then lead=0.086;

else if eagle='baea105' then do; lead=0.041; age='juvie'; end;

else if eagle='goea107' then do; lead=0.275; age='juvie'; end;

else if eagle='goea108' then do; lead=.; age='adult'; end;

else if eagle='baea107' then do; lead=.; age='adult'; end;

else put 'eagle: ' eagle; run;

proc sort data=fm; by spec filenam eagle;

proc means data=fm noprint; by spec filenam eagle; var strength;

output out=xz n=n; run;

proc print; run;

symbol1 c=black v=dot i=join line=1 height=2 width=4;

symbol2 c=blue v=N i=join line=2 height=2 width=4;

symbol3 c=green v=W i=join line=3 height=2 width=4;

symbol4 c=red v=P i=join line=2 height=2 width=3;

symbol5 c=green v=plus i=join line=2;

symbol6 c=blue v=square i=join line=2;

/* sound starts at 0.0113 sec */

/* get rid of beginning of file -- say 0.015 sec */

/* use mod() to fold cycles on top of each other -- turn time into reltime */

/* sin=predicted frequency of stimulus as a function of reltime -- call this noisetype=0 */

data fmnlin; set fm; pi=3.14159265;

if filetype=1 or filetype=3 then rate=70; else rate= 110;

if filetype=1 or filetype=2 then depth=400; else depth=700;

if freq<3000; if time>0.015;

if rate=110 then time=time-0.01225; else time=time-0.0121;

/*if rate=110 then time=time-0.0017; else if depth=200 then time=time-0.009; else time=time-0.0095;*/

if strength>0;

if rate=110 then period=0.00909; else period=0.014286;

reltime=mod(time,period); if reltime <(period/2) then direct='up'; else direct='dn';

timecata=20*reltime/period; timecat=ceil(timecata);

sin=2000 + depth*sin(2*pi*(1/period)*reltime /*- pi/2*/);

freqdiff=freq-sin; output;

noisetype=0; freq=sin; strength=.;freqdiff=.; output; run;

symbol1 c=black v=dot i=join line=1 height=2 width=4;

symbol2 c=blue v=N i=join line=2 height=2 width=4;

symbol3 c=green v=W i=join line=3 height=2 width=4;

symbol4 c=red v=P i=join line=2 height=2 width=4;

symbol5 c=green v=plus i=join line=2;

symbol6 c=blue v=square i=join line=2;

* THIS IS FOR THE FOLDED CYCLES -- AVERAGE ACROSS CYCLES (timecat based on reltime) FOR 1 MEAN CYCLE *;

proc sort data=fmnlin; by spec filetype noisetype timecat; run;

proc means data=fmnlin noprint; by spec filetype noisetype timecat; var strength freq freqdiff;

id sin reltime lead;

output out=timecat mean=xstrength xfreq xdiff stderr=SEstrength SEfreq sediff; run;

data timecat(drop=_type_ _freq_); set timecat; run;

proc print; run;

proc sort ; by spec filetype reltime;

proc gplot; by spec filetype; plot xfreq*reltime=noisetype; plot xdiff*reltime=noisetype; run;

proc gplot; by spec filetype; plot xfreq*timecat=noisetype; plot xdiff*timecat=noisetype; run;

* THIS IS FOR THE FOLDED CYCLES -- AVERAGE ACROSS CYCLES (timecat based on reltime) FOR 1 MEAN CYCLE *;

proc sort data=fmnlin; by spec eagle filetype noisetype timecat; run;

proc means data=fmnlin noprint; by spec eagle filetype noisetype timecat;

var strength freq freqdiff; id age sin reltime lead;

output out=eagtimecat mean=xstrength xfreq xdiff stderr=SEstrength SEfreq sediff; run;

data eagtimecat(drop=_type_); set eagtimecat; if noisetype>0; if filetype>0; sqrtfreq=xfreq**0.25; run;

proc sort data=eagtimecat; by filetype; run;

proc mixed data=eagtimecat; *by filetype; class spec eagle filetype noisetype timecat age;

model xfreq = filetype noisetype timecat age spec spec*filetype spec*timecat spec*age

age*timecat

/* spec*filetype*timecat */ age*filetype age*filetype*noisetype age*noisetype

age*noisetype*timecat age*filetype*timecat/**/

filetype*timecat spec*noisetype timecat*noisetype /*filetype*noisetype filetype*noisetype*timecat*/

/solution outp=preds ddfm=bw /*KR*/;

*lsmeans filetype noisetype filetype*timecat /diff;

repeated /type=ar(1) subject=eagle; title 'mean frequency'; run;

proc plot data=preds; plot resid*pred;

proc univariate data=preds plot normal; var resid;run;

proc sort data=eagtimecat; by filetype; run;

proc mixed data=eagtimecat; *by filetype; class eagle filetype noisetype timecat;

model xdiff = filetype noisetype timecat lead

filetype*timecat /*timecat*noisetype filetype*noisetype filetype*noisetype*timecat */

lead*timecat /*lead*noisetype lead*timecat*noisetype*/

/solution outp=preds ddfm=bw /*KR*/;

lsmeans filetype noisetype filetype*timecat /diff;

repeated /type=ar(1) subject=eagle; title 'tone in noise'; run;

proc plot data=preds; plot resid*pred;

proc univariate data=preds plot normal; var resid;run;

proc sort data=eagtimecat; by filetype; run;

proc mixed data=eagtimecat; *by filetype; class spec eagle filetype noisetype timecat age;

model xstrength = filetype noisetype timecat age spec spec*timecat spec*age

age*timecat age*filetype

age*filetype*timecat/*spec*noisetype spec*filetype age*noisetype age*noisetype*timecat age*filetype*noisetype*/

filetype*timecat filetype*noisetype

/*lead(age) timecat*noisetype spec*filetype*timecat filetype*noisetype*timecat*/

/*timecat*lead(age) noisetype*lead(age) timecat*noisetype*lead(age)*/

/solution outp=preds ddfm=bw /*KR*/;

*lsmeans filetype noisetype filetype*timecat /diff;

repeated /type=ar(1) subject=eagle; title 'mean frequency'; run;

proc plot data=preds; plot resid*pred;

proc univariate data=preds plot normal; var resid;run;

/* FINAL MODELS */

/* RUN BY FILETYPE */

data eagone; set eagtimecat; if filetype=1; run;

proc mixed data=eagone; class spec eagle noisetype timecat;

model xfreq = noisetype timecat spec timecat*spec

/*noisetype*spec timecat*noisetype noisetype*timecat*spec*/

/solution outp=preds ddfm=bw /*KR*/;

lsmeans noisetype timecat spec timecat*spec/*/diff*/;

repeated /type=ar(1) subject=eagle; title 'sinusoid FM filetype=1'; run;

proc plot data=preds; plot resid*pred;

proc univariate data=preds plot normal; var resid;run;

proc sort data=preds; by noisetype timecat;

proc gplot data=preds; by noisetype; plot pred*timecat=spec; run;

data eagtwo; set eagtimecat; if filetype=2; run;

proc mixed data=eagtwo; class eagle noisetype timecat spec;

model xfreq = noisetype timecat spec timecat*spec

/*noisetype*spec timecat*noisetype noisetype*timecat*spec*/ /* */

/solution outp=preds ddfm=bw /*KR*/;

lsmeans noisetype timecat spec timecat*spec /*/diff*/;

repeated /type=ar(1) subject=eagle; title 'sinusoid FM filetype=2'; run;

proc plot data=preds; plot resid*pred;

proc univariate data=preds plot normal; var resid;run;

proc sort data=preds; by noisetype timecat;

proc gplot data=preds; by noisetype; plot pred*timecat=spec; run;

data eagthr; set eagtimecat; if filetype=3; run;

proc mixed data=eagthr; class eagle noisetype timecat spec;

model xfreq = noisetype timecat spec timecat*spec

/*noisetype*spec*/ /*timecat*noisetype noisetype*timecat*spec */

/solution outp=preds ddfm=bw /*KR*/;

lsmeans noisetype timecat spec timecat*spec /*/diff*/;

repeated /type=ar(1) subject=eagle; title 'sinusoid FM filetype=3'; run;

proc plot data=preds; plot resid*pred;

proc univariate data=preds plot normal; var resid;run;

proc sort data=preds; by noisetype timecat;

proc gplot data=preds; by noisetype; plot pred*timecat=spec; run;

data eagfr; set eagtimecat; if filetype=4; run;

proc mixed data=eagfr; class eagle noisetype timecat spec;

model xfreq = noisetype timecat spec timecat*spec

/*noisetype*spec*/ /*timecat*noisetype noisetype*timecat*spec */

/solution outp=preds ddfm=bw /*KR*/;

lsmeans noisetype timecat spec timecat*spec /*/diff*/;

repeated /type=ar(1) subject=eagle; title 'sinusoid FM filetype=4'; run;

proc plot data=preds; plot resid*pred;

proc univariate data=preds plot normal; var resid;run;

proc sort data=preds; by noisetype timecat;

proc gplot data=preds; by noisetype; plot pred*timecat=spec; run;

/* RUN BY FILETYPE */

data eagone; set eagtimecat; if filetype=1; run;

proc mixed data=eagone; class eagle noisetype timecat spec;

model xstrength = noisetype timecat spec /*timecat*noisetype */

timecat*spec /*noisetype*spec noisetype*timecat*spec */

/solution outp=preds ddfm=bw /*KR*/;

lsmeans noisetype timecat spec timecat*spec /*/diff*/;

repeated /type=ar(1) subject=eagle; title 'sinusoid FM STRENGTH filetype=1'; run;

proc plot data=preds; plot resid*pred;

proc univariate data=preds plot normal; var resid;run;

proc sort; by spec noisetype filetype timecat ;

proc print; var spec noisetype filetype timecat xfreq xstrength xdiff; run;

proc sort data=preds; by noisetype timecat;

proc gplot data=preds; by noisetype; plot pred*timecat=spec; run;

axis1 order=(0.0 to 20.0 by 2);

axis2 order=(0.30 to 0.80 by 0.10);

proc sort data=preds; by spec timecat;

proc means noprint data=preds; by spec timecat; var xstrength;

output out=azl mean=xstr; run;

proc print; run;

proc gplot data=azl; plot xstr*timecat=spec/vaxis=axis2 haxis=axis1; run;

data eagtwo; set eagtimecat; if filetype=2; run;

proc mixed data=eagtwo; class eagle noisetype timecat spec;

model xstrength = noisetype timecat spec /*timecat*noisetype */

timecat*spec /* noisetype*spec noisetype*spec*timecat */

/solution outp=preds ddfm=bw /*KR*/;

lsmeans noisetype timecat spec timecat*spec /* /diff */;

repeated /type=ar(1) subject=eagle; title 'sinusoid FM STRENGTH filetype=2'; run;

proc plot data=preds; plot resid*pred;

proc univariate data=preds plot normal; var resid;run;

proc sort data=preds; by noisetype timecat;

proc gplot data=preds; by noisetype; plot pred*timecat=spec; run;

axis1 order=(0.0 to 20.0 by 2);

axis2 order=(0.30 to 0.80 by 0.10);

proc sort data=preds; by spec timecat;

proc means noprint data=preds; by spec timecat; var xstrength;

output out=azl mean=xstr; run;

proc print; run;

proc gplot data=azl; plot xstr*timecat=spec/vaxis=axis2 haxis=axis1; run;

data eagthr; set eagtimecat; if filetype=3; run;

proc mixed data=eagthr; class eagle noisetype timecat spec;

model xstrength = noisetype timecat spec timecat*spec

/* noisetype*spec timecat*noisetype noisetype*timecat*spec */

/solution outp=preds ddfm=bw /*KR*/;

lsmeans noisetype timecat spec timecat*spec /*/diff*/;

repeated /type=ar(1) subject=eagle; title 'sinusoid FM STRENGTH filetype=3'; run;

proc plot data=preds; plot resid*pred;

proc univariate data=preds plot normal; var resid;run;

proc sort data=preds; by noisetype timecat;

proc gplot data=preds; by noisetype; plot pred*timecat=spec; run;

axis1 order=(0.0 to 20.0 by 2);

axis2 order=(0.30 to 0.80 by 0.10);

proc sort data=preds; by spec timecat;

proc means noprint data=preds; by spec timecat; var xstrength;

output out=azl mean=xstr; run;

proc print; run;

proc gplot data=azl; plot xstr*timecat=spec/vaxis=axis2 haxis=axis1; run;

data eagfo; set eagtimecat; if filetype=4; run;

proc mixed data=eagfo; class eagle noisetype timecat spec;

model xstrength = noisetype timecat spec timecat*spec

/*spec*noisetype timecat*noisetype spec*noisetype*timecat */

/solution outp=preds ddfm=bw /*KR*/;

lsmeans noisetype timecat spec timecat*spec /*/diff*/;

repeated /type=ar(1) subject=eagle; title 'sinusoid FM STRENGTH filetype=4'; run;

proc plot data=preds; plot resid*pred;

proc univariate data=preds plot normal; var resid;run;

proc sort data=preds; by noisetype timecat;

proc gplot data=preds; by noisetype; plot pred*timecat=spec; run;

axis1 order=(0.0 to 20.0 by 2);

axis2 order=(0.30 to 0.80 by 0.10);

proc sort data=preds; by spec timecat;

proc means noprint data=preds; by spec timecat; var xstrength;

output out=azl mean=xstr; run;

proc print; run;

proc gplot data=azl; plot xstr*timecat=spec/vaxis=axis2 haxis=axis1; run;

proc sort data=eagtimecat; by filetype; run;

proc mixed data=eagtimecat; *by filetype; class eagle filetype noisetype timecat;

model xdiff = filetype noisetype timecat lead

filetype*timecat /*timecat*noisetype filetype*noisetype filetype*noisetype*timecat */

lead*timecat /*lead*noisetype lead*timecat*noisetype*/

/solution outp=preds ddfm=bw /*KR*/;

lsmeans filetype noisetype filetype*timecat /diff;

repeated /type=ar(1) subject=eagle; title 'tone in noise'; run;

proc plot data=preds; plot resid*pred;

proc univariate data=preds plot normal; var resid;run;

/* XDIFF */

data eagone; set eagtimecat; if filetype=1; run;

proc sort data=eagone; by filetype; run;

proc mixed data=eagone; *by filetype; class eagle noisetype timecat spec;

model xdiff = noisetype timecat spec spec*timecat

/*spec*noisetype timecat*noisetype spec*timecat*noisetype */

/solution outp=preds ddfm=bw /*KR*/;

lsmeans noisetype timecat spec /*/diff*/;

repeated /type=ar(1) subject=eagle; title 'XDIFF: tone in noise FILETYPE=1'; run;

proc plot data=preds; plot resid*pred;

proc univariate data=preds plot normal; var resid;run;

proc sort data=preds; by noisetype timecat;

proc gplot data=preds; by noisetype; plot pred*timecat=spec; run;

data eagtwo; set eagtimecat; if filetype=2; run;

proc sort data=eagtwo; by filetype; run;

proc mixed data=eagtwo; *by filetype; class eagle noisetype timecat spec;

model xdiff = noisetype timecat spec spec*timecat

/* spec*noisetype timecat*noisetype spec*timecat*noisetype*/

/solution outp=preds ddfm=bw /*KR*/;

lsmeans noisetype timecat spec spec*timecat /*/diff*/;

repeated /type=ar(1) subject=eagle; title 'XDIFF: tone in noise FILETYPE=2'; run;

proc plot data=preds; plot resid*pred;

proc univariate data=preds plot normal; var resid;run;

proc sort data=preds; by noisetype timecat;

proc gplot data=preds; by noisetype; plot pred*timecat=spec; run;

data eagthr; set eagtimecat; if filetype=3; run;

proc sort data=eagthr; by filetype; run;

proc mixed data=eagthr; *by filetype; class eagle noisetype timecat spec;

model xdiff = noisetype timecat spec spec*timecat

/* spec*noisetype timecat*noisetype spec*timecat*noisetype*/

/solution outp=preds ddfm=bw /*KR*/;

lsmeans noisetype timecat spec spec*timecat /*/diff*/;

repeated /type=ar(1) subject=eagle; title 'XDIFF: tone in noise FILETYPE=3'; run;

proc plot data=preds; plot resid*pred;

proc univariate data=preds plot normal; var resid;run;

proc sort data=preds; by noisetype timecat;

proc gplot data=preds; by noisetype; plot pred*timecat=spec; run;

data eagf; set eagtimecat; if filetype=4; run;

proc sort data=eagf; by filetype; run;

proc mixed data=eagf; *by filetype; class eagle noisetype timecat spec;

model xdiff = noisetype timecat spec spec*timecat

/*spec*noisetype timecat*noisetype spec*timecat*noisetype*/

/solution outp=preds ddfm=bw /*KR*/;

lsmeans noisetype timecat spec spec*timecat /*/diff*/;

repeated /type=ar(1) subject=eagle; title 'XDIFF: tone in noise FILETYPE=4'; run;

proc plot data=preds; plot resid*pred;

proc univariate data=preds plot normal; var resid;run;

proc sort data=preds; by noisetype timecat;

proc gplot data=preds; by noisetype; plot pred*timecat=spec; run;

**Appendix 6.** Results for Amplitude Modulated (AM) stimulus high and low sidebands

### **AM High sideband**

Overall, noise had a strong effect on processing of all high sidebands (no-noise: -77.82 ± 0.75 dBV; white: -80.83 ± 0.75; pink: -81.58 ± 0.75). Phase-locking strength of the high sideband showed significant carrier × noise type (F_4, 28_ = 3.74, P = 0.015), carrier × AM rate type (F_4, 28_ = 9.74, P < 0.0001), and AM rate × noise type interactions (F_4, 28_ = 4.89, P = 0.0041).

There was also a significant species × carrier × AM rate interaction (F_4, 24_ = 7.29, P = 0.0005), as well as a significant species × AM rate interaction (F_2, 12_ = 5.41, P = 0.021). Overall, bald eagles performed better than golden eagles at phase-locking to high sidebands for 2000 and 0.3 kHz carriers when the AM rate was 400 (2000 Hz bald: -78.76 ± 1.42 dBV, golden: -84.76 ± 2.40; 0.3 kHz bald: -82.08 ± 1.44, golden: -85.40 ± 2.42) or 700 Hz (2000 Hz bald: -77.50 ± 1.47 dBV, golden: -83.33 ± 2.43; 0.3 kHz bald: -79.16 ± 1.50, golden: -82.32 ± 2.52). However, there was no significant difference between the species at 100 Hz AM rate (2000 Hz bald: -75.87 ± 1.47 dBV, golden: -76.61 ± 2.43; 0.3 kHz bald: -77.64 ± 1.47, golden: -78.63 ± 2.43). For 0.1 kHz carrier stimuli, bald eagles had stronger phase-locking for 100 Hz AM rate (bald: -79.83 ± 1.50 dBV; golden: -81.78 ± 2.52) and 700 Hz (bald: -78.45 ± 1.47 dBV; golden: -86.13 ± 2.43), but golden eagles were far better than bald eagles for the 400 Hz AM rate (bald: -80.80 ± 1.44 dBV, golden: -72.24 ± 2.42).

### **AM Low sideband**

Phase-locking to the low sideband was significantly affected by three main effects: AM rate (F_2,12_ = 4.99, P = 0.0265), noise background F_2,14_ = 39.43, P < 0.0001), and carrier tone (F_2,12_ = 12.98, P = 0.0010). Four interaction terms were also significant: carrier tone × noise (F_4,28_ = 4.98, P = 0.0037), AM rate × noise (F_4,28_ = 5.33, P = 0.0026), species × carrier tone (F_2,12_ = 8.67, P = 0.0047), and AM rate × age (F_2,12_ = 7.17, P = 0.0089). Notably, phase-locking of the low sidebands was particularly poor for stimuli with a 0.1 kHz carrier (-84.43 ± 1.06 dBV) compared to 2000 (-78.28 ± 0.99 dBV) and 0.3 kHz (-79.57 ± 1.06 dBV). In addition, both types of background noise decreased phase-locking strength to the low sideband (no-noise: -78.72 ± 0.75 dBV; white: -35%, -81.51 ± 0.75; pink: -41%, -82.05 ± 0.75), especially for the 0.1 kHz carrier stimulus.

Golden eagles generally had higher phase-locking strength compared to bald eagles for stimuli with a 0.2 kHz carrier (bald: -80.17 ± 1.01 dBV; golden: -76.38 ± 1.69), but bald eagles had stronger phase-locking at 1000 (bald: -83.22 ± 1.08 dBV; golden: -85.64 ± 1.79) and 0.3 kHz (bald: -76.98 ± 1.08 dBV; golden: -82.16 ± 1.79).
