# Supplementary material for: Selecting auditory alerting stimuli for eagles on the basis of auditory evoked potentials
Source: Conserv Physiol. 2022 Sep 16;10(1):coac059. doi: 10.1093/conphys/coac059 (PMC9486983; doi:10.1093/conphys/coac059)
Supplement: Web_Material_coac059 [file web_material_coac059.zip › Goller et al.Appx 1.Background sound intensity.docx]

**Appendix 1.** Background sound intensity profiles in the anechoic chamber


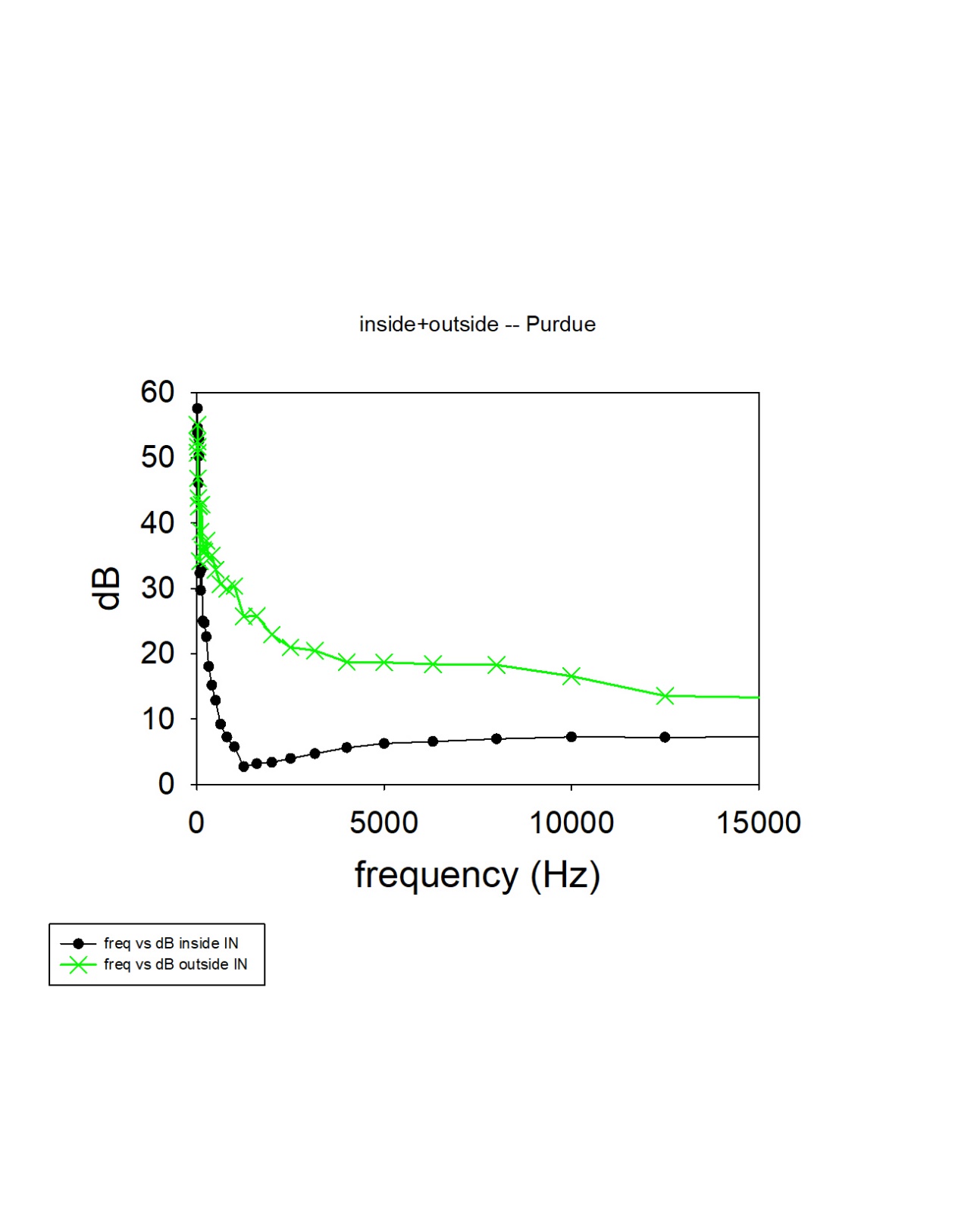


Figure A1. Background sound intensities in a laboratory setting at Purdue University both inside (black) and outside (green) of the anechoic chamber:


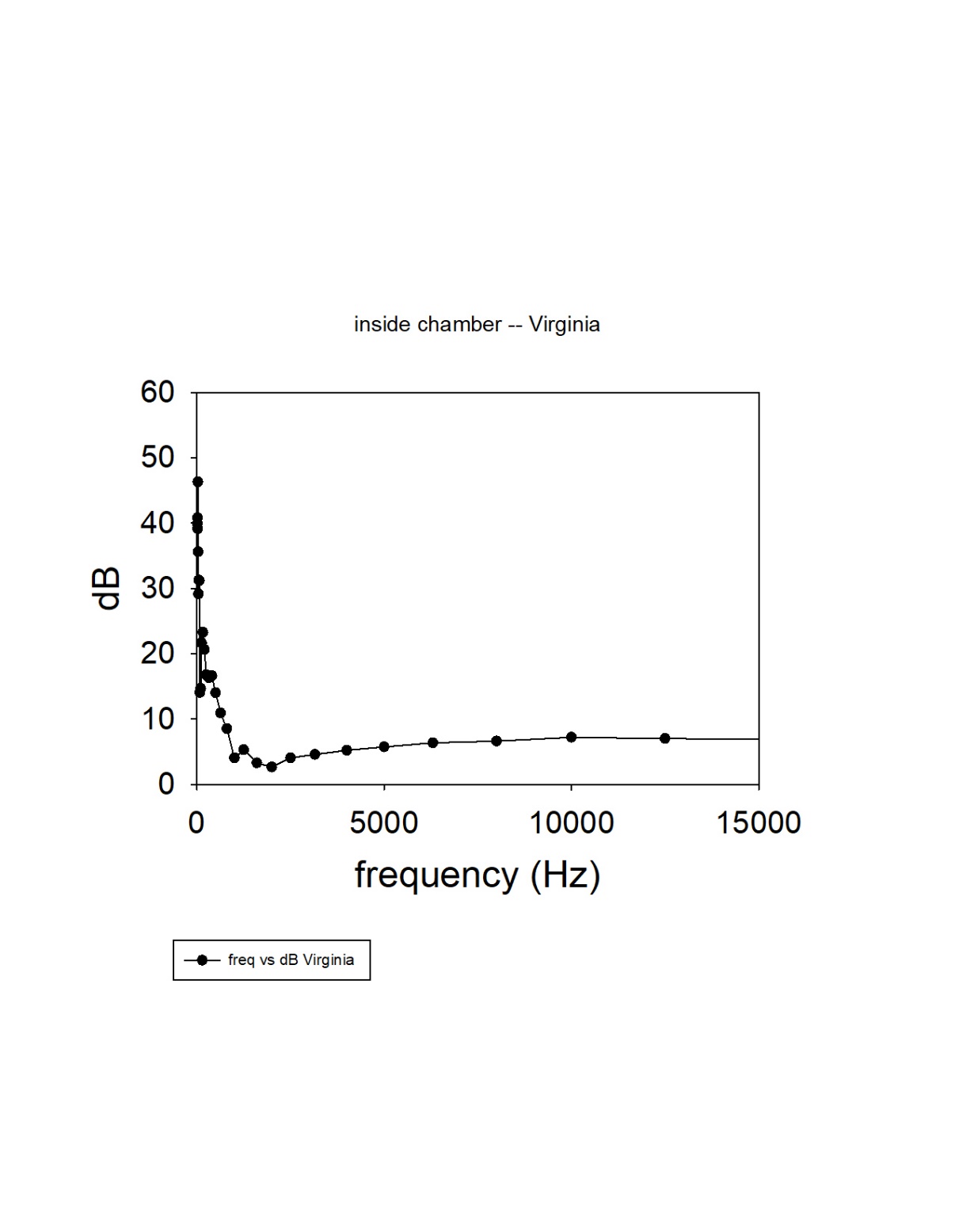


Figure A2. Background sound intensities in the anechoic chamber set up for AEP measurements. These data are from the Wildlife Center of Virginia (04/04/2018).
