# Supplementary material for: Selecting auditory alerting stimuli for eagles on the basis of auditory evoked potentials
Source: Conserv Physiol. 2022 Sep 16;10(1):coac059. doi: 10.1093/conphys/coac059 (PMC9486983; doi:10.1093/conphys/coac059)
Supplement: Web_Material_coac059 [file web_material_coac059.zip › Goller et al.Appx 2 STIMULI table.docx]

**Appendix 2.** Overview of stimuli used to test golden and bald eagle auditory processing.

| Stimulus Group | Stimulus | Duration (ms) | Freq. (kHz) | Amp. Mod. (kHz) | Freq. Mod. (Hz) |
| --- | --- | --- | --- | --- | --- |
| Static Tone | 0.5 kHz tone | 30 | 0.5 |  |  |
|  | 1 kHz tone | 30 | 1 |  |  |
|  | 2 kHz tone | 30 | 2 |  |  |
|  | 3 kHz tone | 30 | 3 |  |  |
|  | 4 kHz tone | 30 | 4 |  |  |
|  | 5 kHz tone | 30 | 5 |  |  |
| Tone Stacks | Harmonic stack | 30 | 1, 2, 3, 4, 5 |  |  |
|  | Mistuned stack | 30 | 1.0, 2.2, 3.3, 3.6, 4.7 |  |  |
|  | Missing fundamental stack | 30 | 1.2, 1.8, 2.4, 3 | 0.6 |  |
| Amplitude Modulation | 1 kHz carrier | 50 | 0.9, 1, 1.1 | 0.1 |  |
| (AM) |  | 50 | 0.6, 1, 1.4 | 0.4 |  |
|  |  | 50 | 0.3, 1, 1.7 | 0.7 |  |
|  | 2 kHz carrier | 50 | 1.9, 2, 2.1 | 0.1 |  |
|  |  | 50 | 1.6, 2, 2.4 | 0.4 |  |
|  |  | 50 | 1.3, 2, 2.7 | 0.7 |  |
|  | 3 kHz carrier | 50 | 2.9, 3, 3.1 | 0.1 |  |
|  |  | 50 | 2.6, 3, 3.4 | 0.4 |  |
|  |  | 50 | 2.3, 3, 3.7 | 0.7 |  |
| Frequency Sweep | Slow up | 50 | 1 to 6 |  |  |
|  | Slow down | 50 | 6 to 1 |  |  |
|  | Fast up | 30 | 1 to 6 |  |  |
|  | Fast down | 30 | 6 to 1 |  |  |
| Frequency Modulation | Shallow, slow | 75 | 2 ± 0.4 |  | 70 |
| (FM) | Deep, slow | 75 | 2 ± 0.7 |  | 70 |
|  | Shallow, fast | 75 | 2 ± 0.4 |  | 110 |
|  | Deep, fast | 75 | 2 ± 0.7 |  | 110 |
