# Supplementary material for: Selecting auditory alerting stimuli for eagles on the basis of auditory evoked potentials
Source: Conserv Physiol. 2022 Sep 16;10(1):coac059. doi: 10.1093/conphys/coac059 (PMC9486983; doi:10.1093/conphys/coac059)
Supplement: Web_Material_coac059 [file web_material_coac059.zip › Goller et al.Appx 3 AEP examples.pptx]

## Slide 1
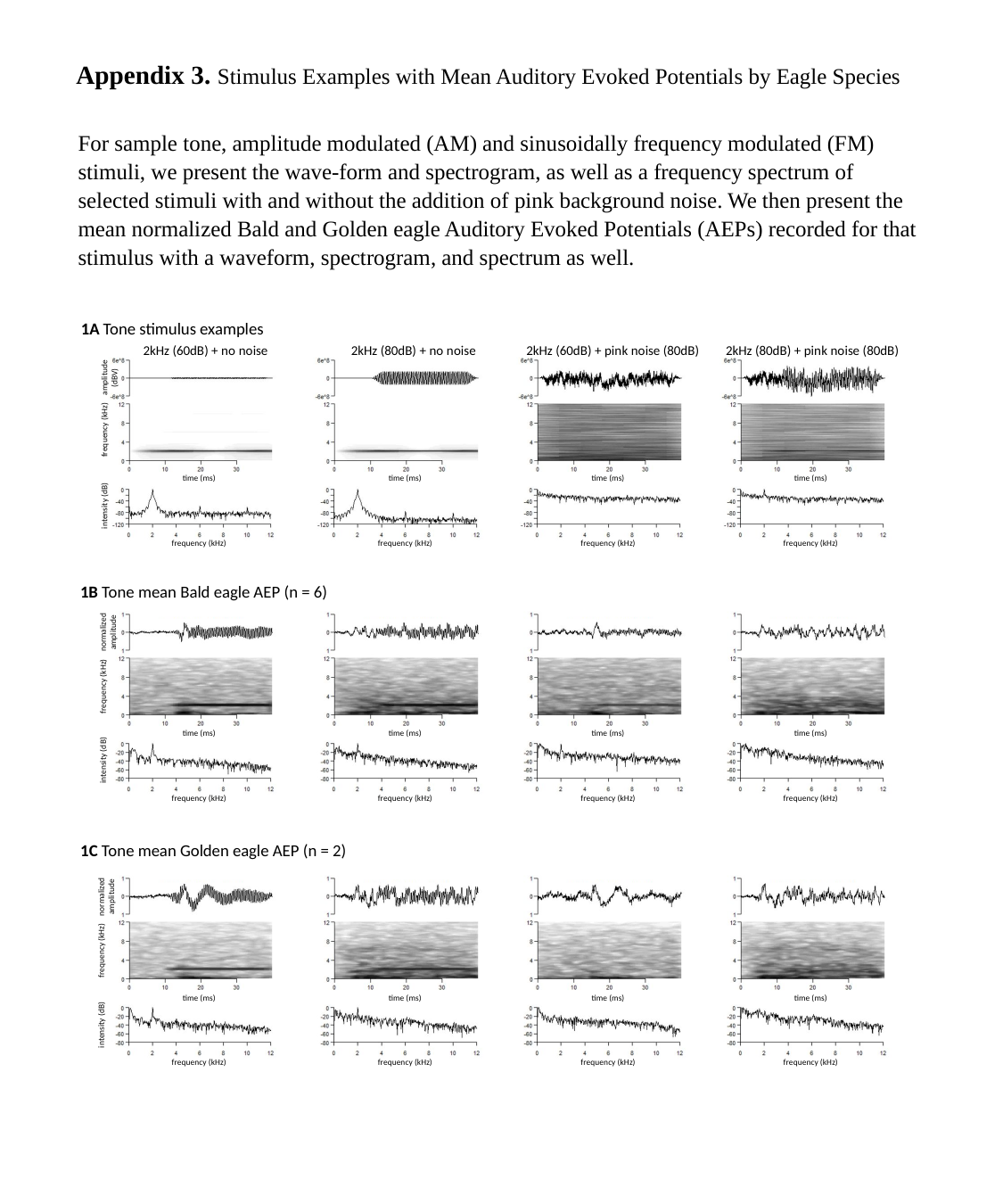

Appendix 3. Stimulus Examples with Mean Auditory Evoked Potentials by Eagle Species
For sample tone, amplitude modulated (AM) and sinusoidally frequency modulated (FM) stimuli, we present the wave-form and spectrogram, as well as a frequency spectrum of selected stimuli with and without the addition of pink background noise. We then present the mean normalized Bald and Golden eagle Auditory Evoked Potentials (AEPs) recorded for that stimulus with a waveform, spectrogram, and spectrum as well.
1A Tone stimulus examples
cv
2kHz (60dB) + no noise
2kHz (80dB) + no noise
2kHz (60dB) + pink noise (80dB)
2kHz (80dB) + pink noise (80dB)
amplitude
(dBV)
frequency (kHz)
time (ms)
time (ms)
time (ms)
time (ms)
intensity (dB)
frequency (kHz)
frequency (kHz)
frequency (kHz)
frequency (kHz)
1B Tone mean Bald eagle AEP (n = 6)
cv
cv
cv
cv
cv
normalized
amplitude
frequency (kHz)
time (ms)
time (ms)
time (ms)
time (ms)
intensity (dB)
frequency (kHz)
frequency (kHz)
frequency (kHz)
frequency (kHz)
1C Tone mean Golden eagle AEP (n = 2)
cv
cv
cv
cv
cv
normalized
amplitude
frequency (kHz)
time (ms)
time (ms)
time (ms)
time (ms)
intensity (dB)
frequency (kHz)
frequency (kHz)
frequency (kHz)
frequency (kHz)

## Slide 2
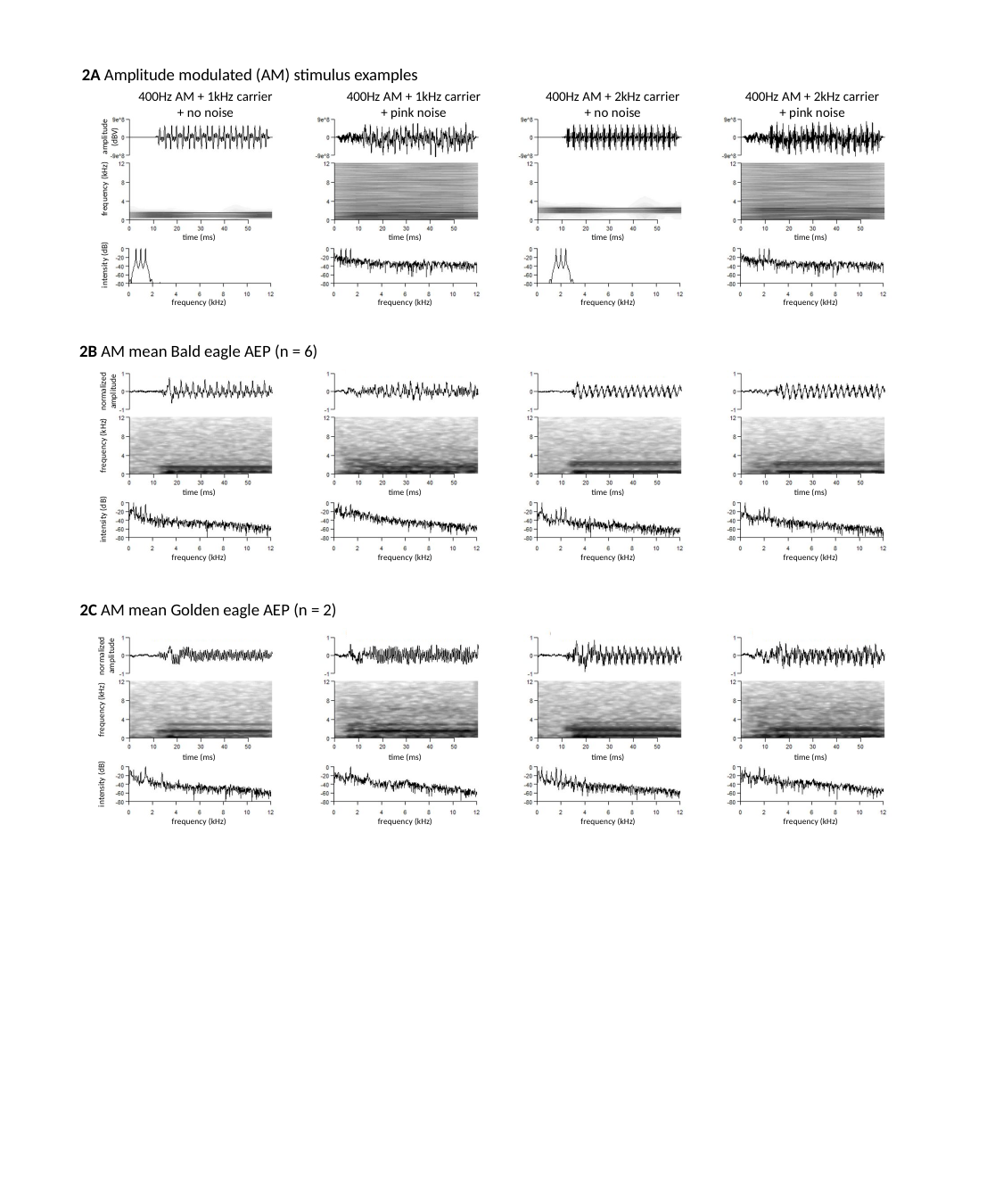

2A Amplitude modulated (AM) stimulus examples
400Hz AM + 1kHz carrier
+ no noise
400Hz AM + 1kHz carrier
+ pink noise
400Hz AM + 2kHz carrier
+ no noise
400Hz AM + 2kHz carrier
+ pink noise
cv
amplitude
(dBV)
frequency (kHz)
time (ms)
time (ms)
time (ms)
time (ms)
intensity (dB)
frequency (kHz)
frequency (kHz)
frequency (kHz)
frequency (kHz)
2B AM mean Bald eagle AEP (n = 6)
cv
cv
cv
cv
cv
normalized
amplitude
frequency (kHz)
time (ms)
time (ms)
time (ms)
time (ms)
intensity (dB)
frequency (kHz)
frequency (kHz)
frequency (kHz)
frequency (kHz)
2C AM mean Golden eagle AEP (n = 2)
cv
cv
cv
cv
cv
normalized
amplitude
frequency (kHz)
time (ms)
time (ms)
time (ms)
time (ms)
intensity (dB)
frequency (kHz)
frequency (kHz)
frequency (kHz)
frequency (kHz)

## Slide 3
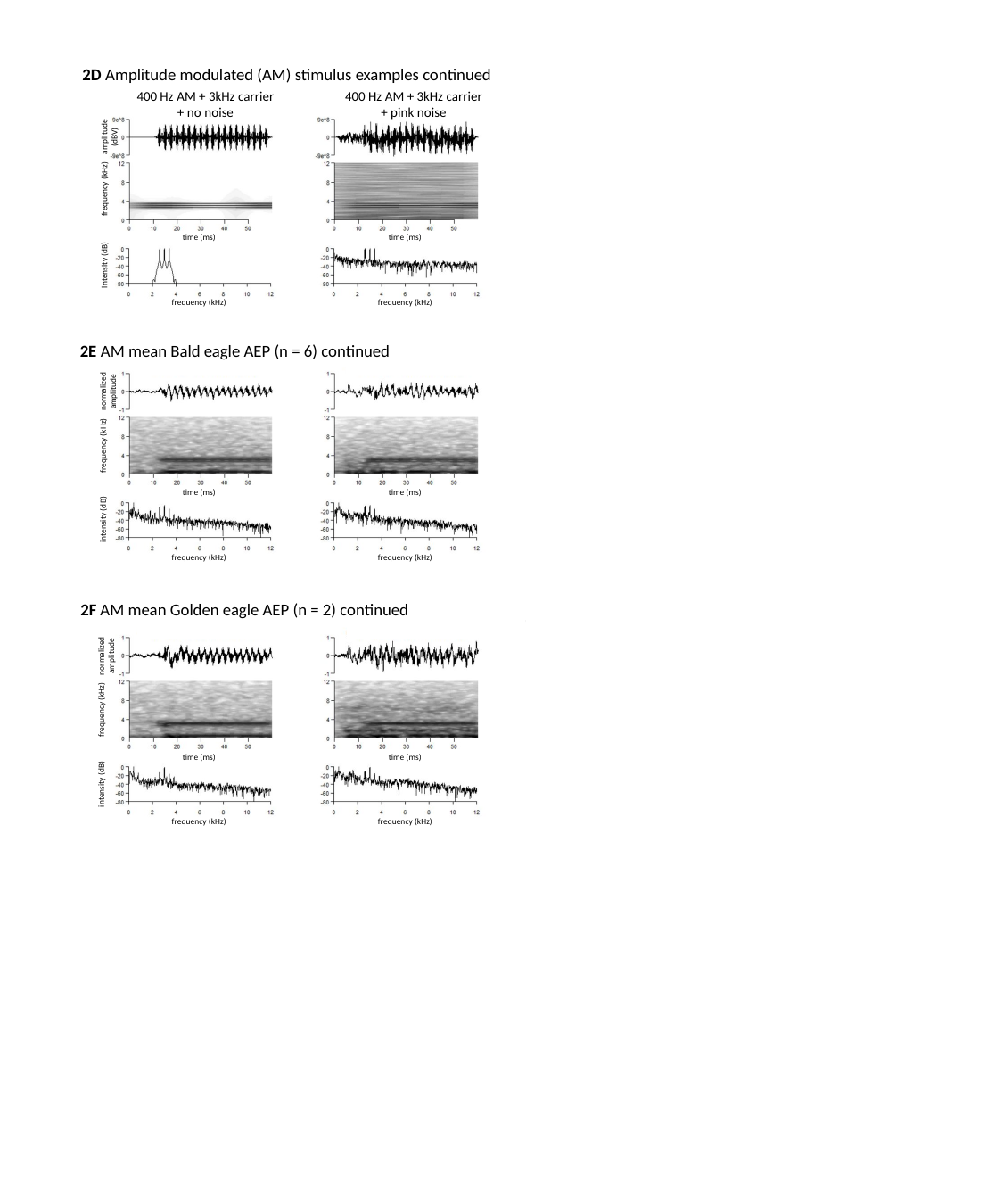

2D Amplitude modulated (AM) stimulus examples continued
400 Hz AM + 3kHz carrier
+ no noise
400 Hz AM + 3kHz carrier
+ pink noise
cv
amplitude
(dBV)
frequency (kHz)
time (ms)
time (ms)
intensity (dB)
frequency (kHz)
frequency (kHz)
2E AM mean Bald eagle AEP (n = 6) continued
cv
cv
cv
normalized
amplitude
frequency (kHz)
time (ms)
time (ms)
intensity (dB)
frequency (kHz)
frequency (kHz)
2F AM mean Golden eagle AEP (n = 2) continued
cv
cv
cv
normalized
amplitude
frequency (kHz)
time (ms)
time (ms)
intensity (dB)
frequency (kHz)
frequency (kHz)

## Slide 4
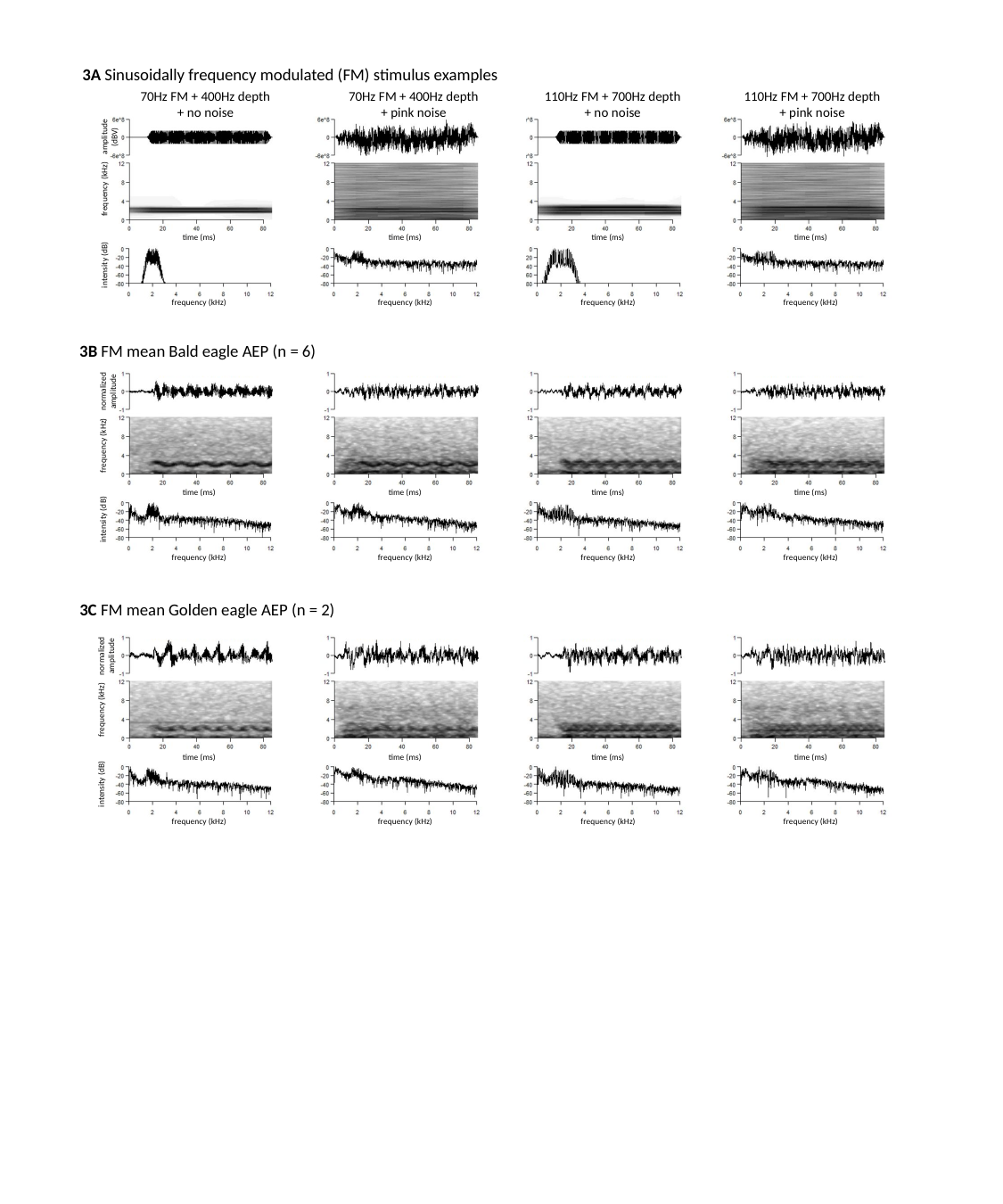

3A Sinusoidally frequency modulated (FM) stimulus examples
70Hz FM + 400Hz depth
+ no noise
70Hz FM + 400Hz depth
+ pink noise
110Hz FM + 700Hz depth
+ no noise
110Hz FM + 700Hz depth
+ pink noise
cv
amplitude
(dBV)
frequency (kHz)
time (ms)
time (ms)
time (ms)
time (ms)
intensity (dB)
frequency (kHz)
frequency (kHz)
frequency (kHz)
frequency (kHz)
3B FM mean Bald eagle AEP (n = 6)
cv
cv
cv
cv
cv
normalized
amplitude
frequency (kHz)
time (ms)
time (ms)
time (ms)
time (ms)
intensity (dB)
frequency (kHz)
frequency (kHz)
frequency (kHz)
frequency (kHz)
3C FM mean Golden eagle AEP (n = 2)
cv
cv
cv
cv
cv
normalized
amplitude
frequency (kHz)
time (ms)
time (ms)
time (ms)
time (ms)
intensity (dB)
frequency (kHz)
frequency (kHz)
frequency (kHz)
frequency (kHz)
